# *Eunoe* Malmgren, 1865 (Annelida, Polynoidae) in the Arctic, North Atlantic, and North Pacific: redescription of the type species and clarification of the genus boundaries

**DOI:** 10.3897/zookeys.1283.168195

**Published:** 2026-07-01

**Authors:** Martha W. Everett, Anna Koroleva, Nicolas Straube, Nataliya Budaeva

**Affiliations:** 1 Shirshov Institute of Oceanology of Russian Academy of Sciences, 36 Nakhimovsky Avenue, Moscow, 117997, Russia Faculty of Biology, Lomonosov Moscow State University Moscow Russia; 2 Faculty of Biology, Lomonosov Moscow State University, Leninskie Gory 1/12, Moscow, 119234, Russia Department of Natural History, University Museum of Bergen, University of Bergen Bergen Norway; 3 Department of Natural History, University Museum of Bergen, University of Bergen, Allégaten 41, 5007, Bergen, Norway Shirshov Institute of Oceanology of Russian Academy of Sciences Moscow Russia

**Keywords:** Integrative taxonomy, lectotype, molecular phylogeny, museomics, new combination, scaleworms, SEM, species delimitation

## Abstract

The scale worm genus *Eunoe* was investigated using an integrative taxonomic approach that combined multilocus DNA (COI, 16S rRNA, ITS2, and 28S rRNA) and detailed morphological data to address persistent uncertainty over generic limits in Polynoidae. Our phylogenetic analyses support *Eunoe* sensu stricto as a clade comprising six species: *Eunoe nodosa* (the type species), *E. oerstedi*, *E. cf. oerstedi* CMC01 (a putative undescribed species), *E. ciliata***comb. nov**. (formerly placed in *Gattyana*), *E. shirikishinai*, and *E. spinicirris*. The remaining fifteen species currently assigned to *Eunoe* were recovered outside *Eunoe* s. str. and were scattered across the inferred phylogeny. Their placement does not allow confident assignment to other polynoid genera. In this study, we redescribe *E. nodosa* and *E. oerstedi*, designate lectotypes for both species, and generate sequence data from the lectotype of *E. oerstedi* using museomics methods. Based on our morphological analysis, five additional species likely belonging to *Eunoe* s. str. are also identified: *E. barbata*, *E. hydroidopapillata*, *E. hozawai*, *E. sentiformis*, and *E. spinosa*. Our findings suggest that *Eunoe* s. str. can be characterized by the presence of macrotubercles on elytra and the dorsal or dorsolateral placement of the anterior pair of eyes. In contrast, the ventral position of the anterior pair of eyes is a diagnostic character of *Gattyana* s. str. We also corroborate earlier evidence for the polyphyly of *Harmothoe*, emphasizing the need for a comprehensive taxonomic revision of generic boundaries in Polynoidae.

## Introduction

*Eunoe* Malmgren, 1865, is a large genus of scale worms from the family Polynoidae, including 49 species recorded worldwide according to the World Register of Marine Species (WoRMS Editorial Board 2025). The genus is currently characterized by fifteen pairs of elytra, stout notochaetae with distinct rows of spinules and rounded tips, and more slender exclusively unidentate neurochaetae with distinct distal rows of spinules (Loshamn 1980; Uschakov 1982; Barnich and Fiege 2010). Although the diagnostic features of *Eunoe* clearly separate it from the related polynoid genera *Harmothoe* Kinberg, 1856, *Leucia* Malmgren, 1867, *Acanthicolepis* McIntosh, 1900, and *Gattyana* McIntosh, 1897, these taxa may appear similar to the untrained eye in general morphology, which can complicate identification (Barnich and Fiege 2010).

Approximately half of the number of species in the genus were described from the Arctic Ocean, the North Atlantic, and the North Pacific (Table 1). Two of these species, *Eunoe nodosa* (M. Sars, 1861) and *E. oerstedi* Malmgren, 1865, both large in size, reaching ≤ 15 cm in length, were commonly reported throughout this region. Their descriptions are based on the syntype series from Svalbard and northern Norway. Another species, *E. senta* (Moore, 1902), was originally described as *Gattyana senta* from McCormick Bay, Greenland, but the type locality was corrected to the Chukchi Sea, Alaska, in a subsequent publication by Moore (1905). This species was transferred to *Eunoe* because it lacked capillary notochaetae, a traditionally diagnostic character of *Gattyana* (Hartman 1939). *Eunoe senta* was reported from Norway by Loshamn (1980) based on a single specimen that is presumably lost (A. Altenburger, pers. comm.), and its presence in the northeastern Atlantic remains questionable. These three species, *E. nodosa*, *E. oerstedi*, and *E. senta*, are frequently confused due to vague original descriptions and similarities in their elytral characters.

**Table 1.** Type localities of *Eunoe* species from the Arctic Ocean, the North Atlantic, and the North Pacific.

**Species**	**Type locality**
**Arctic Ocean**	
*Eunoe bathydomus* (Ditlevsen, 1917)	The Greenlandic part of the Davis Strait
*Eunoe clarki* Pettibone, 1951	Point Barrow, Alaska
*Eunoe nodosa* (M. Sars, 1861)	Norway, Nordkap, Havøysund
*Eunoe oerstedi* Malmgren, 1865	Svalbard, (78.00, 20.00), 46 m, mud
*Eunoe senta* (Moore, 1902)	Chukchi Sea, Alaska
*Eunoe sentiformis* Uschakov, 1958	East Siberian Sea
**North Atlantic**	
*Eunoe hubrechti* (McIntosh, 1900)	United Kingdom Exclusive Economic Zone
*Eunoe laetmogonensis* Kirkegaard & Billett, 1980	Porcupine Seabight
*Eunoe purpurea* Treadwell, 1936	Nonsuch Island, Bermuda
*Eunoe spinulosa* Verrill, 1879	Eastern Canada, Nova Scotia
*Eunoe tritoni* McIntosh, 1900	Færö Channel
**North Pacific**	
*Eunoe barbata* Moore, 1910	Admiralty Inlet, Washington, USA; Santa Cruz, California
*Eunoe depressa* Moore, 1905	Alaska
*Eunoe hozawai* Okuda, 1939	Japan
*Eunoe hydroidopapillata* Rzhavsky & Shabad, 1999	Kamchatka (54.766667, 167.133333)
*Eunoe issunboushi* Jimi, Hookabe, Moritaki, Kimura & Imura, 2021	Kumano Sea, offshore Eastern Honshu, Japan (34.019444, 136.386111). Commensal in the shell of *Microfusus magnifica* occupied by *Parapagurodes doederleini*, the Kumano Sea, 280 m
*Eunoe shirikishinai* Imajima & Hartman, 1964	Japan, Hokkaido
*Eunoe spinicirris* Annenkova, 1937	Northern Sea of Japan
*Eunoe spinosa* Imajima, 1997	Japan
*Eunoe subtruncata* Annenkova, 1937	Sea of Okhotsk, Bolshoy Shantar Island
*Eunoe yedoensis* McIntosh, 1885	Japan

It was a common view in the early studies that *E. nodosa* and *E. oerstedi*, or even all three species, including *E. senta*, should be synonymized (Nordenskiöld 1883; Wesenberg-Lund 1950). Théel (1879) synonymized these species under the name *Polynoe scabra* Theel, 1879, after comparing materials from Norwegian waters and the Kara Sea. Bidenkap (1894) synonymized *E. nodosa* and *E. oerstedi* after examining a large amount of material and finding that many specimens possessed characters of both species. This view was accepted by Fauvel (1913) and Augener (1913, 1928). Ditlevsen (1917) reported continuous transitions between macrotubercles of *E. nodosa*, *E. oerstedi*, and *E. senta* and suggested synonymizing them.

Since the second half of the 20^th^ century, *E. nodosa* and *E. oerstedi* have been considered valid species and were treated as such. Pettibone (1953, 1954, 1963) distinguished them based on the shape of the macrotubercles, “single row near external border, nodular, with roughened tips” in *E. nodosa* and “variable in number, size, position, branched” in *E. oerstedi*, and additional lobes on elytrophores and dorsal tubercles, which are present in *E. nodosa* and absent in *E. oerstedi*. Loshamn (1980) examined a large number of specimens and the type series of both species and confirmed their validity. However, he found great variability in the shape of the macrotubercles and suggested that only characters such as the presence of additional lobes on elytrophores and the shape of chaetae should be used for identification, adding further complexity to correct identification. The observations of Loshamn (1980) were reported in his thesis and were never published in a peer-reviewed journal. Thus, they were largely overlooked by the wider audience. The shape of macrotubercles remained one of the main diagnostic characters for distinguishing these two species in subsequent literature (Barnich and Fiege 2010; Barnich 2011).

Molecular data for both *E. nodosa* and *E. oerstedi* were presented by Carr et al. (2011). Based on the barcode region of the mitochondrial cytochrome *c* oxidase subunit I (COI) gene, two clades were found within *E. nodosa* with Kimura Two-Parameter (K2P) distance of 2.6%—one from the Canadian Arctic (Nunavut, Devon Island) and the other from the Canadian Arctic (Nunavut), the North Pacific (Alaska, Chukchi Sea, and Bering Strait), and the Northwest Atlantic (Newfoundland and Labrador). Two clades were also found within *E. oerstedi* with a distance of 5.5% (K2P)—one from the North Pacific (Alaska, Chukchi Sea) and the other from the Northwest Atlantic (New Brunswick, St. Andrews). However, the study did not include material from the type localities of the *Eunoe* species. Moreover, the authors did not conclude whether these clades within *E. nodosa* and *E. oerstedi* rank on the species level or represent divergent populations within each species.

Further, the phylogenetic placement of the genus *Eunoe* itself remains uncertain. A single specimen labeled as *Eunoe nodosa* (SMNH 118963), which, based on the BOLD Identification System, was a misidentified specimen of *Harmothoe cf. fragilis*, was included in several studies (Norlinder et al. 2012; Gonzalez et al. 2018; Zhang et al. 2018; Bonifácio and Menot 2019; Murray et al. 2025). Gonzalez et al. (2023) included two specimens—the misidentified *H. cf. fragilis* and a specimen from the Indian Ocean labeled as *Eunoe* sp. In a phylogenetic analysis based on COI by Shields et al. (2013), *Eunoe bathydomus* (Ditlevsen, 1917) did not form a monophyletic group with other *Eunoe* species, whereas *E. nodosa* formed a sister clade with *Gattyana ciliata* Moore, 1902, rendering both genera polyphyletic. In the study by Jimi et al. (2021), the phylogenetic analysis based on COI, 16S rRNA, 18S rRNA, and 28S rRNA revealed a well-supported *Eunoe* clade, which comprised five species, including *E. nodosa*, the genus’s type species, misidentified as *E. uniseriata* Banse & Hobson, 1968, MK390764, *E. oerstedi*, *Eunoe spinicirris* Annenkova, 1937, and *Eunoe shirikishinai* Imajima & Hartman, 1964. Notably, *Gattyana ciliata* was also recovered within the *Eunoe* clade (Jimi et al. 2021). However, the newly described species, *Eunoe issunboushi* Jimi, Hookabe, Moritaki, Kimura & Imura, 2021, did not nest within the *Eunoe* clade, leading to the conclusion that the genus *Eunoe* was not monophyletic and required further taxonomic revision.

In the most recent study, Murray et al. (2025) conducted a comprehensive revision of deep-water Australian polynoids and described four new *Eunoe* species: *E. albacauda* Murray, Burghardt, Gunton, Nizar, Nikolic & Wilson, 2025, *E. apicolata* Murray, Burghardt, Gunton, Nizar, Nikolic & Wilson, 2025, *E. benhami* Murray, Burghardt, Gunton, Nizar, Nikolic & Wilson, 2025, and *E. danmurrayi* Murray, Burghardt, Gunton, Nizar, Nikolic & Wilson, 2025. Their molecular analyses based on four markers (COI, 16S rRNA, 18S rRNA, and 28S rRNA) recovered these species as distinct, well-supported clades but confirmed that *Eunoe*, as currently defined, was polyphyletic. However, the authors retained the traditional morphological definition of the genus, emphasizing that the distinction between *Eunoe* and *Harmothoe* was largely arbitrary and that a broader phylogenetic framework for the family Polynoidae was still needed.

In the present study, we aim to clarify the diagnoses of *Eunoe nodosa* and *E. oerstedi* based on examination of their type series, as well as on molecular and morphological data recorded for newly collected material from the Nordic seas and the Russian Arctic. We describe variation in macrotubercle shape and report new morphological characters previously not used in species diagnoses. Lectotypes for both species are designated and described, and sequence data are generated from the lectotype of *E. oerstedi* using museomics methods. Additionally, we perform a phylogenetic reconstruction with nuclear and mitochondrial markers using molecular data currently available for *Eunoe* species, redefining the genetic boundaries of *Eunoe* and clarifying the phylogenetic relationships between *Eunoe*, *Harmothoe*, and *Gattyana*. Furthermore, we examine all *Eunoe* species belonging to the clade supported by molecular phylogenetics, including the type species *E. nodosa*, and morphologically compare them with several *Eunoe* species lacking molecular data, and provide an identification key to *Eunoe* s. str. with illustrations. We further propose new characters for distinguishing *Eunoe* and *Gattyana*.

## Material and methods

### Specimens and study area

We examined the morphology of 424 specimens from 164 collecting sites across a depth range of 0–1925 m (Suppl. material 1: table SS1), with most of the specimens coming from the continental shelf along the Swedish and Norwegian coasts. Molecular data were obtained for 71 specimens (Suppl. material 1: table SS1).

The type series of *Eunoe nodosa* was borrowed from the Natural History Museum, University of Oslo, Norway (**NHMO**); types of *E. oerstedi* were borrowed from the Swedish Museum of Natural History (**SMNH**). Comparative material was borrowed from the collections of the University Museum of Bergen, University of Bergen, Norway (**ZMBN**), the Shirshov Institute of Oceanology, Russian Academy of Sciences, Russia (**IO RAS**), the White Sea Biological Station, Moscow State University, Russia (**WSBS MSU**), the University Museum of the Norwegian University of Science and Technology, Trondheim, Norway (**NTNU-VM**), and the National Museum of Natural History, Smithsonian Institution, USA (**USNM**). Several specimens studied by Carr et al. (2011) were borrowed from the Senckenberg Museum Frankfurt, Germany (**SMF**). Sampling locality details of the studied specimens are given in Suppl. material 1: table SS1, and abbreviated information (registration and specimen number) is provided in the ‘material examined’ sections.

### Molecular analysis

#### Modern samples

Genomic DNA was extracted from the 96% EtOH-fixed samples using the QuickExtract DNA solution (Epicentre). A small part from the middle of the body (5–10 segments) for small specimens and a part of a parapodium for large specimens was cut off and placed in 100–150 μL QuickExtract solution and treated at 65 °C for 45 min, followed by 2 min at 95 °C in a thermal cycler.

Two mitochondrial (fragments of COI, 16S rRNA) and two nuclear (complete ITS2 and a fragment of 28S rRNA) markers were amplified using primers and protocols shown in Suppl. material 1: table SS2. PCR was performed in two laboratories (Molecular ecology lab at the Institute of Oceanology, Russian Academy of Sciences, and DNA lab at the University of Bergen, Norway), using two different protocols. In the first protocol, each PCR reaction contained 14.2 μL ddH2O, 0.4 μL of each primer (10 μM), 1 μL of template DNA, and 4 μL of a five-fold solution of Screen Mix-HS (Eurogen) for a total reaction volume of 20 μL. Amplification success of the PCRs was checked using electrophoresis on a 1% agarose gel stained with GelRed Nucleic Acid Stain. Each PCR product (1–4 μL) was purified by adding 50 μL of a mixture containing 1.5 μL NH4Ac 5M, 43.8 μL EtOH 96%, and 4.7 μL ddH2O. Each sample was mixed by vortexing, incubated at room temperature for 20 min, and then centrifuged at 14,000 rpm for 20 min. The supernatant was removed by inverting the tubes over a sink. The precipitate was mixed with 1 μL of primer (4 μM), dried in a thermostat, and sent for sequencing to Syntol (Moscow, Russia).

In the second protocol, each PCR reaction contained 17.35 μL ddH2O, 2.5 μL buffer (10×), 2 μL nucleotide mix, 1 μL of each primer (10 µM), 0.15 μL Taq DNA (Takara Taq, concentration of 5 U/μL), and 1 μL of template DNA in a mixture totaling 25 μL. Amplified PCR products were analyzed by electrophoresis on a 1% agarose gel stained with GelRed Nucleic Acid Stain and then sent to Macrogen Inc. (Amsterdam, the Netherlands) for purification and bidirectional sequencing.

#### Museomics

To extract DNA from the presumably ethanol-fixed type specimen of *Eunoe oerstedi*SMNH 2391 collected in 1861, we followed the Guanidine DNA extraction protocol as detailed in Straube et al. (2021). 50 mg of pharyngeal tissue was used for extraction, flanked by two negatives. The final extract was measured with a Qubit Fluorometric quantification® using the high-sensitivity DNA detection kit.

Thereafter, single-stranded DNA libraries were constructed following the protocol by Gansauge et al. 2017, adding 5.14 ng of DNA from the initial extract. The DNA library was then sequenced targeting 5 million 75-basepair sequencing reads on an Illumina MiniSeq sequencing instrument using a mid-output 75-bp sequencing kit and custom sequencing and indexing primers as described in Paijmans et al. (2017). All laboratory steps and sequencing were performed at the University Museum of Bergen’s DNA laboratory.

After sequencing, raw reads were demultiplexed using Illumina’s bcl2fastq software. Raw reads were then trimmed to remove adapters and low-quality bases using cutadapt v.1.16 (Martin 2011), adopting the settings as in Straube et al. (2021) and Trimmomatic v.0.39 (Bolger et al. 2014). Quality control for both raw and trimmed reads was assessed with FastQC (Andrews 2010). Trimmed reads were assembled using SPAdes v.3.15.5 (Bankevich et al. 2012). To identify ribosomal markers, the resulting contigs were queried using BLAST search (Altschul et al. 1990) against known nuclear ribosomal sequences of *E. oerstedi* and other polynoids. This approach yielded several contigs containing the ITS2–28S rRNA region and fragments of the 18S rRNA gene. The relevant contigs were manually extracted from the assembly and combined in MEGA 7.0 (Kumar et al. 2016) for further alignment.

For the mitochondrial markers (COI and 16S rRNA), trimmed reads were assembled with MIRA v.4.0.2 (Chevreux et al. 1999) and subsequently mapped in Mitobim v.1.9.1 (Hahn et al. 2013) for each gene using sequences COI and 16S rRNA from the specimen *E. oerstedi* voucher ZMBN 159262 (BOLD process ID SCWO056-24) as the reference.

The resulting consensus sequences for the mitochondrial (COI, 16S rRNA) and nuclear ribosomal (ITS2, 28S rRNA) markers were then added to their respective datasets and aligned as described in the following section.

### Phylogenetic analysis

**Figure 1.**
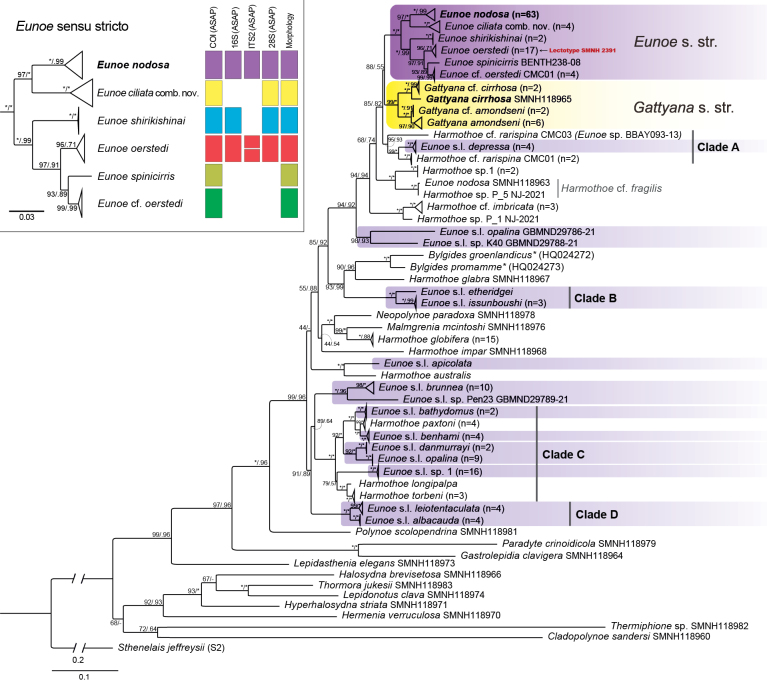
Maximum-likelihood tree inferred from the concatenated COI, 16S rRNA, ITS2, and 28S rRNA dataset. Node support is given as UFBoot/BPP; */* = 100/1; “–” denotes nodes not recovered in the Bayesian inference tree. Numbers in parentheses indicate the number of specimens (n) analyzed in the collapsed clades. Capital letters label clades discussed in the text. Species delimitation within *Eunoe* s. str. inferred by ASAP and morphology is shown in the inset. White spaces indicate missing data. Collapsed clades within *Eunoe* s. str. represent species-level groups. Type species names of *Eunoe* and *Gattyana* are shown in bold. *Bylgides* specimens are marked with an asterisk as potentially misidentified (HQ024272 = *B. elegans*; HQ024273 = *B. annenkovae*; unpublished data).

Molecular data (COI, 16S rRNA, ITS2, and 28S rRNA) were obtained for 62 specimens of *Eunoe*, one specimen of *Gattyana*, seven specimens of *Harmothoe*, and one specimen of *Sthenelais* Kinberg, 1856 (Sigalionidae) used as an outgroup (Suppl. material 1: table SS1). In addition, COI, 16S rRNA, and 28S rRNA sequences of *Eunoe*, *Harmothoe*, *Gattyana*, *Bylgides* Chamberlin, 1919, *Neopolynoe paradoxa* (Anon, 1888), *Malmgrenia mcintoshi* (Tebble & Chambers, 1982), *Paradyte crinoidicola* (Potts, 1910), *Gastrolepidia clavigera* Schmarda, 1861, *Lepidasthenia elegans* (Grube, 1840), *Halosydna brevisetosa* Kinberg, 1856, *Thormora jukesii* Baird, 1865, *Lepidonotus clava* (Montagu, 1808), *Hyperhalosydna striata* (Kinberg, 1856), *Hermenia verruculosa* Grube, 1856, *Thermiphione* Hartmann-Schröder, 1992, and *Cladopolynoe sandersi* (Pettibone, 1985) were obtained from GenBank and BOLD. These included sequences associated with Nygren et al. (2011), Struck et al. (2005, 2007), Carr et al. (2011), Norlinder et al. (2012), Shields et al. (2013), Gonzalez et al. (2017, 2018, 2023), Jimi et al. (2021), Cowart et al. (2022), and Maxwell et al. (2022); the taxon list, accession numbers, voucher information, and original references are provided in Suppl. material 1: table SS1. Sequences were manually edited in CodonCode Aligner (CodonCode Corporation, www.codoncode.com), aligned for each locus separately using the online version of MAFFT with the E-ins-i strategy, and then manually curated in MEGA 7.0 (Kumar et al. 2016). COI sequences were translated into amino acids in MEGA 7.0 (Kumar et al. 2016) to check for stop codons. Alignments of four genes were concatenated in MEGA 7.0 (Kumar et al. 2016). The maximum-likelihood (ML) analysis of the concatenated dataset was run in IQ-TREE 2 (Nguyen et al. 2015) using the usegalaxy.eu server (Minh et al. 2020). The charsets and the best-fitting substitution models were selected with ModelFinder implemented in IQ-TREE (Kalyaanamoorthy et al. 2017). The following models were applied to the following partitions: TIM3e+I+R3 for the first codon position of COI (COIcod1), F81+F+I+R2 for the second codon position (COIcod2), TIM2+F+G4 for the third codon position (COIcod3), TIM2+F+I+G4 for 16S rRNA (S16), TPM2u+F+G4 for ITS2 (ITS2), and TN+F+I+R2 for 28S rRNA (S28) (Chernomor et al. 2016). Node support was assessed using 10,000 ultrafast bootstrap replicates (UFBoot) (Hoang et al. 2018) and the SH-like approximate likelihood ratio test (SH-aLRT) (Guindon et al. 2010). UFBoot values are reported in Fig. 1, whereas SH-aLRT support values are provided in the Supplementary Material (Suppl. material 2: fig. S1). Bayesian inference (BI) was performed in MrBayes v.3.2 (Ronquist et al. 2012) using two independent runs with eight Markov chains each for 5 million generations (temp = 0.1), sampling every 2,000 generations. The first 25% of samples were discarded as burn-in. The charsets and best-fitting substitution models were selected with ModelFinder implemented in IQ-TREE 2 using the usegalaxy.eu server (Minh et al. 2020) specifically to guide model choice for the Bayesian analysis (Kalyaanamoorthy et al. 2017), and then implemented in MrBayes as GTR+I+G (COIcod1, 16S, 28S), HKY+I+G (COIcod2), GTR+G (COIcod3), and HKY+G (ITS2) (Hasegawa et al. 1985). Convergence and mixing were assessed using the average standard deviation of split frequencies (ASDSF < 0.03) and effective sample sizes (ESS > 200) in Tracer v.1.7.1 (Rambaut et al. 2018). The resulting ML tree was visualized in FigTree 1.4.4 (http://tree.bio.ed.ac.uk/software/figtree/) and edited with Inkscape 1.1 (https://inkscape.org/) (Fig. 1).

### Species delimitation

To delineate putative species within the group recognized by the phylogenetic analyses as the *Eunoe* sensu stricto clade, we applied Assemble Species by Automatic Partitioning (ASAP) (Puillandre et al. 2021) separately to each marker (COI, 16S rRNA, ITS2, and 28S rRNA). Analyses were run via the SPART/ASAP web interface (https://spartexplorer.mnhn.fr/delimitation) using default settings (Suppl. material 2: file S1A–D).

### Haplotype network analysis

**Figure 2.**
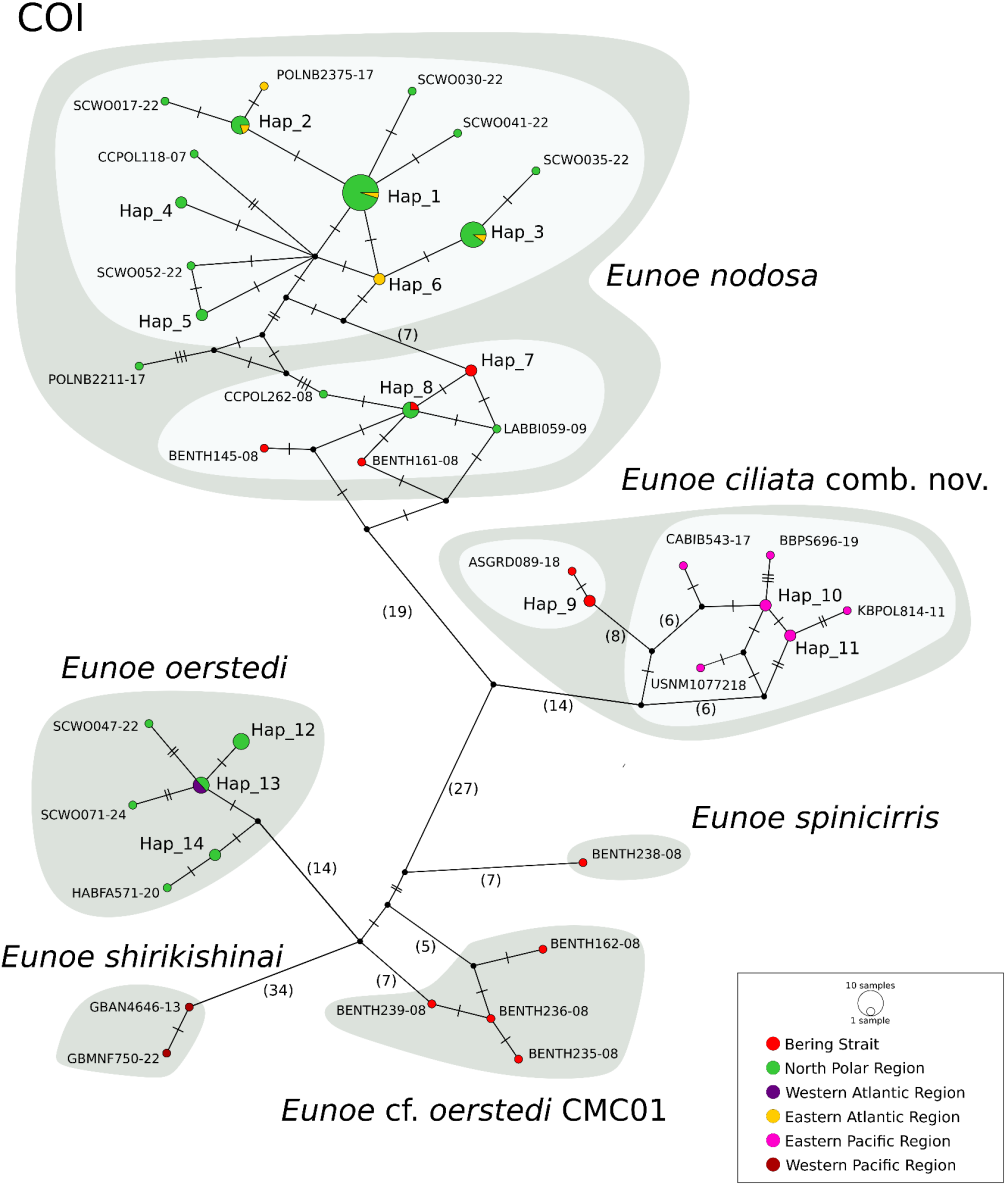
Haplotype network for *Eunoe* sensu stricto based on COI sequence data. The number of mutational steps equal to or fewer than three is represented by perpendicular lines on the connecting lines between haplotypes. The number of mutational steps greater than three is indicated by numbers near the lines. The size of each circle is proportional to the number of sequences, colors indicate geographic regions, and small black circles indicate inferred intermediate haplotypes not observed in the dataset. The red color (Bering Strait) represents the boundary between the North Polar and Pacific regions. Pale grey areas within *E. nodosa* and *E. ciliata* comb. nov. represent haplogroups with distinct geographical distributions.

**Figure 3.**
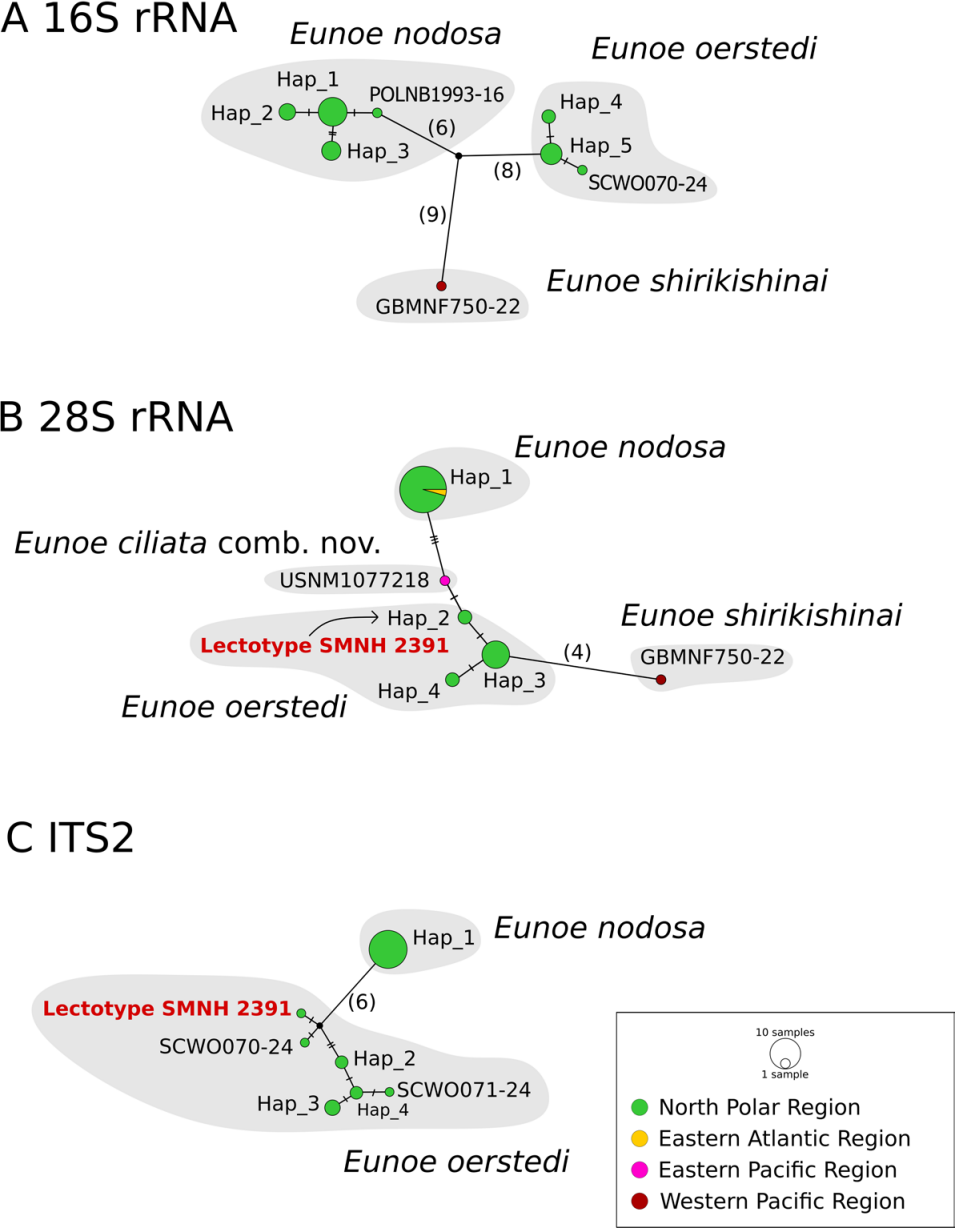
Haplotype network for *Eunoe* sensu stricto based on 16S rRNA sequence data (**A**), 28S rRNA sequence data (**B**), and ITS2 sequence data (**C**). The number of mutational steps equal to or fewer than three is represented by perpendicular lines on the connecting lines between haplotypes. The number of mutational steps greater than three is indicated by numbers near the lines. The size of each circle is proportional to the number of sequences, colors indicate geographic regions, and small black circles indicate inferred intermediate haplotypes not observed in the dataset.

The diversity and relationships among COI, 16S rRNA, ITS2, and 28S rRNA haplotypes for the group recognized by the phylogenetic analyses as the *Eunoe* sensu stricto clade were determined by constructing Templeton–Crandall–Sing (TCS) haplotype networks (Clement et al. 2002) in PopART v.1.7 (Leigh and Bryant 2015) (Figs 2, 3). The following geographical regions were coded as traits based on the subdivision of the Arctic region provided by Mironov (2013): the Eastern and the Western Atlantic regions, the Eastern and the Western Pacific regions, the North Polar region, and the boundary between the North Polar region and the Pacific regions. The latter was coded as a separate geographical unit (trait) because two species in our dataset, *Eunoe oerstedi* CMC01 and *Eunoe spinicirris*, were found only in the Bering Strait. This limited dataset prevented us from determining whether these species inhabit only one of the adjacent regions, both regions, or are restricted to the boundary separating the North Polar region from the Pacific regions.

### Morphological examination

**Figure 4.**
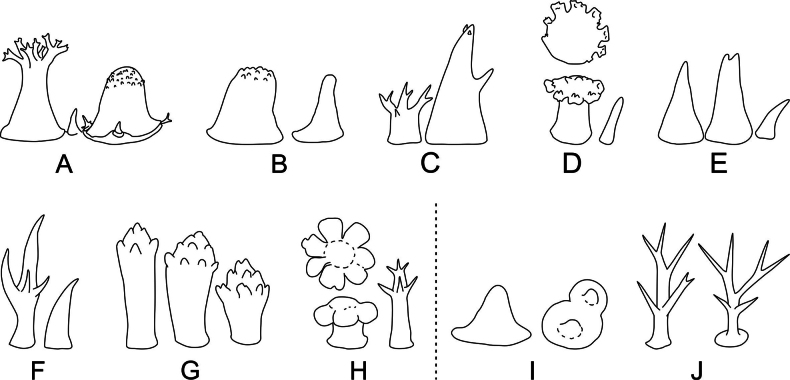
Typical elytral macrotubercles of *Eunoe* sensu stricto (**A–H**) and *Eunoe* sensu lato (**I–J**): **A**. *Eunoe nodosa*, apically arborescent macrotubercles (dichotomous branchlets) and semiglobose to cylindrical macrotubercles with nodular papillae on apex; **B**. *E. ciliata* comb. nov., semiglobose to cylindrical macrotubercles with nodular papillae on apex and conical macrotubercles with blunt, rounded tips; **C**. *E. oerstedi*, apically arborescent macrotubercles (conical, non-dichotomous branchlets) and horn-like macrotubercles; **D**. *E. cf. oerstedi* CMC01, cylindrical macrotubercles with flattened, crown-like tip with undulate margin (appearing as coronate discs in top view); **E**. *E. spinicirris*, spines, occasionally bifid; **F**. *E. shirikishinai*, spiniform macrotubercles, including furcate forms, and simple spiniform macrotubercles; **G**. *E. barbata*, cylindrical to slightly clavate macrotubercles, apically coronate with small branches with rounded tips, forming crenulate apex; **H**. *E. sentiformis*, cylindrical macrotubercles, ending distally in rosette-shaped apex with strongly lobate margin and cylindrical macrotubercles with dentate crown at tip; **I**. E. s. l. depressa, sessile, low mammilliform macrotubercles; **J**. E. s. l. senta, antler-like, long-branched macrotubercles with distally pointed branchlets.

**Figure 5.**
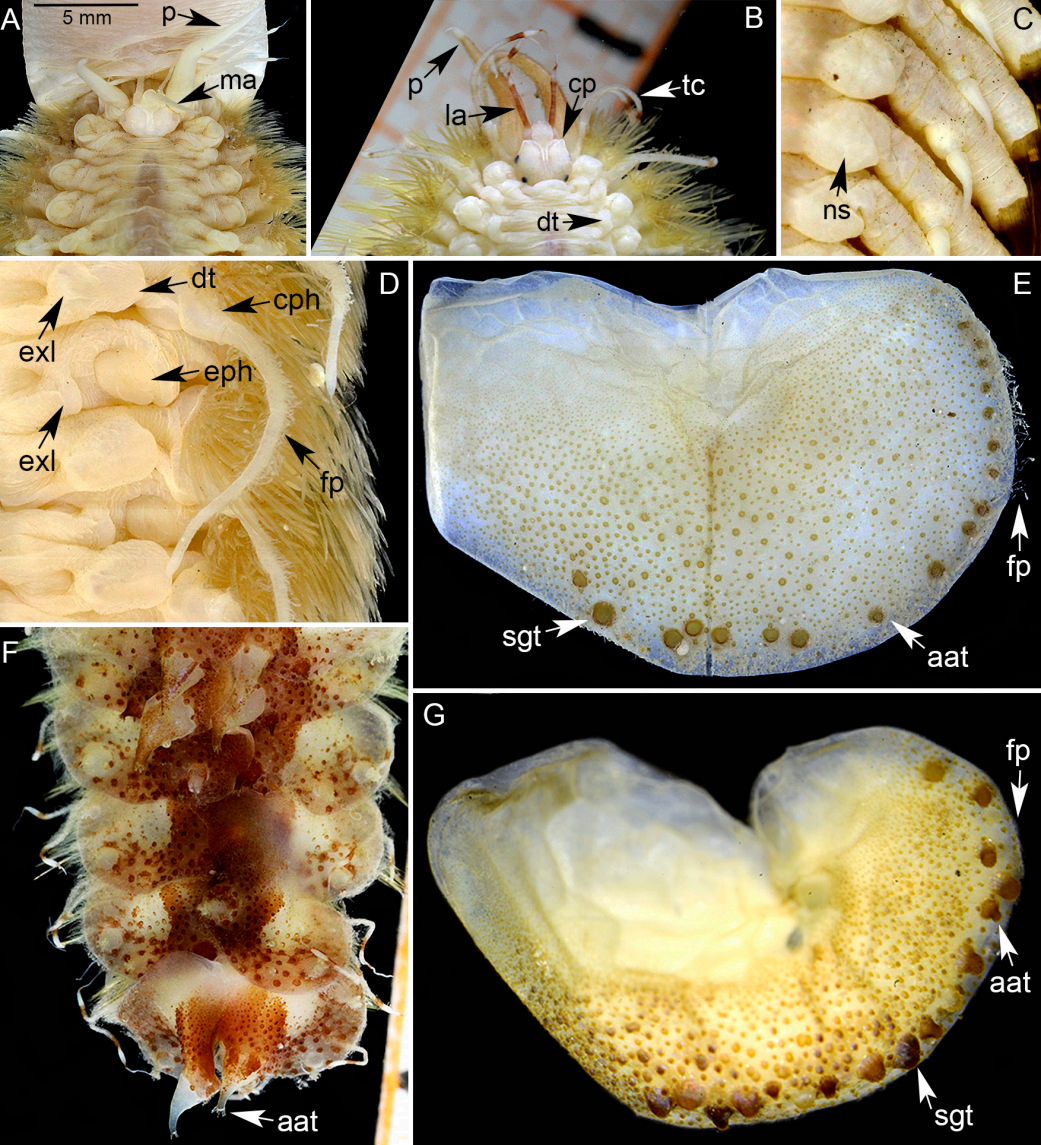
*Eunoe nodosa*, light microscopy of body and elytra. (**A, C–E**) Lectotype NHMO IN-5334 and NHMO IN-5335 (Barents Sea, near Nordkap); **A**. Prostomium, dorsal view; **B**. Prostomium, dorsal view, ZMBN 150373 (Greenland, Disco Island); **C**. Nephridial papillae covered by nephridial sac, ventral view; **D**. Extra lobes on cirrophores, dorsal tubercles, and elytrophores; cirrophore with dorsal cirri covered by filiform papillae, dorsal view; **E**. Elytron, dorsal view; **F**. Elytra, dorsal view, ZMBN 150381 (Norwegian Sea); **G**. Elytron, dorsal view, USNM 21611 (Alaska). Ruler unit: 1 mm. Abbreviations: aat – apically arborescent tubercle, cp – cephalic peaks, cph – cirrophore, dt – dorsal tubercle, eph – elytrophore, exl – extra lobe, fp – filiform papillae, la – lateral antenna, ma – median antenna, ns – nephridial sac, p – palp, sgt – semiglobose macrotubercles, tc – tentacular cirri.

**Figure 6.**
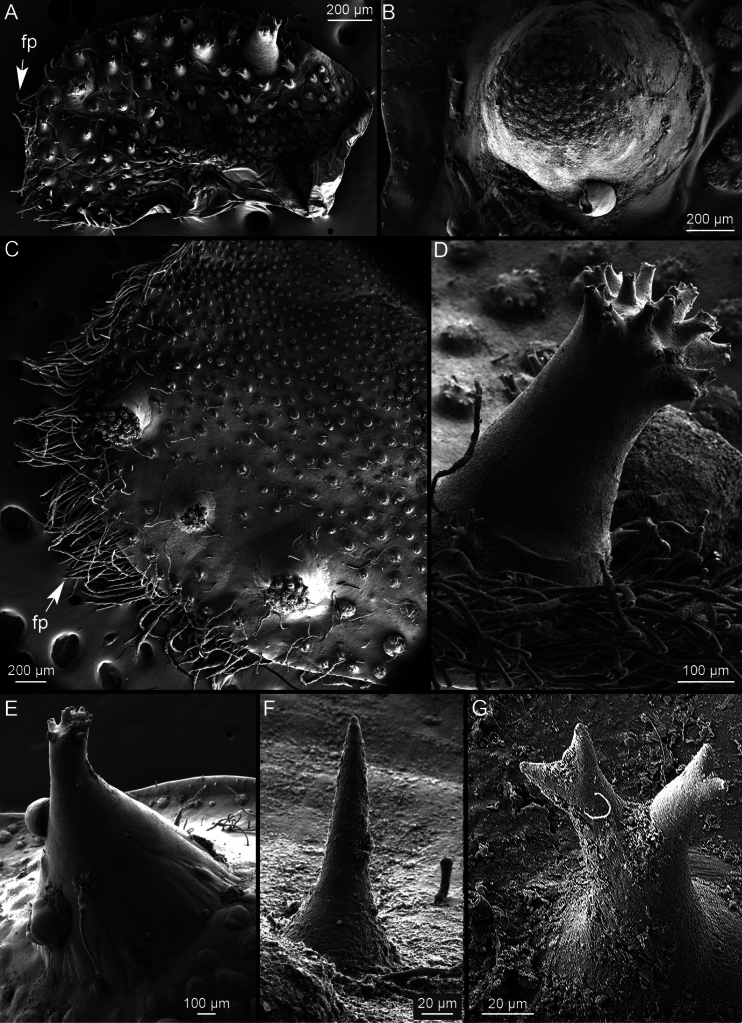
*Eunoe nodosa*, SEM of elytra from mid-body. **A**. Elytron, dorsal view, ZMBN 150389 (small specimen); **B**. Semiglobose macrotubercle with nodular papillae apically, ZMBN 150363 (large specimen); **C**. Part of elytron with apically arborescent macrotubercles and filiform papillae on margin, ZMBN 150373 (medium specimen); (**D–F**) ZMBN 149383: **D**. Apically arborescent macrotubercles and small round macrotubercles in background; **E**. Elongated apically arborescent macrotubercle; **F**. Spine; **G**. Bifid spine, ZMBN 150363 (large specimen). Abbreviations: fp – filiform papillae.

**Figure 7.**
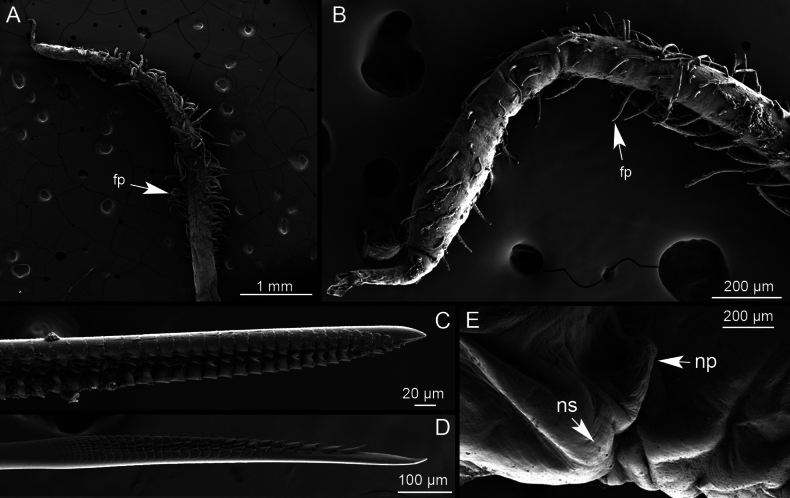
*Eunoe nodosa*, SEM of parapodia from mid-body. **A**. Lectotype NHMO IN-5334 (large specimen); **B–D**. ZMBN 150361 (large specimen). **A, B**. Dorsal cirrus; **C**. Notochaeta with subacute tip; **D**. Neurochaeta; **E**. Nephridial papilla. Abbreviations: fp – filiform papillae, np – nephridial papilla, ns – nephridial sac.

**Figure 8.**
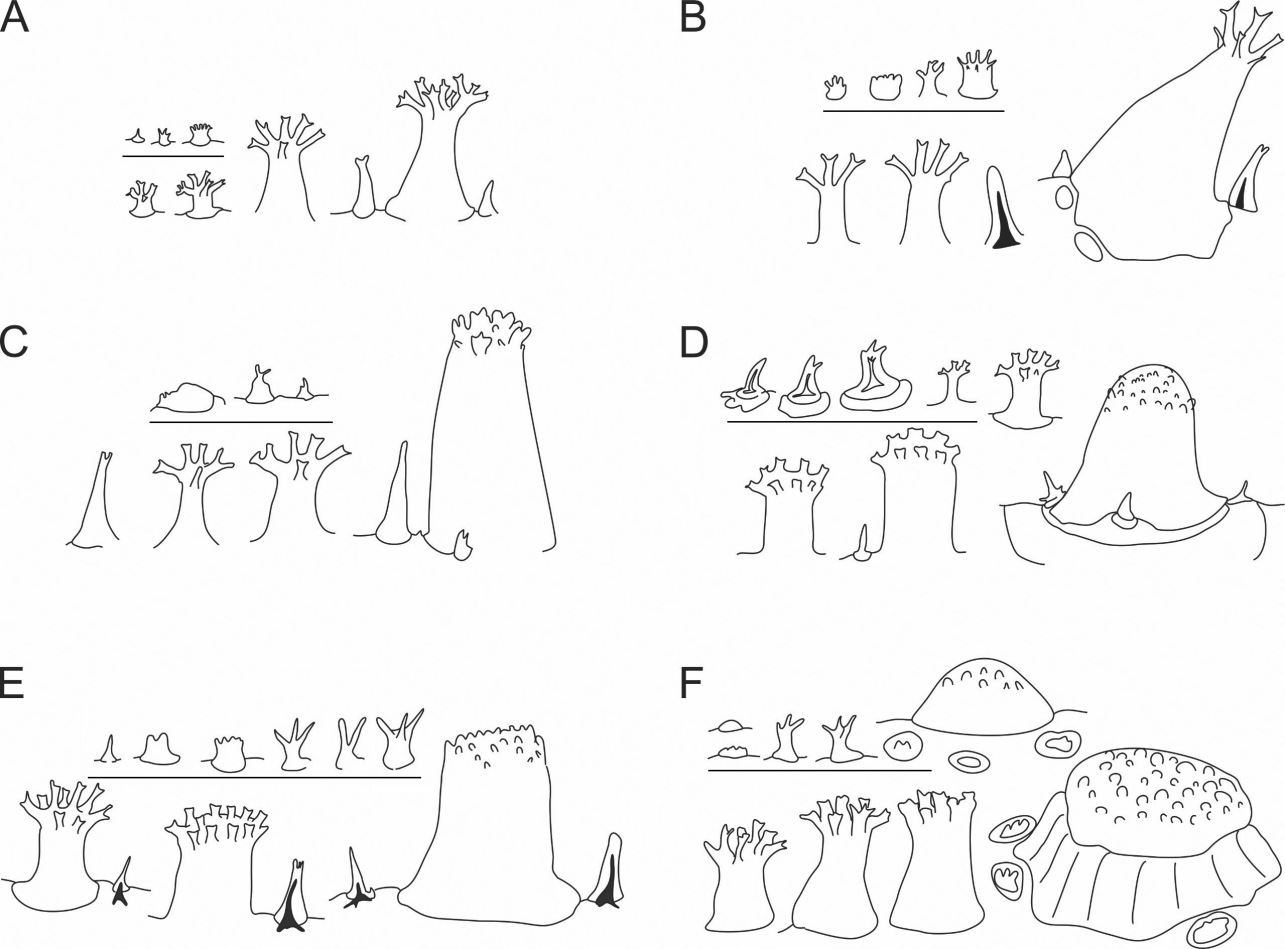
Variation in elytral micro- and macrotubercles in *Eunoe nodosa*. In each panel, microtubercles are indicated by black horizontal bars. Some microtubercles show a dark central core under light microscopy. Specimens are grouped by body length: <5 mm (**A, B**), 5–10 mm (**C, D**), and >10 mm (**E, F**). **A**. ZMBN 150378, macrotubercles apically arborescent; **B**. ZMBN 150381, elongated macrotubercles, apically arborescent; **C**. ZMBN 150371, cylindrical macrotubercles with apically arborescent tips; **D**. ZMBN 150373, apically arborescent macrotubercles and cylindrical macrotubercles with apical nodular papillae; **E**. ZMBN 150384, apically arborescent macrotubercles and cylindrical macrotubercles with apical nodular papillae; **F**. ZMBN 150383, apically arborescent macrotubercles and semiglobular macrotubercles covered with nodular papillae. Maximum macrotubercle height 1.3 mm (**B**).

**Figure 9.**
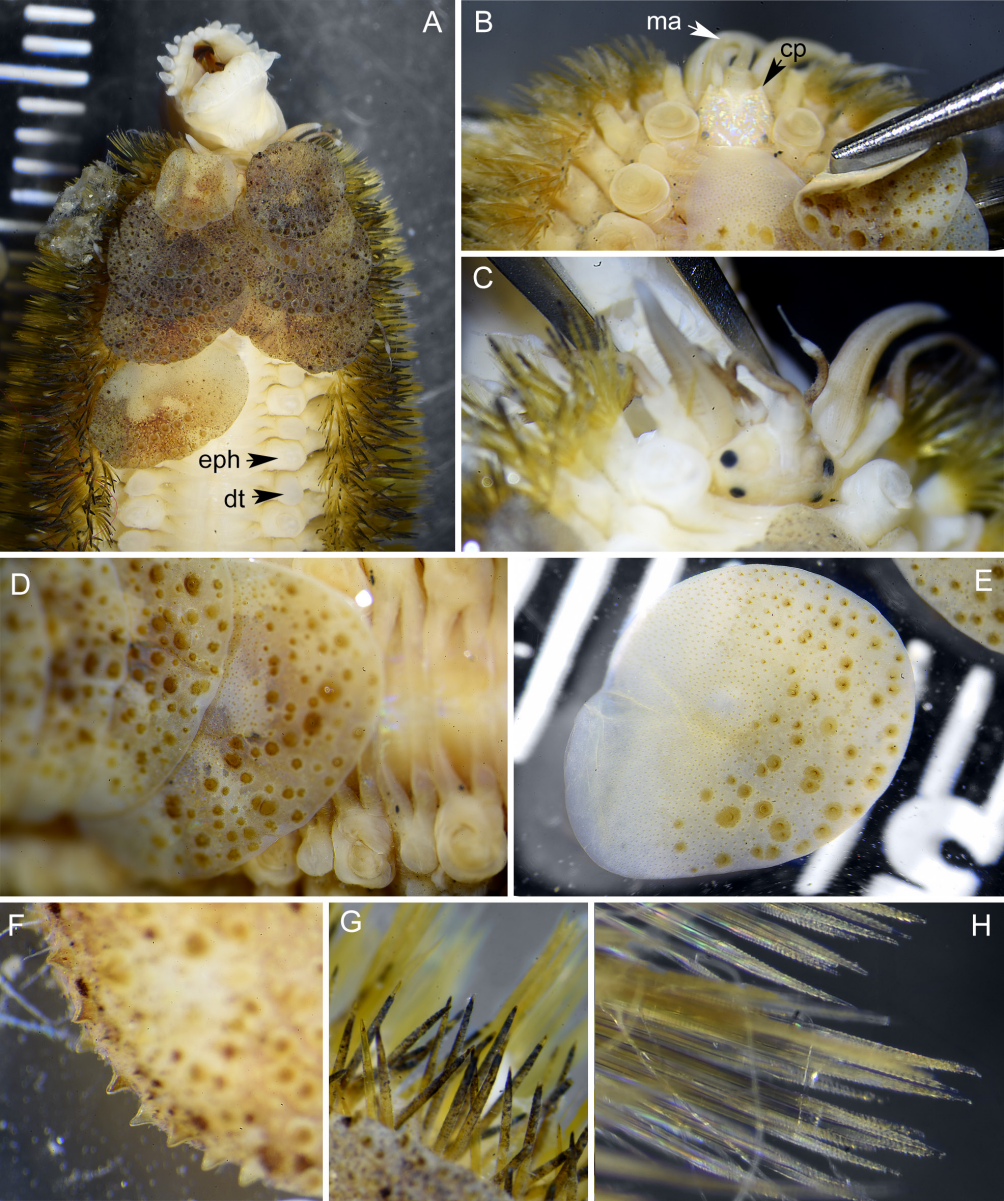
Eunoe s. l. depressa, light microscopy. USNM 1512664 (Alaska) (**A, C, F–H**), paratype USNM 5590 (Alaska) (**B, D**), holotype USNM 5517 (Alaska) (**E**). **A**. Anterior part, with everted pharynx, dorsal view; **B, C**. Prostomium, dorsal view; **D**. Elytra, dorsal view; **E**. Elytron, dorsal view; **F**. Low, sessile mammilliform macrotubercles, dorsal view; **G**. Notochaetae; **H**. Neurochaetae. Ruler unit: 1 mm. Abbreviations: cp – cephalic peaks, dt – dorsal tubercle, eph – elytrophore, ma – median antenna.

**Figure 10.**
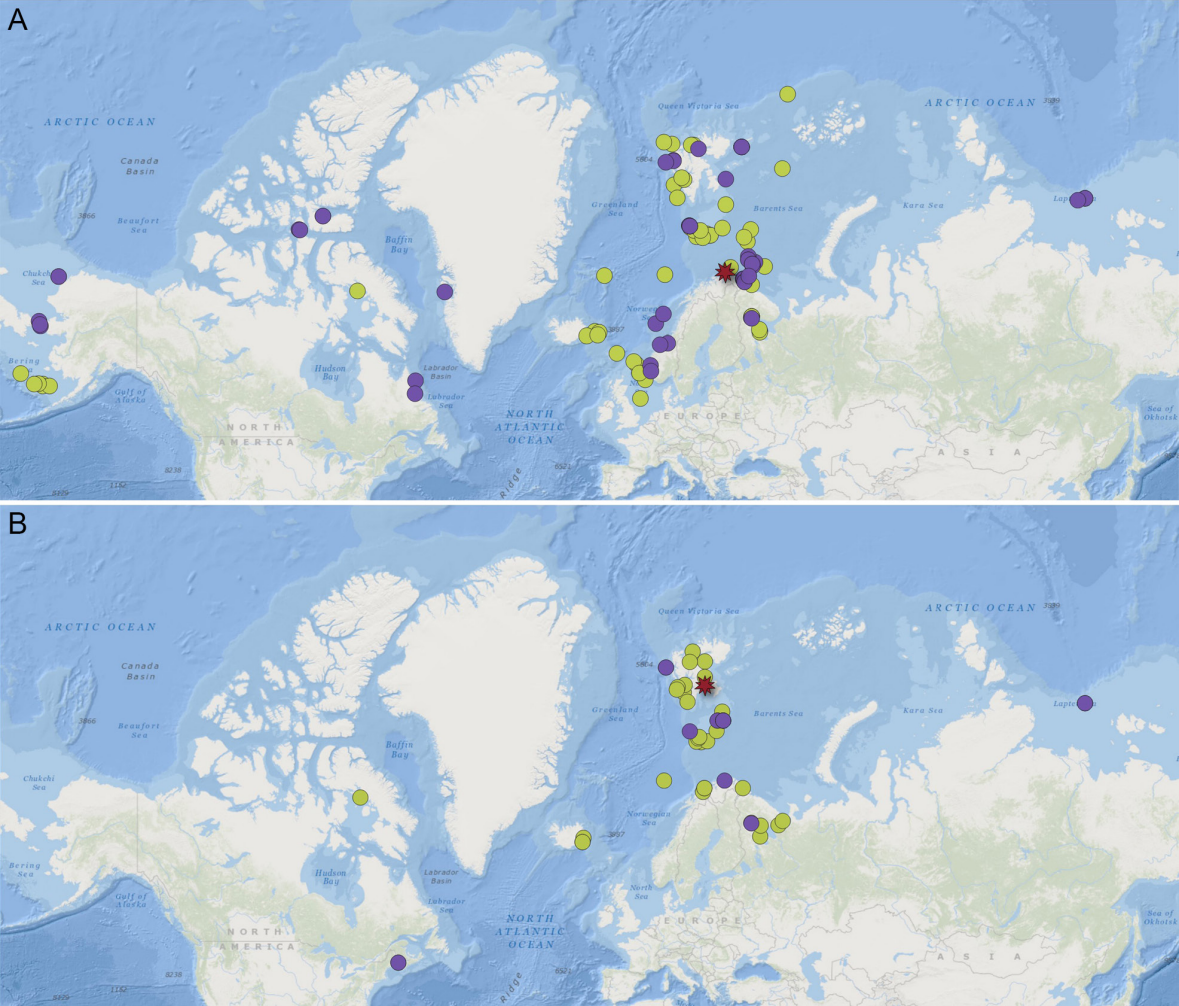
**A**. Distribution of *Eunoe nodosa*; **B**. Distribution of *Eunoe oerstedi*. Red stars – type localities, purple circles – localities of specimens with examined morphology and molecular data, green circles – localities of specimens with examined morphology only.

**Figure 11.**
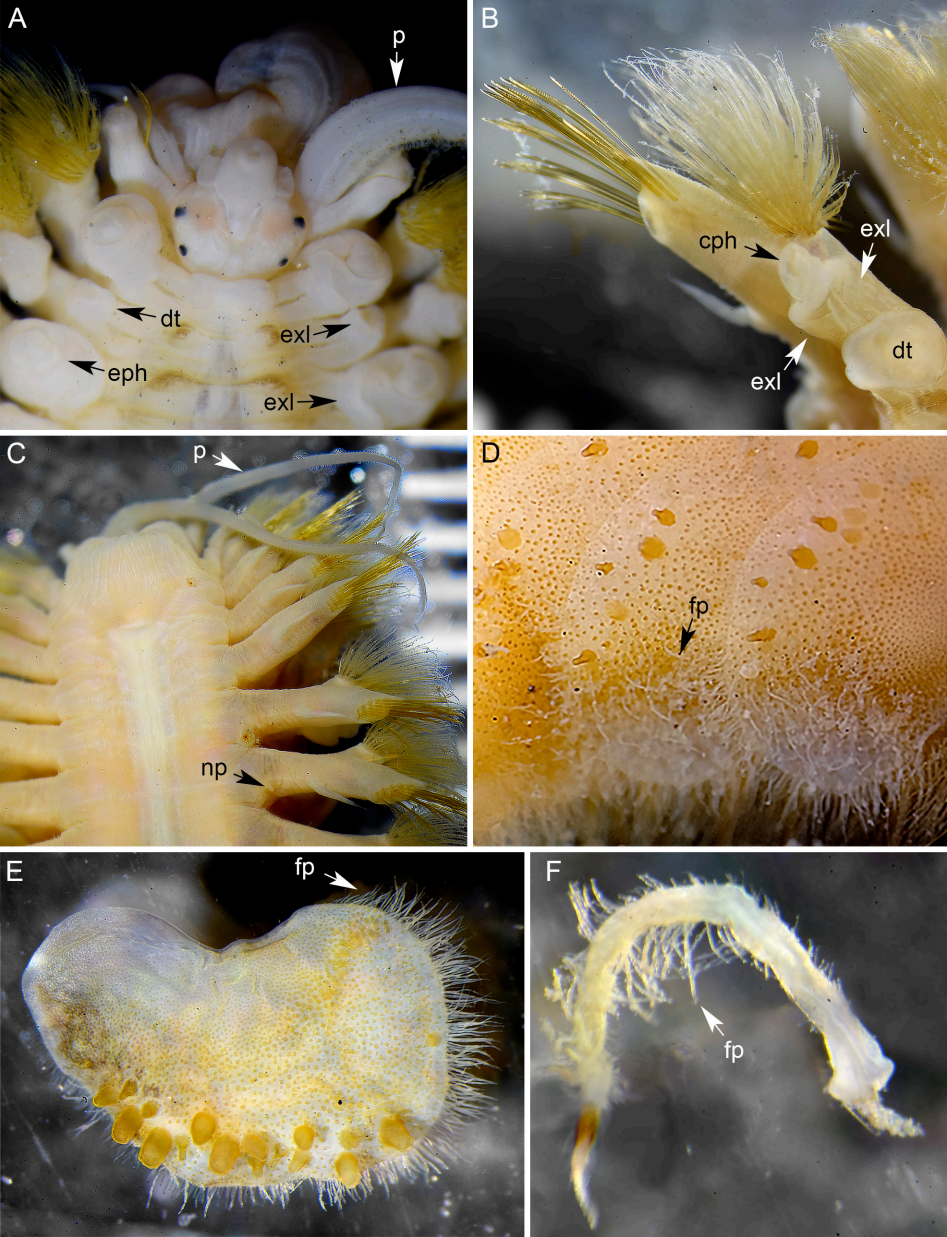
*Eunoe ciliata* comb. nov., light microscopy. USNM 1512583 (**A**), USNM 1077218 (**B, C, E, F**), USNM 32241 (**D**). **A**. Prostomium, dorsal view; **B**. Parapodium with capillary notochaetae, cirrophore with extra lobes, and dorsal tubercle; **C**. Anterior part, nephridial papillae, ventral view; **D**. Elytra with macrotubercles and filiform papillae on surface, dorsal view; **E**. Elytron with macrotubercles with nodular papillae and filiform papillae, dorsal view; **F**. Dorsal cirrus with filiform papillae. Ruler unit: 1 mm. Abbreviations: cph – cirrophore, dt – dorsal tubercle, eph – elytrophore, exl – extra lobe, fp – filiform papillae, np – nephridial papilla, p – palp.

**Figure 12.**
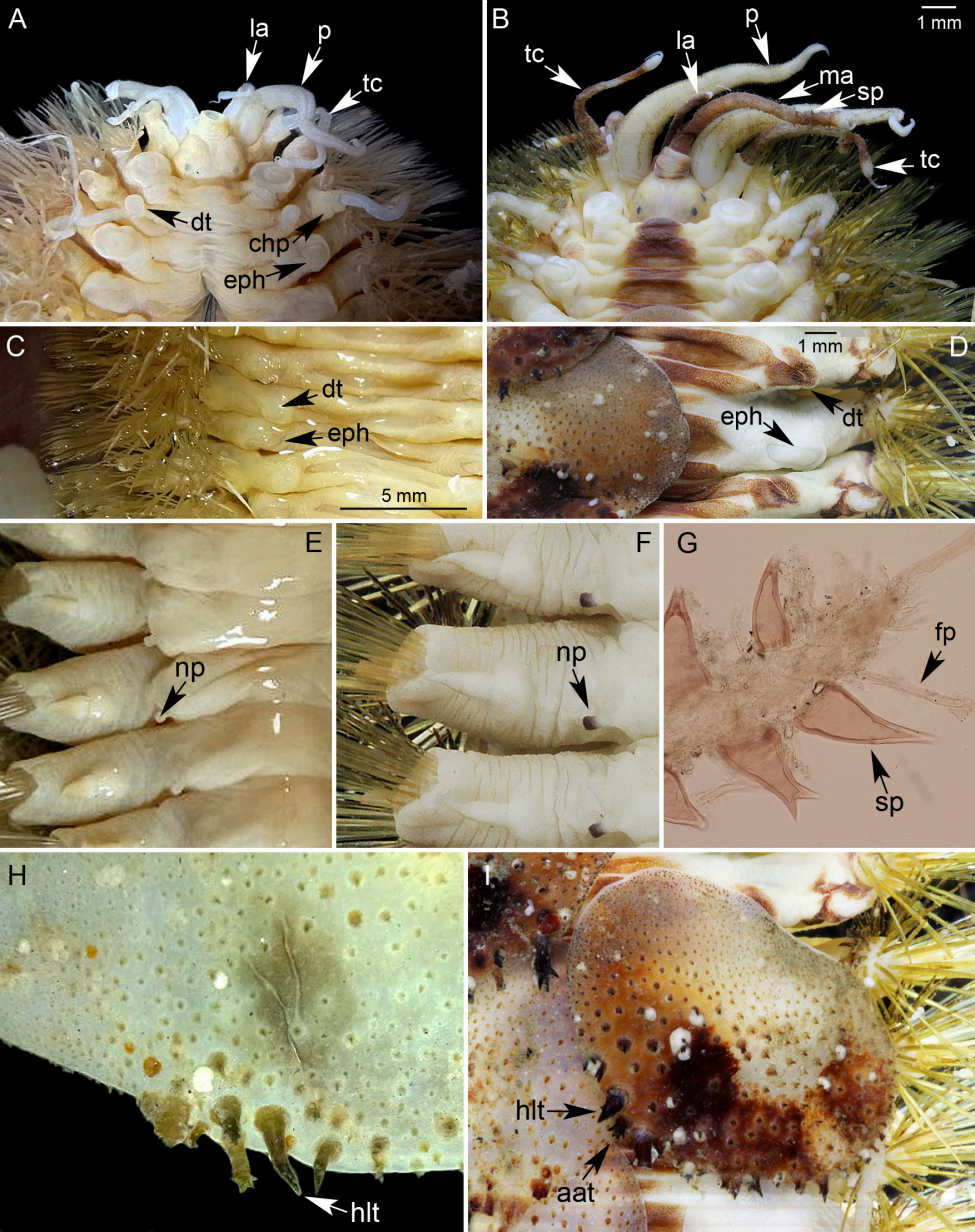
*Eunoe oerstedi*, light microscopy. Lectotype SMNH 2391 (**A, C, E, H**), ZMBN 173048 (**B, D, F, I**), paralectotype SMNH 2395 (**G**). **A, B**. Prostomium, dorsal view; **C, D**. Dorsal tubercles and elytrophores, dorsal view; **E**. Nephridial papillae, ventral view; **F**. Pigmented nephridial papillae, ventral view; **G**. Spines on dorsal cirrus; **H, I**. Elytron with horn-like and apically arborescent macrotubercles, dorsal view. Abbreviations: aat – apically arborescent tubercle, cph – cirrophore, dt – dorsal tubercle, eph – elytrophore, fp – filiform papillae, hlt – horn-like tubercle, la – lateral antenna, ma – median antenna, np – nephridial papilla, p – palp, sp – spine, tc – tentacular cirrus.

**Figure 13.**
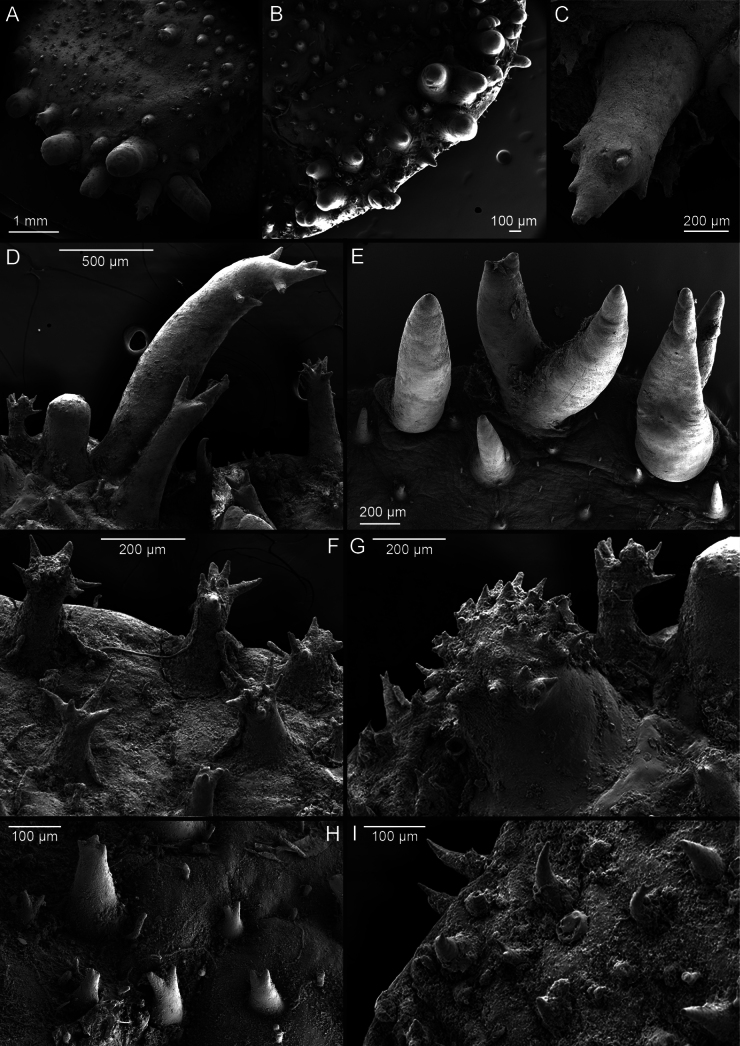
*Eunoe oerstedi*, SEM of elytra from mid-body. Paralectotype SMNH 2389 (large specimen) (**A, C**), NTNU-VM 78191 (large specimen) (**B, E, H**), lectotype SMNH 2391 (large specimen) (**D, F, G, I**). **A, B**. Part of elytron with cylindrical and horn-like macrotubercles, dorsal view; **C–E**. Horn-like macrotubercles; **F, G**. Apically arborescent macrotubercles with conical and pointed branches without secondary branching; **H**. Bifid spines; **I**. Spines.

**Figure 14.**
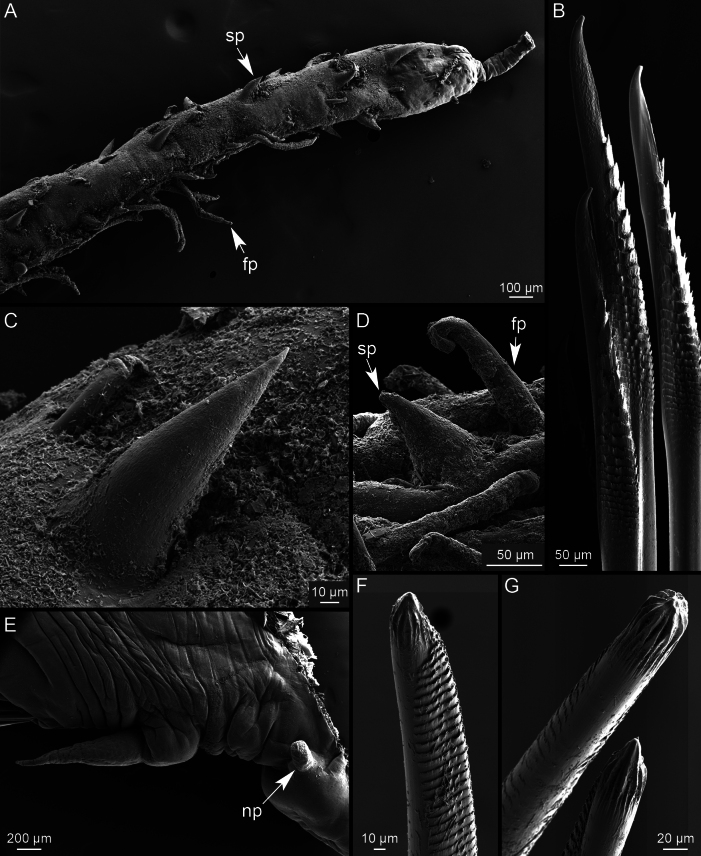
*Eunoe oerstedi*, SEM of parapodia from mid-body. NTNU-VM 78191 (large specimen) (**A–C, E–G**), paralectotype SMNH 2389 (large specimen) (**D**). **A**. Dorsal cirrus with spines and filiform papillae; **B**. Neurochaetae; **C**. Spine on dorsal cirrus; **D**. Spine and filiform papillae; **E**. Parapodium with ventral cirrus and nephridial papilla, ventral view; **F**. Notochaeta with subacute tip; **G**. Notochaetae with blunt and subacute tips. Abbreviations: fp – filiform papillae, np – nephridial papilla, sp – spines.

**Figure 15.**
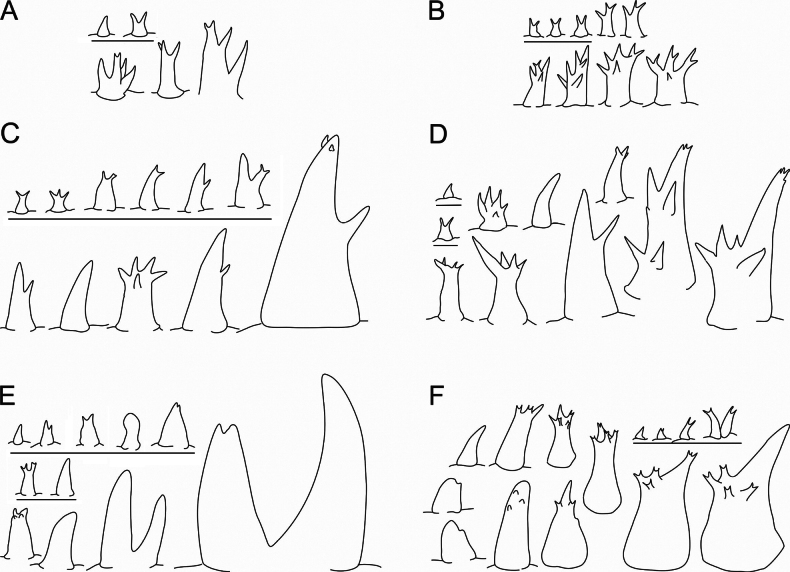
Variation in elytral micro- and macrotubercles in *Eunoe oerstedi*. In each panel, microtubercles are indicated by black horizontal bars. Specimens are grouped by body length: <5 mm (**A, B**), 5–10 mm (**C, D**), and >10 mm (**E, F**): **A**. ZMBN 150359, spines, bifid spines, and macrotubercles with pointed and conical branches with single prominent pointed branch, without secondary branching; **B**. ZMBN 150360, spines, bifid spines, and apically arborescent macrotubercles with pointed and conical branches, without secondary branching; **C**. ZMBN 159268, bifid spines and horn-like macrotubercles with and without branches; **D**. ZMBN 159262, spines, bifid spines, and horn-like macrotubercles with and without branches; **E**. NTNU-VM 78191, spines, bifid spines, and horn-like macrotubercles without branches; **F**. ZMBN 161425, spines, horn-like macrotubercles, and macrotubercles with pointed and conical branches with single prominent pointed branch. Scale: largest macrotubercle 1.5 mm (**E**).

**Figure 16.**
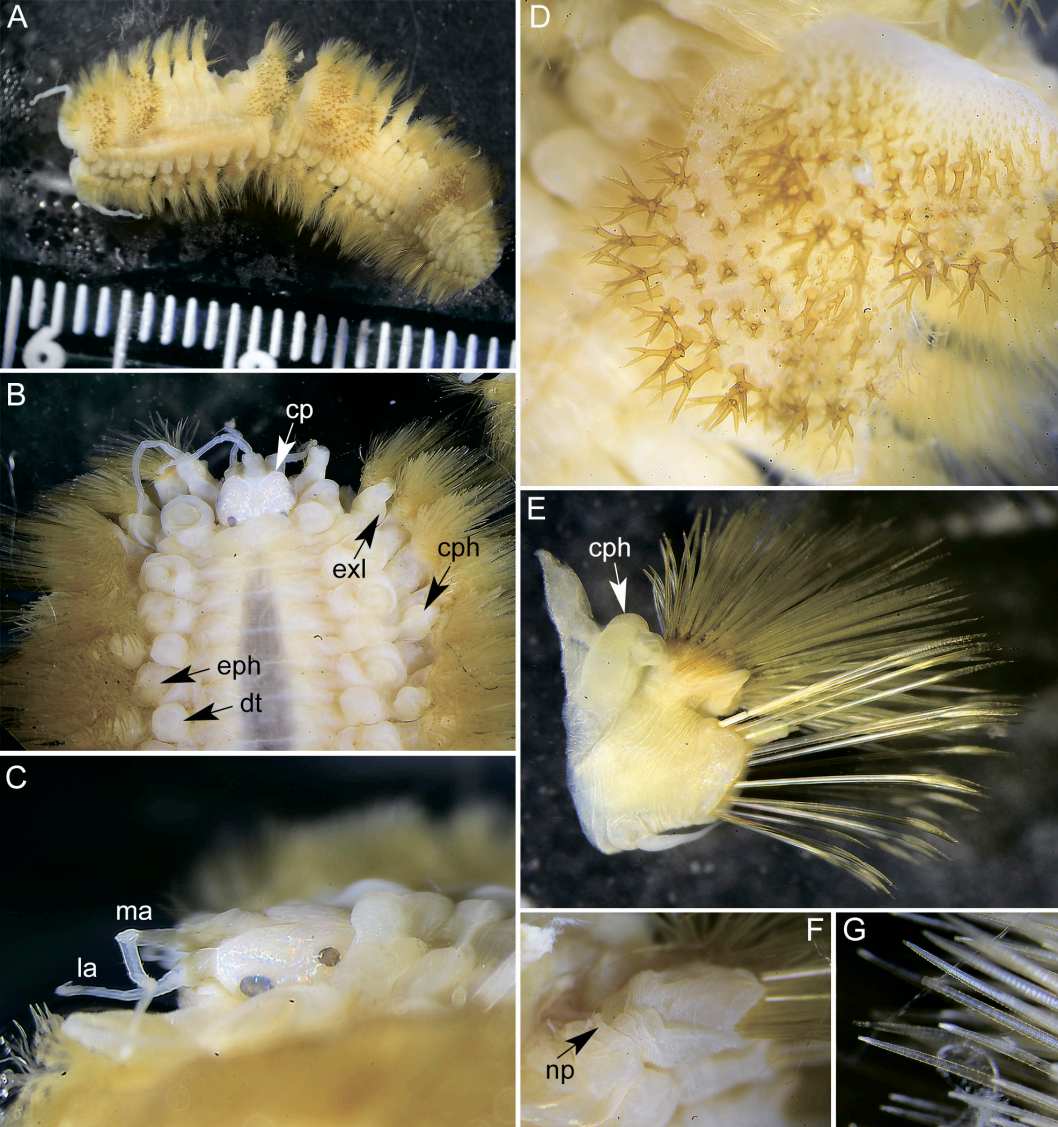
Eunoe sensu lato senta, light microscopy. USNM 5600 (Bowen Island, BC) (**A, D**), USNM 5602 (Alaska) (**B, C, F, G**), USNM 5603 (Alaska) (**E**). **A**. Body with elytra, dorsal view; **B**. Prostomium, cirrophore with extra lobes, elytrophores, and dorsal tubercles, dorsal view; **C**. Prostomium with lateroventral placement of anterior pair of eyes, lateral view; **D**. Elytra with antler-like macrotubercles with distally pointed branchlets, dorsal view; **E**. Parapodium with ventral cirrus and extra lobes on cirrophore; **F**. Nephridial papilla; **G**. Notochaetae. Ruler unit: 1 mm. Abbreviations: cph – cirrophore, dt – dorsal tubercle, eph – elytrophore, exl – extra lobe, la – lateral antenna, ma – median antenna, np – nephridial papilla.

**Figure 17.**
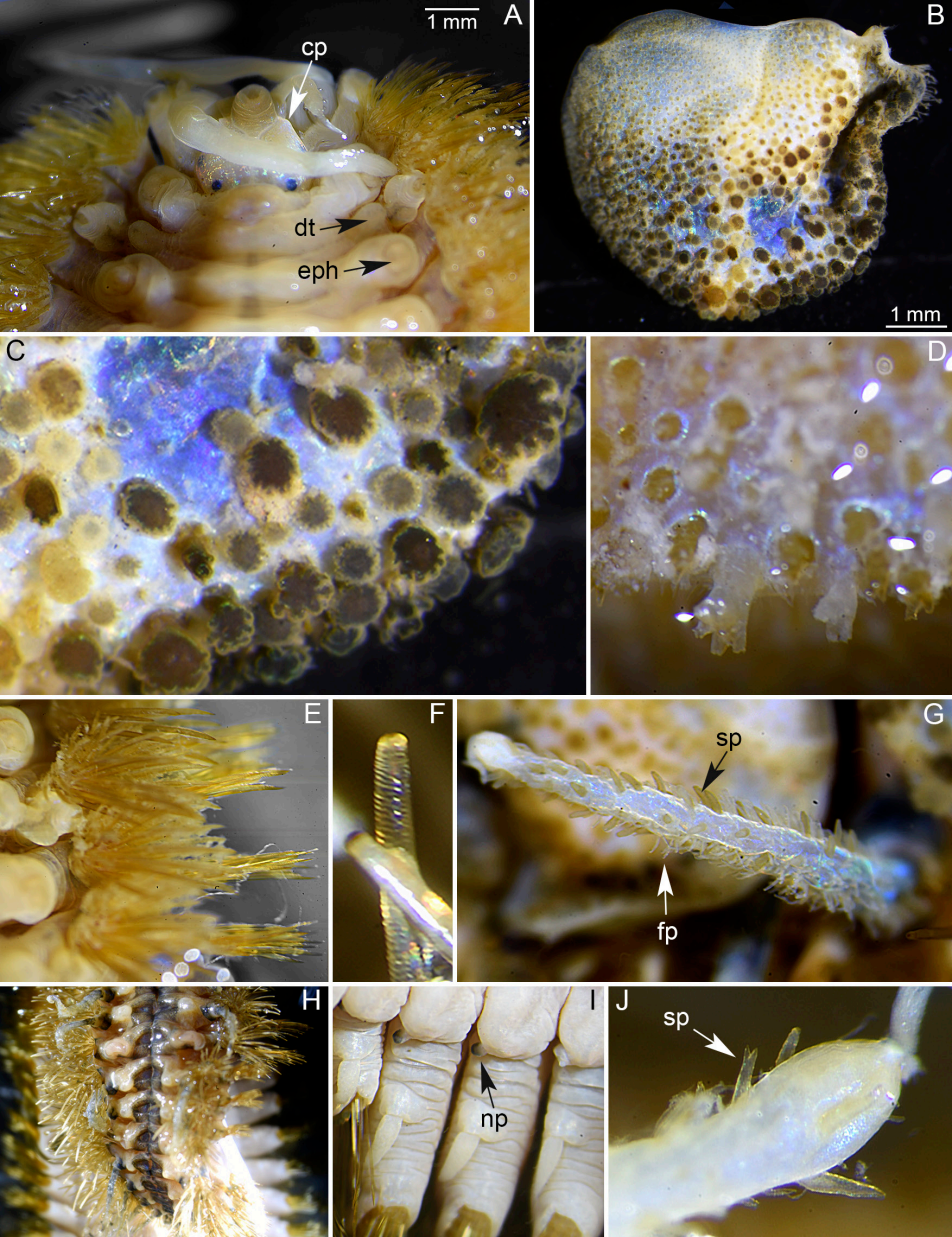
*Eunoe cf. oerstedi* CMC01, light microscopy. USNM 31410 (Alaska) (**A, D, E, G, J**), USNM 30014 (Alaska) (**B, C, F, H, I**). **A**. Prostomium, dorsal tubercles, and elytrophores, dorsal view; **B**. Elytron, dorsal view; **C**. Elytron with macrotubercles with flattened, crown-like tip with undulate margin, dorsal view; **D**. Apically arborescent macrotubercles; **E**. Parapodia, dorsal view; **F**. Notochaetae; **G**. Dorsal cirrus with spines; **H**. Posterior end with color pattern, dorsal view; **I**. Pigmented nephridial papillae, ventral view; **J**. Spines on dorsal cirrus. Abbreviations: cp – cephalic peaks, dt – dorsal tubercle, eph – elytrophore, fp – filiform papillae, np – nephridial papilla, sp – spines.

**Figure 18.**
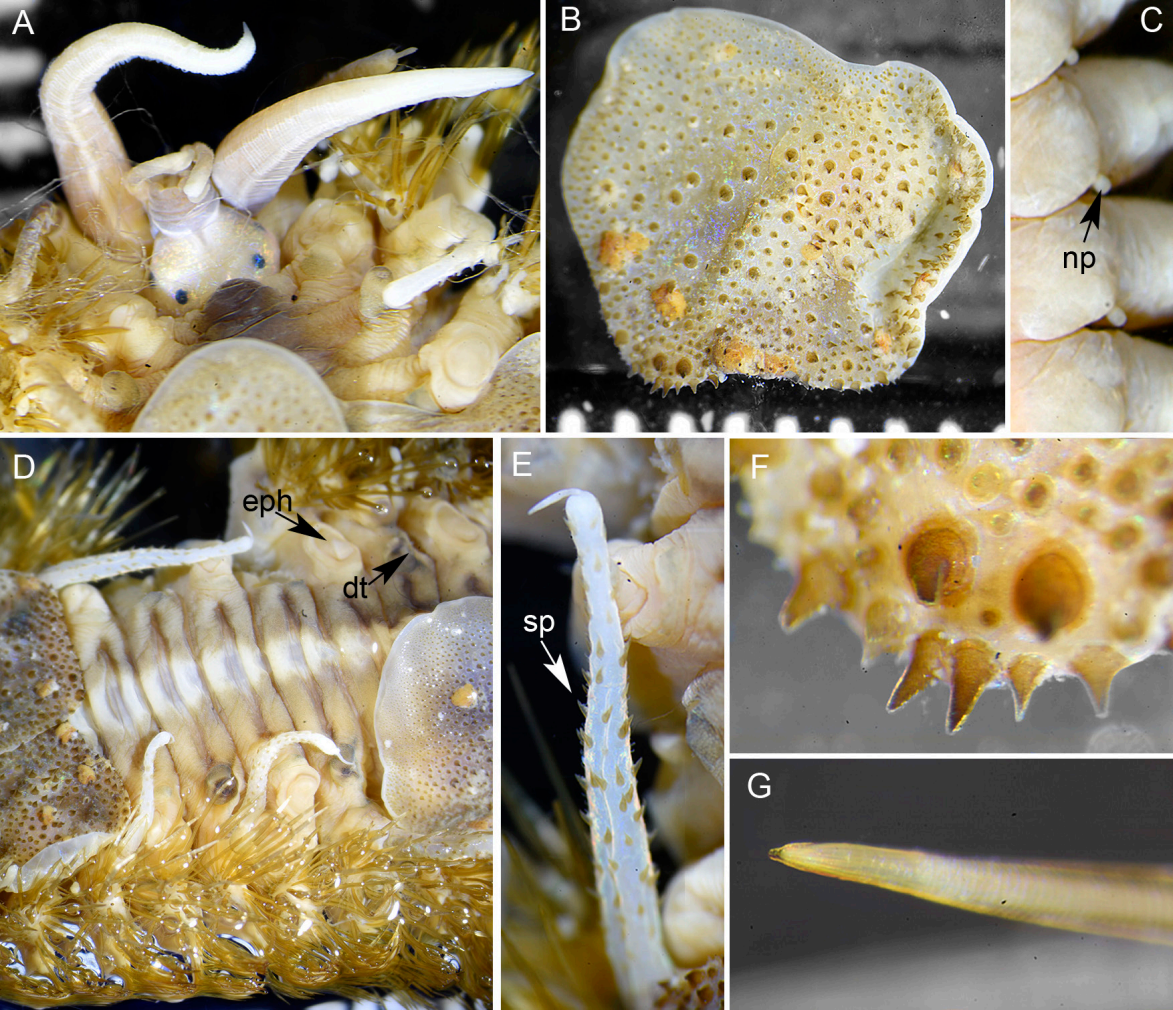
*Eunoe spinicirris*USNM 43578 (Sea of Okhotsk), light microscopy; **A**. Prostomium, dorsal view; **B**. Elytron with spines, dorsal view; **C**. Nephridial papillae, ventral view; **D**. Body with color pattern, elytrophores, and dorsal tubercles, dorsal view; **E**. Dorsal cirrus with large spines; **F**. Elytron with spines, dorsal view; **G**. Notochaeta. Ruler unit: 1 mm. Abbreviations: dt – dorsal tubercle, eph – elytrophore, np – nephridial papilla, sp – spines.

**Figure 19.**
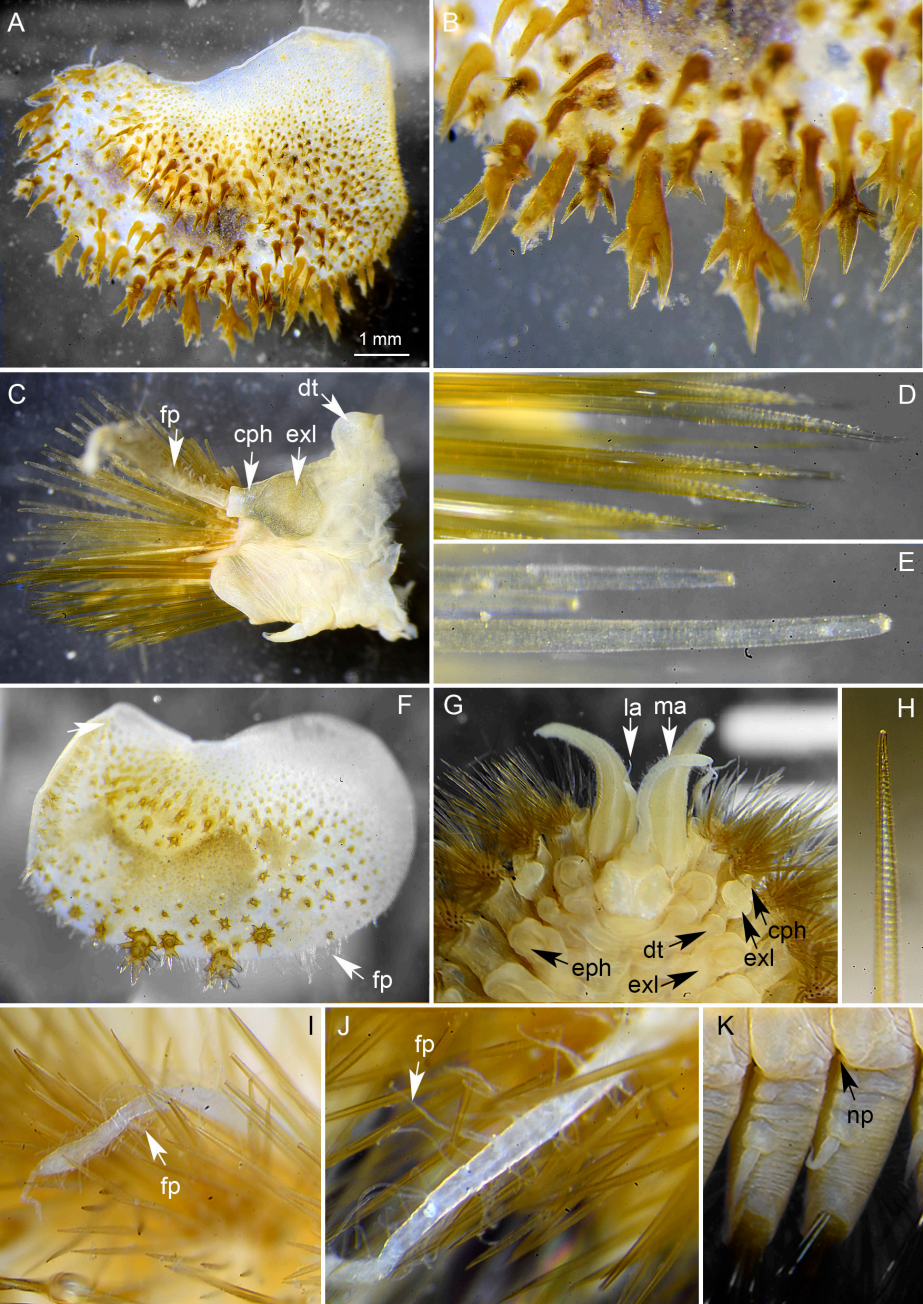
*Eunoe shirikishinai*, holotype USNM 74051 (**A–E**); *Eunoe spinosa*, USNM 23762 (**F–K**), light microscopy. **A**. Elytron, dorsal view; **B**. Macrotubercles, dorsal view; **C**. Parapodium with dorsal and ventral cirri, lateral view; **D**. Neurochaetae; **E**. Notochaetae; **F**. Elytron, dorsal view; **G**. Prostomium, dorsal tubercles, and elytrophores with extra lobes, dorsal view; **H**. Notochaeta; **I, J**. Dorsal cirrus with long filiform papillae; **K**. Nephridial papillae, ventral view. Abbreviations: cph – cirrophore, dt – dorsal tubercle, eph – elytrophore, exl – extra lobe, fp – filiform papillae, la – lateral antenna, ma – median antenna, np – nephridial papilla.

**Figure 20.**
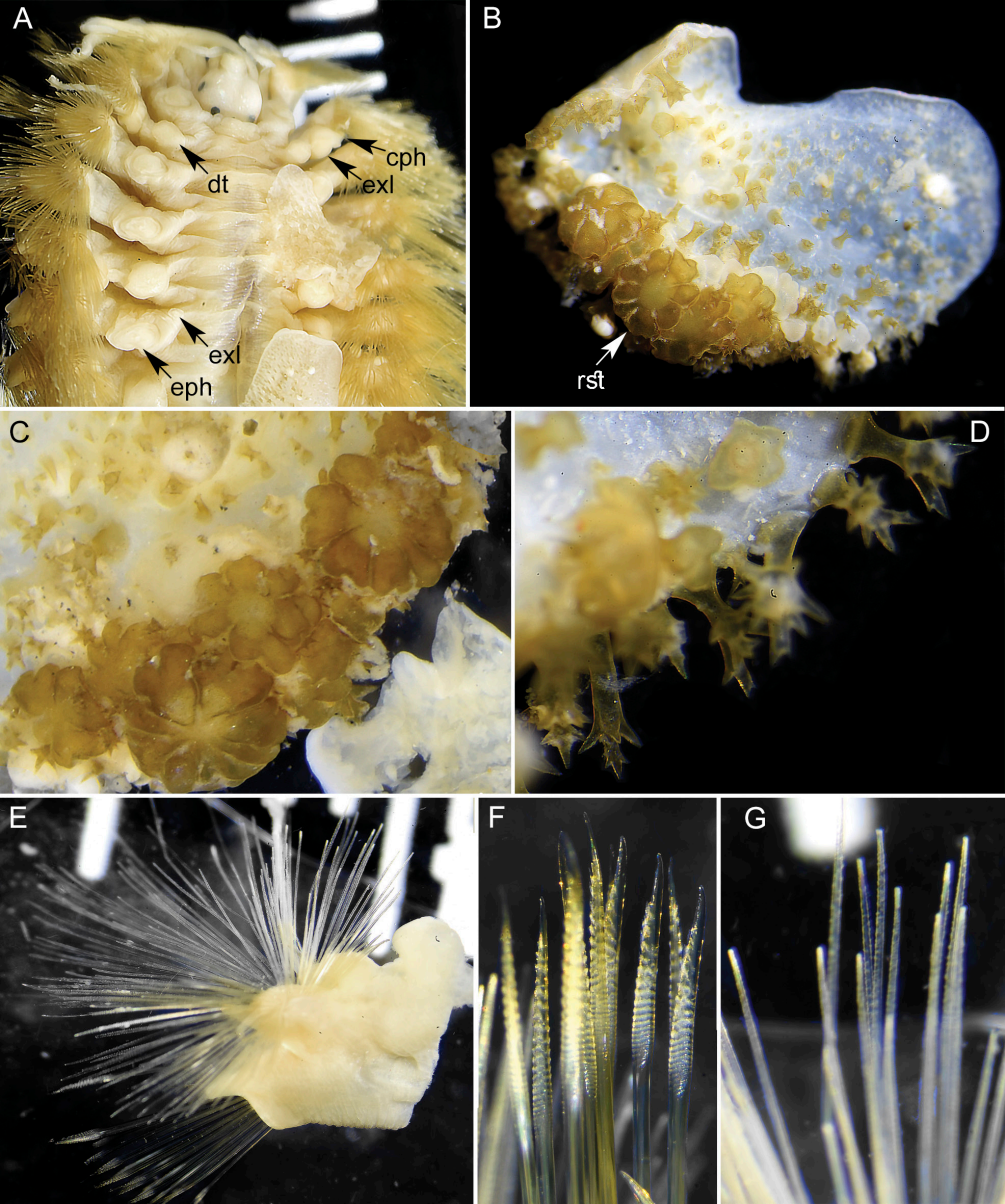
*Eunoe sentiformis*USNM 43582 (Kuril Islands), light microscopy. **A**. Prostomium, dorsal tubercles, elytrophores with extra lobes, and cirrophores with extra lobes, dorsal view; **B**. Elytron with cylindrical macrotubercles, ending distally in rosette-shaped apex with strongly lobate margin (“flower-like” head with five to eight petals), dorsal view; **C**. Rosette-shaped apex of macrotubercles, dorsal view; **D**. Cylindrical macrotubercles with dentate crown at tip; **E**. Parapodium; **F**. Neurochaetae; **G**. Notochaetae with truncate tips. Ruler unit: 1 mm. Abbreviations: cph – cirrophore, dt – dorsal tubercle, eph – elytrophore, exl – extra lobe, rst – rosette-shaped tubercle.

**Figure 21.**
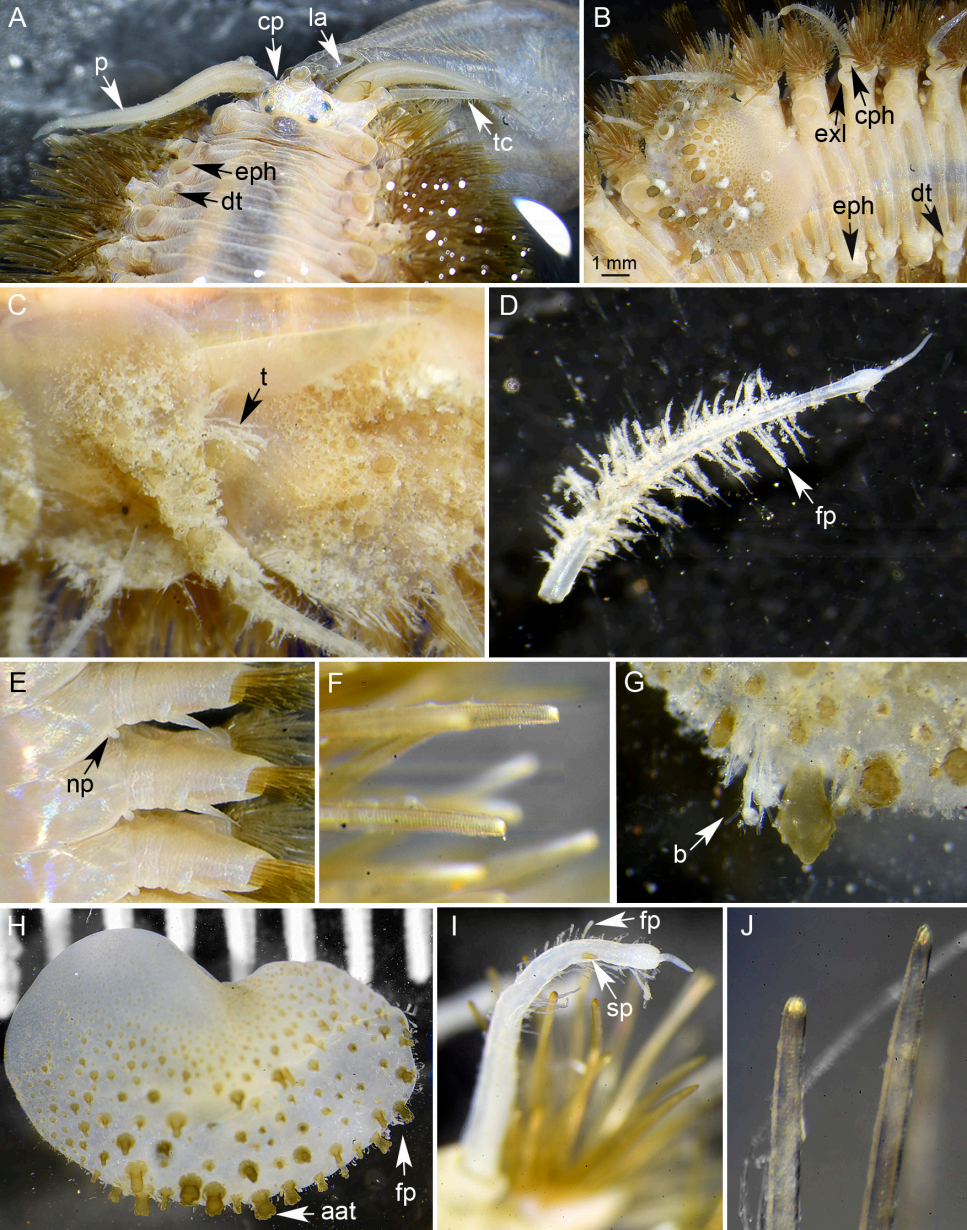
*Eunoe barbata*USNM 25205 (Puget Sound, WA) (**A, C, E)**, USNM 25203 (Puget Sound, WA) (**B, F**), USNM 25199 (San Juan Islands, WA) (**D**), USNM 25191 (Whidbey Island, WA) (**G**) and *E. cf. oerstedi*USNM 43580 (Sea of Okhotsk) (**H–J**), light microscopy. **A**. Prostomium, elytrophores, and dorsal tubercles, dorsal view; **B**. Mid-body region with elytron, extra lobes on cirrophores, dorsal view; **C**. Papillation of elytra with tuft of filiform papillae; **D**. Dorsal cirrus; **E**. Nephridial papillae, ventral view; **F**. Notochaetae with truncate tips; **G**. Macrotubercles and tuft of filiform papillae; **H**. Elytron, dorsal view; **I**. Dorsal cirrus with spines; **J**. Notochaetae with blunt rounded tips. Ruler unit: 1 mm. Abbreviations: aat – apically arborescent tubercle, cp – cephalic peaks, cph – cirrophore, dt – dorsal tubercle, eph – elytrophore, exl – extra lobe, fp – filiform papillae, la – lateral antenna, np – nephridial papilla, p – palp, sp – spine, t – tuft of filiform papillae, tc – tentacular cirrus.

Specimens were examined using a dissecting stereomicroscope. Permanent slides of parapodia were mounted in Hydro-matrix® and examined under a compound light microscope. Specimens were photographed using a Canon EOS 60D single reflex camera from the dorsal and ventral sides, showing the whole specimen, and also focusing on characters important for identification, such as the shape of the prostomium, elytra, and cirri. Measurements were taken from photographs: the specimen’s length and the widest segment’s width (excluding chaetae). For light microscopy and SEM imaging, parapodia were dissected from the mid-body region (approximately the middle third of the body). Six specimens were used for scanning electron microscopy (SEM). The samples were dehydrated in ethanol (EtOH) and Hexamethyldisilazane (HMDS) according to the protocol (3:1 EtOH:HMDS 1 h, 1:1 EtOH:HMDS 1 h, 1:3 EtOH:HMDS 1 h, HMDS 1 h), air dried in a fume hood, mounted on stubs, gold/palladium coated, and photographed with a Zeiss Supra 55VP scanning electron microscope at the Electron Microscopy Lab of the University of Bergen (ELMILAB). Morphology of the species discussed in this study is shown in Figs 4, 5, 6, 7, 8, 9, 10, 11, 12, 13, 14, 15, 16, 17, 18, 19, 20, 21.

The terminology generally follows Pettibone (1953, 1963) and Barnich and Fiege (2010), with minor changes in the description of macrotubercles. Specimens were grouped by body size into small (<5 mm long), medium (5–10 mm), and large (>10 mm), allowing assessment of intraspecific morphological variation. Below, we summarize the terminology used to describe different morphological structures in *Eunoe* by various authors and suggest several new characters used in species diagnoses.

In polynoids, the elytrigerous segments bear elytra on elytrophores, which may possess or lack an extra lobe, and cirrigerous segments bear dorsal cirri and dorsal tubercles (Darboux 1900), situated dorsally near the cirrophore, and may also possess or lack an extra lobe. In *Eunoe*, elytra are present on segments: 2, 4, 5, 7, 9, 11, 13, 15, 17, 19, 21, 23, 26, 29, and 32.

Many polynoid genera, including *Eunoe* and *Gattyana* bear four eyes arranged in two pairs on the prostomium. The posterior pair lies dorsally near the posterior margin. The anterior pair may occupy one of three positions: dorsally on the widest part of the prostomium (Fig. 11A), dorsolaterally (Fig. 12B), or ventrally beneath the anterior margin or cephalic peaks, in which case the anterior eyes are not visible in dorsal view (Fig. 16A, C).

The nephridial papilla (Darboux 1900) is located ventrally at the base of the parapodium, starting from the 4^th^ or 5^th^ segment in *Eunoe*. It may be covered by a nephridial sac and only visible if the sac is moved aside (Figs 5C, 7E, 16F). Alternatively, the nephridial papilla may be uncovered and visible from the ventral side of a specimen, and can also be pigmented (Figs 11C, 12E, 12F, 14E, 17I, 18C, 19K, 21E).

The shape of macrotubercles is one of the most important and prominent characters in *Eunoe* identification, often used to distinguish the two most common Arctic species, *Eunoe nodosa* and *E. oerstedi* (Barnich 2011). Macrotubercles of *E. nodosa* were usually described as “nodular, with roughened tips or a fascicle of short spikes” (Pettibone 1963: 44) or “conical to cylindrical, distally with nodular papillae” or “distally with spiny papillae” (Barnich and Fiege 2010: 6). Macrotubercles of *E. oerstedi* were described as “branched, extremely variable in size, number, arrangement, and shape” (Pettibone 1963: 44) or as “cylindrical and distally branched multifid, or spiny” (Barnich and Fiege 2010: 8). These descriptions are somewhat misleading because both species may bear branched macrotubercles. However, they can be distinguished by the branching architecture: apically crown-bearing, “apically arborescent”, and tapering, “horn-like”, macrotubercles.

The “apically arborescent” macrotubercles (Fig. 4A) consist of a conical to cylindrical trunk that does not markedly taper distally, sometimes with a slight basal swelling, and a compact apical crown of short branchlets. Branching is confined to the distal tip, the branchlets are usually short and subequal. In *E. nodosa*, the branchlets often form a dense dichotomously divided apical crown, producing a “baobab-like” silhouette (Figs 4A, 5F, 5G, 6A, 6C–E, 8A–F). In *E. nodosa*, the apically arborescent macrotubercles frequently co-occur with unbranched ones: these macrotubercles are semiglobose (dome-shaped) to short cylindrical, with a distally rounded, roughened, nodulose apex and no discrete branchlets.

In *E. oerstedi*, macrotubercles are likewise polymorphic and include an apically arborescent, crown-bearing morphotype, but their distal processes are typically irregular and non-dichotomous, in contrast to the often dichotomously divided apical crown in *E. nodosa*. In *E. oerstedi*, the second morphotype is the “horn-like” macrotubercle (Fig. 4C): tall, tapering conical macrotubercles with a dominant main axis, either unbranched or bearing one (rarely a few) lateral outgrowth(s), usually arising along the trunk or near its distal part rather than forming a symmetric apical crown. The “horn-like” macrotubercles are size-dependent, shifting from shorter cones with more distal processes in small specimens to longer cones with fewer processes in larger specimens (Fig. 15). In the largest specimens, they may be completely unbranched and resemble a “carrot-like” cone (Figs 12H, 13E, 15E).

Other species recovered in the clade containing *Eunoe nodosa* show additional macrotubercle morphologies. *Eunoe ciliata* comb. nov. bears two types of unbranched macrotubercles: stout, bluntly conical macrotubercles with a nodulose, rough tip, and more slender conical, blunt-tipped macrotubercles. In *E. cf. oerstedi* CMC01, macrotubercles are cylindrical and end distally in a flattened, crown-like tip with an undulate margin, often appearing as coronate discs in top view. In *E. spinicirris*, macrotubercles are simple conical spines with occasionally bifid tips. In *E. shirikishinai*, macrotubercles are spiniform, often furcate and variably branched. In *E. barbata*, macrotubercles are cylindrical to slightly clavate with an apically coronate, crenulate apex. In *E. sentiformis*, two types of macrotubercles are present: cylindrical macrotubercles, ending distally in a rosette-shaped apex with a strongly lobate margin (a “flower-like” head) and cylindrical macrotubercles with a dentate crown at the tip. Macrotubercles of other species currently assigned to *Eunoe* can be described as very low, sessile mammilliform (E. s. l. depressa) or as antler-like, long-branched arborescent with distally pointed branchlets (E. s. l. senta) (Fig. 4).

Dorsal and tentacular cirri and antennae of *Eunoe* species are often covered with long, filiform papillae. However, in the present study, we also observed spines, wide at the base and sharply tapering, resembling rose thorns (Figs 12G, 14A, 14C, 14D). Spines on different body parts have been previously documented in two *Eunoe* species. In particular, Annenkova (1937) described *Eunoe spinicirris* as having spines on the antennae, dorsal and tentacular cirri, and elytra. Uschakov (1982) mentioned similar spines on the antennae and on dorsal and tentacular cirri of *E. oerstedi*, although he did not consider this character diagnostic. Similar spines (microtubercles) were also reported on the elytra of some but not all species (Figs 6F, 13I, 18F, 19A).

For chaetal terminology, notochaetae and neurochaetae are described following Barnich and Fiege (2010). However, to avoid confusion between spines on body appendages or elytra and those occurring on chaetae, we refer to the latter as spinules.

## Results

### Museomics

Short-read sequencing of the historical type specimen of *Eunoe oerstedi* recovered 6,049,029 raw reads. The DNA appeared fragmented with an average read length of 19.2 bp (median 17 bp). After quality filtering and adapter trimming, 4,656,465 reads were available for phylogenetic placement of the specimen.

De novo assembly with SPAdes yielded 1,186 contigs ranging from 22 to 2,435 bp in length (N50 = 211). BLAST searches against known polynoid sequences identified three separate 18S rRNA fragments (310, 762, and 368 bp, with mean coverages ranging from 20.5× to 21.3×) and the nearly complete ITS2–28S rRNA region (3,038 bp, mean coverage 21.7×), assembled from two overlapping contigs (644 bp and 2,435 bp).

For the mitochondrial loci, reads were successfully assembled in MIRA and mapped in Mitobim to recover six fragments of COI (12 reads) with lengths of 82, 33, 34, 31, 38, and 64 bp (42.9% complete with regard to the reference sequence ZMBN 159262; coverage = 1× for all fragments except the last one, with coverage 3.2×), and two fragments of 16S (18 reads) with lengths of 143 bp and 103 bp (50.2% complete; coverage = 3.5× and 1.9×, respectively).

The resulting consensus sequences for all markers (nuclear: ITS2, 28S rRNA; mitochondrial: COI, 16S rRNA) were used for subsequent phylogenetic analyses.

### Phylogenetic analysis

The final concatenated alignment included 217 specimens and 2689 aligned positions and comprised four loci: COI 657 bp, 16S rRNA 513 bp, ITS2 379 bp, and 28S rRNA 1140 bp. The alignment contained 1003 variable sites, of which 672 were parsimony-informative. Sequence data were available for 210 specimens for COI, 105 for 16S rRNA, 33 for ITS2, and 98 for 28S rRNA. Missing data, including Ns and gaps, accounted for 53.11% of the concatenated matrix, with 296,237 Ns and 13,648 gaps, and locus-specific missingness of 7.44% for COI, 54.77% for 16S rRNA, 86.61% for ITS2, and 67.54% for 28S rRNA.

Maximum-likelihood analysis (ML) and Bayesian inference (BI) of the combined dataset recovered largely congruent topologies (Suppl. material 2: figs S1, S2). The main differences concerned three relationships within the ingroup recovered only in the ML analysis with low nodal support (nodes labelled 44/–, 67/–, and 68/–; Fig. 1), whereas all species-level clades were supported by both approaches. Hereafter, ML nodal support is reported as UFBoot. The SH-aLRT values are shown on the ML tree in the Suppl. material 2: fig. S1.

The genus *Eunoe* was recovered as polyphyletic. A well-supported clade (UFBoot = 100; BPP = 1) including the type species, *Eunoe nodosa* (UFBoot = 100; BPP = 0.99), is further referred to as the *Eunoe* sensu stricto clade. Other species in the *Eunoe* s. str. clade were *E. shirikishinai* (UFBoot = 100; BPP = 1), *E. oerstedi* (UFBoot = 96; BPP = 0.71), *E. spinicirris* (BENTH238-08), *E. cf. oerstedi* CMC01 (UFBoot = 99; BPP = 0.99), and *Gattyana ciliata*, which is further referred to as *Eunoe ciliata* comb. nov. (UFBoot = 100; BPP = 1). The lectotype SMNH 2391 of *Eunoe oerstedi* nested within the *E. oerstedi* clade (Fig. 1; Suppl. material 2: figs S1, S2).

All the remaining species of *Gattyana* formed a single clade, here treated as *Gattyana* sensu stricto (UFBoot = 99; BPP = 1), containing the type species—*Gattyana cirrhosa* (Pallas, 1766) (SMNH 118965), *Gattyana cf. cirrhosa* (UFBoot = 100; BPP = 0.99), *G. amondseni* (Malmgren, 1867) (UFBoot = 97; BPP = 0.90), and *Gattyana cf. amondseni* (UFBoot = 100; BPP = 0.91). *Gattyana* s. str. was recovered as the sister taxon to *Eunoe* s. str., but support for this node was low (UFBoot = 88; BPP = 0.55).

For convenience, species currently assigned to *Eunoe* but excluded from *Eunoe* s. str. are further referred to as *Eunoe* sensu lato. This designation is purely operational and does not imply monophyly or formal recognition of a residual genus. The remaining species of *Eunoe* s. l. included in the analysis were recovered as fifteen independent clades distributed across the tree (Fig. 1A–D). Eleven of the *Eunoe* s. l. species were recovered as well-supported monophyletic groups (UFBoot > 95; BPP > 0.95): Eunoe s. l. depressa, E. s. l. etheridgei (Benham, 1915), E. s. l. issunboushi, E. s. l. apicolata, E. s. l. brunnea Hartman, 1978, E. s. l. bathydomus (Ditlevsen, 1917), E. s. l. benhami, E. s. l. danmurrayi, E. s. l. leiotentaculata Averincev, 1978, E. s. l. albacauda, and *Eunoe* s. l. sp. 1. Eunoe s. l. opalina McIntosh, 1885 was split into two clades; and two provisional species, *Eunoe* s. l. sp. K40 GBMND29788-21 and *Eunoe* s. l. sp. Pen23 GBMND29789-21, were singletons.

Several of *Eunoe* s. l. species formed highly supported clades (A–D; Fig. 1). Clade A (UFBoot = 95; BPP = 0.93) comprised Eunoe s. l. depressa, and two presumably cryptic species *Harmothoe cf. rarispina* (M. Sars, 1861) CMC01 and CMC03. Within this clade, E. s. l. depressa was a sister taxon to *H. cf. rarispina* CMC01 (UFBoot = 99; BPP = 1). Clade B (UFBoot = 100; BPP = 1) included Eunoe s. l. etheridgei and E. s. l. issunboushi. Clade C (UFBoot = 100; BPP = 1) comprised eight well-supported groups (UFBoot > 95; BPP > 0.95): Eunoe s. l. bathydomus, E. s. l. benhami, E. s. l. danmurrayi, E. s. l. opalina, *Eunoe* s. l. sp. 1, *Harmothoe paxtoni* Averincev, 1978, *H. longipalpa* (Kirkegaard, 1995), and *H. torbeni* (Kirkegaard, 1995). Clade D (UFBoot = 100; BPP = 1) included Eunoe s. l. leiotentaculata and E. s. l. albacauda. Other species of *Eunoe* s. l. were singletons distributed across the tree.

Notably, the specimen labelled *Eunoe nodosa*SMNH 118963 was genetically nearly identical to *Harmothoe cf. fragilis*, indicating that this specimen was misidentified in Norlinder et al. (2012). The sequence MK390764, originally identified as *Eunoe uniseriata*, was nested within the *Eunoe nodosa* clade in our analyses, indicating that this specimen was a misidentified specimen of *E. nodosa*.

### Species delimitation

The best ASAP partition (i.e., with the lowest score) based on COI recovered six groups fully congruent with the six clades resolved within *Eunoe* s. str. in the phylogenetic analyses: *Eunoe nodosa*, *E. ciliata* comb. nov., *E. oerstedi*, *E. cf. oerstedi* CMC01, *E. spinicirris*, and *E. shirikishinai* (Fig. 1). The 16S rRNA dataset recovered three of these species (*E. nodosa*, *E. oerstedi*, and *E. shirikishinai*) (Fig. 1), and the 28S rRNA dataset recovered four (*E. nodosa*, *E. ciliata* comb. nov., *E. oerstedi*, and *E. shirikishinai*). The remaining COI-defined groups were not represented in these datasets. ITS2 was available only for *E. nodosa* and *E. oerstedi*: *E. nodosa* was recovered as a distinct group, whereas *E. oerstedi* was split into two ASAP groups (Suppl. material 2: file S1C).

### Haplotype network analysis

The COI haplotype network reconstruction of the clade *Eunoe* s. str. included 90 sequences from six geographical regions (Fig. 2). The network revealed six distinct haplotype groups corresponding to six species: *E. nodosa*, *E. ciliata* comb. nov., *E. oerstedi*, *E. spinicirris*, *E. shirikishinai*, and *E. cf. oerstedi* CMC01. *Eunoe nodosa* consisted of two haplogroups, one from the Eastern Atlantic region and the North Polar region, and the other from the North Polar region and the Bering Strait, representing the boundary between the North Polar and the Pacific regions. Two haplogroups were also recognized in *E. ciliata* comb. nov., one from the Eastern Pacific region and the other from the Bering Strait. *Eunoe oerstedi* formed a single haplogroup with a central haplotype reported from both the North Polar and Western Atlantic regions.

The haplotype networks for the other three genes (16S rRNA, ITS2, and 28S rRNA) included fewer taxa due to the lack of sequence data available in GenBank and BOLD (Fig. 3). However, the two target species, *E. nodosa* and *E. oerstedi*, analyzed in detail in the present study, were represented by distinct haplotype groups across both mitochondrial and nuclear markers. In both the 28S rRNA and ITS2 haplotype networks, the lectotype SMNH 2391 of *E. oerstedi* was placed within the haplotype group of *E. oerstedi*. For the mitochondrial loci, only short fragmentary sequences were recovered from the lectotype, precluding their inclusion in the haplotype network analyses. The 16S rRNA and 28S rRNA haplotype networks included haplotypes assigned to *E. shirikishinai*. The 28S rRNA network also included a single haplotype of *E. ciliata* comb. nov., separated by two substitutions from *E. oerstedi* and by three substitutions from *E. nodosa*.

### Systematic account

#### Polynoidae Kinberg, 1856

##### Eunoe

Taxon classificationAnimaliaPhyllodocidaPolynoidae

Malmgren, 1865

46ACAD8C-8FA2-5281-8C55-F358FCE9E23A

Fig. 4

Eunoe Malmgren, 1865: 61, in part.

###### Type species.

*Polynoe nodosa* M. Sars, 1861 (type subsequently designated by Uschakov, 1955).

###### Type locality.

Havösund, Nordkap.

###### Diagnosis.

Amended from Barnich and Fiege (2010). Body dorsoventrally flattened, short, with ≤ 50 segments; dorsum more or less covered by elytra or short posterior region uncovered. Fifteen pairs of elytra on segments 2, 4, 5, 7, 9, 11, 13, 15, 17, 19, 21, 23, 26, 29, and 32. Elytra with micro- and macrotubercles often branching. Prostomium with or without distinct cephalic peaks and three antennae; lateral antennae inserted ventrally to median antenna. Palps with six longitudinal rows of small papillae. Anterior pair of eyes dorsolateral at widest of prostomium, posterior pair dorsal near hind margin. Parapodia with elongate acicular lobes; both aciculae penetrating epidermis; neuropodia with supra-acicular process. Notochaetae stout with distinct rows of spinules, with acute, subacute, blunt, or truncate tips; notochaetae capillary. Neurochaetae stout, with distinct rows of spinules distally and exclusively unidentate tips.

###### Remarks.

The generic diagnosis was amended from Barnich and Fiege (2010) by adding the palp character, six longitudinal rows of small papillae; adding elytral ornamentation, micro- and macrotubercles, often branching; replacing “rows of spines” with “rows of spinules” for chaetal ornamentation; and broadening the notochaetal character to include stout notochaetae with acute, subacute, blunt, or truncate tips, as well as capillary notochaetae.

##### Eunoe nodosa

Taxon classificationAnimaliaPhyllodocidaPolynoidae

(M. Sars, 1861)

5A38CF05-962C-5A9F-B527-CD0C6A19B6C0

Figs 5, 6, 7, 8, 10A

Polynoë nodosa M. Sars, 1861: 58.
Polynoë scabra Theel, 1879: 7 [part].
Polynoë (Eunoë) islandica Hansen, 1878: 2, T. I fig. 4, T. II, figs 2, 3, 5–7.
Polynoë arctica Hansen, 1878: 267, T. I, figs 1–5.
Polynoë spinnulosa Hansen, 1882, Hansen 1880: 225, T. II, figs 6–10.
Polynoë foramenifera Hansen, 1882, Hansen 1880: 226, T. III, figs 6–11.
Eunoë nodosa .—Malmgren 1865: 64, pl. VIII fig. 4; Pettibone 1954: 217, fig. 26c.
Eunoa nodosa .—Malmgren 1867: 6; McIntosh 1900: 291, pl. 27 fig. 9, pl. 32, fig. 3, pl 37, figs 20, 22, 24, 26, 27, pl. 42, fig. 28.
Harmothoë (Eunoe) nodosa .—Pettibone 1963: 44, fig. 9a–c.
Eunoe nodosa .—Loshamn, 1980: 185, fig. 91A-f;—Uschakov 1982: 176, pl. LXV, figs 6–7;— Jirkov 2001: 147, figs on page 148 (1–4);—Barnich and Fiege 2010: 6, fig. 2A–K.

###### Type material examined.

***Lectotype (here designated)*** • NHMO IN-5334 (1 spm) (former acc. number NHMO C5274). Elytra detached from the lectotype (NHMO IN-5334): NHMO IN-5303 (former acc. number ZMO/NHMO C5243; listed as ZMO C5243 in Barnich and Fiege 2010) and NHMO IN-5335 (former acc. number NHMO C5275). ***Paralectotype*** • NHMO IN-5336 (1 spm) (former acc. number NHMO C5276).

###### Additional type material (not examined).

***Paralectotype*** • NHMO IN-5304 (1 spm) (former acc. number ZMO/NHMO C5244; listed as ZMO C5244 in Barnich and Fiege 2010).

###### Type locality.

Havösund, Nordkap.

###### Other material examined.

• ZMBN 104861 (1 spm), ZMBN 104887 (1 spm), ZMBN 104896 (1 spm), ZMBN 116524 (1 spm), ZMBN 116525 (1 spm), ZMBN 120373 (1 spm), ZMBN 120375 (1 spm), ZMBN 108081 (1 spm), ZMBN 114338 (1 spm), ZMBN 116162 (1 spm), ZMBN 149383 (1 spm), ZMBN 150361 (1 spm), ZMBN 150362 (1 spm), ZMBN 150363 (1 spm), ZMBN 150364 (1 spm), ZMBN 150365 (1 spm), ZMBN 150367 (1 spm), ZMBN 150368 (1 spm), ZMBN 150369 (1 spm), ZMBN 150370 (1 spm), ZMBN 150371 (1 spm), ZMBN 150372 (1 spm), ZMBN 150373 (1 spm), ZMBN 150374 (1 spm), ZMBN 150375 (1 spm), ZMBN 150376 (1 spm), ZMBN 150377 (1 spm), ZMBN 150378 (1 spm), ZMBN 150379 (1 spm), ZMBN 150380 (1 spm), ZMBN 150381 (1 spm), ZMBN 150382 (1 spm), ZMBN 150383 (1 spm), ZMBN 150384 (1 spm), ZMBN 150385 (1 spm), ZMBN 150386 (1 spm), ZMBN 150387 (1 spm), ZMBN 150388 (1 spm), ZMBN 150389 (1 spm), ZMBN 150390 (1 spm), ZMBN 150391 (1 spm), ZMBN 150392 (1 spm), ZMBN 150394 (1 spm), WS 14316 (1 spm), WS 20633 (1 spm), WS 20634 (1 spm), WS 20635 (1 spm), WS 12090 (1 spm), WS 2009 (1 spm), WS 24107 (1 spm), WS 22479 (1 spm), WS 21584 (1 spm), WS 24325 (1 spm), INV 0000810 (1 spm), INV 0002256 (1 spm), INV 0002267 (1 spm), INV 0002287 (1 spm), INV 0002257 (1 spm), USNM 43576 (2 spm), USNM 1702605 (12 spm), USNM 1702608 (3 spm), USNM 1715000 (1 spm), USNM 1714995 (5 spm), USNM 1648308 (1 spm), USNM 1714985 (1 spm), USNM 1702658 (1 spm), USNM 21611 (1 spm), USNM 41654 (1 spm), ZMMU WS 12090 (1 spm), ZMMU WS 10858 (1 spm), ZMMU WS 14316 (1 spm), ZMMU WS 20633 (1 spm), ZMMU WS 20634 (1 spm), ZMMU WS 20635 (1 spm), ZMMU WS 2009 (1 spm), ZMMU WS 24107 (1 spm), ZMMU WS 22479 (1 spm), ZMMU WS 21584 (1 spm), ZMMU WS 24325 (1 spm), ZMBN 1996 (1 spm, former *Polynoe arctica*), ZMBN 1997 (1 spm, former *Polynoe arctica*), ZMBN 1990 (1 spm, former *Polynoe foraminifera*), ZMBN 1991 (1 spm, former *Polynoe foraminifera*), ZMBN 1992 (1 spm, former *Polynoe foraminifera*), ZMBN 1987 (1 spm, former *Polynoe islandica*), ZMBN 2007 (1 spm, former *Polynoe spinulosa*).

###### Comparative material.

Eunoe s. l. depressa: holotype • USNM 5517 (1 spm), paratype • USNM 5590 (1 spm), USNM 1512664 (1 spm), USNM 1714992 (1 spm), USNM 1714994 (1 spm), USNM 1714997 (1 spm), USNM 1715001 (1 spm), USNM 1715005 (1 spm).

*Harmothoe globifera* (G.O. Sars, 1873): • ZMBN 159270 (1 spm), ZMBN 159271 (1 spm), ZMBN 159272 (1 spm), ZMBN 159273 (1 spm), ZMBN 159274 (1 spm), ZMBN 130954 (1 spm), ZMBN 104916 (1 spm), ZMBN 116515 (1 spm), ZMBN 116519 (1 spm), ZMBN 108083 (1 spm), ZMBN 120503 (1 spm), ZMBN 125403 (1 spm), ZMBN 125424 (1 spm).

*Harmothoe fragilis*: holotype • USNM 17148 (1 spm).

*Gattyana cirrhosa*: • ZMBN 117341 (1 spm), ZMBN 126064 (1 spm).

*Gattyana cf. cirrhosa*: • ZMBN 130148 (1 spm), ZMBN 136834 (1 spm), ZMBN 126075 (1 spm).

###### Diagnosis.

Elytra with long papillae along outer lateral margin; posterior margin with short, scattered papillae. Elytral macrotubercles apically arborescent, long in small specimens, becoming shorter conical or cylindrical in larger specimens. Large specimens with additional unbranched semiglobose macrotubercles with nodular papillae. Macrotubercles in posterior row. Elytrophores with extra lobe. Dorsal tubercles with extra lobe. Antennostyles, tentacular, and dorsal cirrostyles without spines. Nephridial papillae covered by nephridial sacs. Stout notochaetae with subacute tips (not truncate or capillary).

###### Description.

Redescription based on lectotype NHMO IN-5334; variation based on other material examined. Lectotype complete, 54 mm long, 11 mm wide, 37 segments. Other specimens 37–39 segments; ≤ 86 mm long and 20 mm wide. Color (ethanol): body pale yellowish-white; rarely with brownish dorsal pigmentation. Dorsal cirri with brown spot. Elytra white with brown to orange-brown spots distally. Macrotubercles brown or golden brown. Recently fixed specimens with more prominent brown spots on dorsal cirri (Fig. 5B, F).

Prostomium bilobed. Cephalic peaks conical in small specimens, weakly developed in large specimens, rarely absent. Two pairs of large eyes, anterior pair larger, located laterally or dorsolaterally at widest part of prostomium; posterior dorsal near hind margin (Fig. 5B). Palps thick, 3 × prostomial length, with six longitudinal rows of small papillae. Ceratophores of lateral antennae attached ventrally. Antennostyles with filiform papillae, spines absent; abruptly tapering subdistally. Median antenna about twice as long as laterals. Tentaculophores without distinct chaetae in lectotype; 0–2 chaetae in other examined material. Dorsal and ventral tentacular cirri subequal in length; ventral often appears shorter in fixed specimens; styles with filiform papillae, spines absent; abruptly tapering subdistally. Nuchal flap present on segment 2. Segment 2 with buccal cirri, papillate, abruptly tapering subdistally. Buccal cirri ~ 5 × longer than ventral cirri of following segments. Ventral cirri from segment 3 short, tapering, slightly papillate; not extending beyond chaetal lobes. In lectotype, pharynx everted with ring of terminal papillae (9+9), paired jaws. Facial tubercle not examined.

Elytra 15 pairs on segments 2, 4, 5, 7, 9, 11, 13, 15, 17, 19, 21, 23, 26, 29, 32. Elytrophores on elytrigerous segments with extra lobe (Fig. 5D). Elytra reniform (Fig. 5E, G). Outer lateral margin of elytra with long, dense filiform papillae. Posterior margin with short, scattered papillae (Figs 5E, 6A, 6C, 6D). Elytral surface with sparsely distributed short papillae (Fig. 6A, C, F). Elytral microtubercles low, semiglobose to weakly branched and scattered over dorsal surface, gradually increase into macrotubercles (Figs 6G, 8A–F). Elytral macrotubercles of two types. First type: conical to cylindrical, branching confined to apex, often dichotomous; more rounded with shorter branches in larger specimens (Figs 6B, 8D–F); hereafter referred to as apically arborescent macrotubercles (Fig. 6D). Second type: unbranched, semiglobose to cylindrical, distally rounded, bearing nodular papillae apically (Fig. 5E, G, 6B). Macrotubercles in posterior row (Fig. 5E, G).

Cirrigerous segments with dorsal tubercles. Dorsal tubercles with extra lobe (Fig. 5D). Cirrophores with extra lobe on one side (Fig. 5A, D). Dorsal cirrostyles ≤ 5.5 mm long; filiform papillae, spines absent, abruptly tapering subdistally.

In the lectotype, nephridial papillae from segment 4 on left side, segment 5 on right side, covered by nephridial sac, only visible if sac moved aside. In other specimens, nephridial papillae from segments 4 and 5, usually covered by nephridial sac (Figs 5C, 7E), sometimes visible in smaller specimens.

Parapodia biramous. Notopodium smaller, with ventral elongate acicular lobe. Neuropodium with elongate prechaetal acicular lobe; digitiform supra-acicular process; postchaetal lobe rounded. Acicular tips penetrate epidermis. Notochaetae numerous; wider than neurochaetae; unidentate; subacute tips, tapering to blunt point; covered with rows of small spinules almost to tip (Fig. 7C). Upper notochaetae few; short; curved. Lower notochaetae longer; straighter. Neurochaetae numerous; all unidentate; slightly falcate; with distinct rows of spinules; spinules absent distally (Fig. 7D).

Pygidium with one pair of pygidial cirri.

###### Morphological variation of material with associated molecular data.

In small specimens (body length < 5 mm; Fig. 8A, B), microtubercles are spines (occasionally bifid) and low semiglobose elements. Macrotubercles in the middle of the elytra are apically arborescent with dichotomous branching. The largest macrotubercles are slender, cylindrical, with an apical crown of short dichotomous branchlets, forming a posterior row near the posterior margin of the elytra.

Medium specimens (5–10 mm; Fig. 8C, D) differ from small specimens in the posterior macrotubercles, which become elongate conical to cylindrical with reduced apical branching, or appear unbranched and bear apical nodular papillae (Fig. 8C, D). In some small and medium specimens, elongated conical to cylindrical macrotubercles with an apically arborescent crown reach the edge of the next elytron (Fig. 8B).

Large specimens (> 10 mm; Fig. 8E, F) have both apically arborescent macrotubercles and unbranched semiglobose macrotubercles with apical nodular papillae. Macrotubercles form a row near the posterior and outer lateral margins of the elytra (Fig. 5E, G). Microtubercles are low, semiglobose to weakly branched. Occasionally, flattened microtubercles, slightly protruding above the surface of the elytra, were present, with bumps on the posterior edge, or sometimes along the entire perimeter (Figs 5D, 8F).

###### Remarks.

The detached elytra of the lectotype were assembled on a fishing line by Loshamn (Fig. 5E). As already mentioned by Loshamn (1980), *Eunoe nodosa* can be clearly distinguished from *E. oerstedi* by the presence of additional lobes on elytrophores and dorsal tubercles. In this study, we propose the presence of spines on antennostyles, tentacular, and dorsal cirrostyles (Figs 12G, 14A, 14C, 14D) as an additional diagnostic character of *E. oerstedi* versus the complete absence of spines in *E. nodosa*. The presence of spines in *E. oerstedi* was reported by Uschakov (1982), but this character was never used in identification keys. Both characters can be observed even in specimens lacking elytra, which is very common in the preserved material. Elytral characters, such as apically arborescent macrotubercles in *E. nodosa* and “horn-like” macrotubercles in *E. oerstedi*, help differentiate the species in most but not all cases. The elytral ornamentation is variable, and both species may show arborescent macrotubercles (Figs 6D, 13F, 13G), which contributed to past confusion in the use of these names. We therefore recommend prioritizing body characters for identification, using elytral characters only as an additional aid. Importantly, we retained the locality information for panels B and G (Greenland and Alaska, Fig. 5B, G) to emphasize that the diagnostic elytral morphology is consistent across large geographic distances. Another useful character is the color pattern, more obvious in the recently fixed specimens but still present in the type material. *Eunoe nodosa* has a pale yellowish-white body, rarely with a brown dorsal side (Fig. 5A, B, D), whereas *E. oerstedi* has a pattern of dark-brown spots on the dorsal side of the body. Elytra of *E. nodosa* are white with brown to orange-brown spots (Fig. 5F) and brown or golden-brown macrotubercles often arranged in a posterior row (Fig. 5E, G). In *E. oerstedi*, elytral spots are darker (Fig. 12I), and macrotubercles are dark brown, almost black in some specimens, and more irregularly distributed near the posterior margin (Fig. 12I).

*Eunoe nodosa* is often confused with *Harmothoe globifera* because both species exhibit semiglobose macrotubercles covered with nodular papillae (Figs 5E, 5G, 6B, 8D–F) (Barnich and Fiege 2010). The easiest-to-spot difference is that the elytra of *H. globifera* are white and always lack arborescent macrotubercles, whereas the elytra of *E. nodosa* always have brown spots and apically arborescent macrotubercles. If a specimen lacks elytra, it can be distinguished by the exclusively unidentate neurochaetae in *E. nodosa* and uni- and bidentate neurochaetae in *H. globifera*.

*Eunoe nodosa* can also be confused with E. s. l. depressa, *Harmothoe fragilis*, and *Gattyana cirrhosa*. We examined the holotype and the paratype of E. s. l. depressa (USNM 5517, USNM 5590) and found their morphology to be identical to that of the specimen of E. s. l. depressa included in our phylogenetic analysis, USNM 1512664 (ASGRD061-18). In E. s. l. depressa, the macrotubercles were very low, sessile mammilliform, unbranched at the tip, and did not form a posterior row (Fig. 9D–F), in contrast to the apically arborescent and semiglobose macrotubercles of *E. nodosa*, which typically form a posterior row near the posterior margin of the elytra (Fig. 5E–G). The prostomium of E. s. l. depressa closely resembled that of *E. nodosa*, with small, pointed cephalic peaks and large eyes, with the anterior pair slightly larger and positioned laterally or dorsolaterally on the widest part of the prostomium (Fig. 9B, C). These two species can be differentiated by the absence of extra lobes on the elytrophores and dorsal tubercles in E. s. l. depressa (Fig. 9A, B, D). Their notochaetae with subacute tips covered in rows of small spinules (Fig. 9G) were similar to those of most species within *Eunoe* s. str. The neurochaetae of the examined specimens were only briefly studied, and no secondary tooth was observed at the tip (Fig. 9H).

*Harmothoe fragilis* and *E. nodosa* have a similar pattern of coloration, and that might be the reason for the misidentification of SMNH 118963. Nonetheless, *H. fragilis* is differentiated from the latter by bidentate neurochaetae, absence of apically arborescent macrotubercles, and prominent cephalic peaks. In *Gattyana cirrhosa*, the arborescent macrotubercles are very similar to those of *E. nodosa*, but the clear difference is the presence of notochaetae with capillary tips in the former. Based on the elytra, the two species can be distinguished by the more uniform distribution of macrotubercles in *G. cirrhosa*.

###### Distribution.

Based on our molecular data and sequences from GenBank and BOLD (Suppl. material 1: table SS1) *Eunoe nodosa* inhabits the coastal areas of Norway, Norwegian Sea, Barents Sea, Greenland Sea, Laptev Sea, Bering Sea, and Chukchi Sea, as well as the Arctic Canadian regions including Thomas Lee Inlet, Resolute Bay, Hudson Bay, Cornwallis Island, Devon Island, Disco Island, Anaktalak Fiord, Saglek Fiord, and Sletvik. Depth range 8–501 m.

Based on morphological data and literature records (Pettibone 1963; Loshamn 1980; Uschakov 1982; Barnich and Fiege 2010), *Eunoe nodosa* is a widely spread Arctic-boreal species with a circumpolar distribution in the Arctic Ocean. It occurs from the Bering Sea to the Sea of Japan in the Western Pacific region; to New Jersey on the coast of North America in the Western Atlantic region, and as far south as the English Channel along the European coast in the Eastern Atlantic region. It was not found on the Pacific coast of North America. Depths down to 1144 m in the Greenland Sea and 100–200 m in the Barents Sea. It lives on mixed sediments, prefers silt with stones; in the temperature ranges from –1.8 °C to 7.3 °C. The paralectotype localities are Komagfjord and Varangerfjord.

##### Eunoe ciliata

Taxon classificationAnimaliaPhyllodocidaPolynoidae

(Moore, 1902) comb. nov.

97E19D17-6202-5164-8BC5-44CCF964E99B

Fig. 11

Gattyana ciliata Moore, 1902: 263, pl XIII, figs 14–19, pl. XIV, fig. 20;—Pettibone 1954: 228;—Uschakov 1982: 154, pl. LIII, figs 1–6;—Jirkov 2001: 149, figs on page 150 (1–6).

###### Material examined.

• USNM 32241 (1 spm), USNM 1077218 (1 spm), USNM 43590 (1 spm), USNM 5596 (1 spm), USNM 1512583 (1 spm).

###### Type locality.

Pacific Ocean, Alaska.

###### Comparative material.

*Gattyana cirrhosa*: • ZMBN 94800 (1 spm), ZMBN 117341 (1 spm), ZMBN 126064 (1 spm).

*Gattyana cf. cirrhosa*: • ZMBN 130148 (1 spm), ZMBN 136834 (1 spm), ZMBN 126075 (1 spm).

*Gattyana amondseni*: • ZMBN 104862 (1 spm).

###### Diagnosis.

Elytra with dense long papillae on outer lateral margin; shorter papillae on posterior margin. Long papillae scattered on surface of elytra closer to outer lateral margin. Macrotubercles conical with blunt, roughened tips occasionally covered with nodular papillae. Extra lobes on dorsal tubercles, on both sides of cirrophores, small on elytrophores. Antennostyles, tentacular, and dorsal cirrostyles without spines. Nephridial papillae visible. All notochaetae with capillary tips.

###### Description.

Based on examined material. Length ≤ 40 mm, width including chaetae 20 mm. Color in ethanol: body white (Fig. 11A), elytra tan-colored with golden macrotubercles (Fig. 11E), dorsal cirri white with brown spot (Fig. 11F).

Cephalic peaks lacking or short, blunt. Eyes small, anterior pair slightly larger than posterior, visible dorsally, on widest part of prostomium (Fig. 11A). Palps long, thin, 8 × longer than prostomium (Fig. 11C). Lateral antennal styles about twice as long as prostomium. Tentacular cirri 2–2.5 × longer than lateral antennae. Antennostyles and tentacular cirrostyles with filiform papillae, spines absent; abruptly tapering subdistally. Nuchal flap present on segment 2. Facial tubercle not examined.

Elytra 15 pairs; oval to reniform; with dense long papillae on outer lateral margin; shorter papillae on posterior margin (Fig. 11E). Elytrophores with small extra lobes (Fig. 11A). Long papillae scattered on surface of elytra closer to outer lateral margin (Fig. 11D). Microtubercles numerous, conical, pointed, hooked, and some bifid; intermediate-sized tubercles usually elongated, conical, some bifid, in several rows arranged diagonally from center of elytron laterally. Macrotubercles conical with blunt, roughened tips occasionally covered with nodular papillae, usually near posterior edge of elytra, variable in number, size, and shape (Fig. 11E).

Extra lobes on dorsal tubercles, on both sides of cirrophores (Fig. 11A). Dorsal cirrostyles with filiform papillae, spines absent; abruptly tapering subdistally (Fig. 11F).

Nephridial papillae long, slender, visible ventrally in anterior segments (Fig. 11C), directed dorsally, placed between parapodia posterior segments (making them invisible ventrally).

All notochaetae with capillary tips (Fig. 11B). All neurochaetae unidentate, covered with distinct rows of spinules, with long, bare, slightly falcate tips.

###### Remarks.

Examined specimens generally agreed with those described by Pettibone (1954) and Uschakov (1982), except for several minor characters. In our material, body length reached ≤ 60 mm, compared to 63–65 mm in Pettibone and ≤ 80 mm in Uschakov; examined specimens were incomplete, whereas Pettibone reported 36–37 segments and Uschakov reported 38–40 segments. We measured the relative length of the palps, ~ 8 × longer than the prostomium, observed extra lobes on the dorsal tubercles, on both sides of the cirrophores, and on the elytrophores, confirmed the absence of spines on the antennostyles, tentacular, and dorsal cirrostyles, and did not observe the large, dark, bulging tubercles resembling a fungoid cap reported by Uschakov on some elytra (Uschakov 1982). Examined specimens were missing tentacular cirri, Uschakov reported that tentacular cirri were 2–2.5 × longer than lateral antennae.

Although *Eunoe ciliata* comb. nov. bears notochaetae with capillary tips, it is nested within the *Eunoe* s. str. clade. *Eunoe ciliata* comb. nov. differs from the species in the *Gattyana* s. str. clade in having exclusively capillary-tipped notochaetae. All the species from the latter exhibit two types of notochaetae: with blunt tips in some notochaetae from the dorsal bundle, while all notochaetae from the ventral bundle bear capillary tips (Moore 1902; Uschakov 1982; Jirkov 2001). Another character that differentiates *E. ciliata* comb. nov. from the species in the *Gattyana* s. str. clade and combines it with *Eunoe* s. str. species is the placement of the anterior pair of eyes. All species in the *Gattyana* s. str. clade have the anterior pair of eyes located ventrally on the prostomium, making them invisible in dorsal view, similar to E. s. l. senta (Fig. 16A, C). In *E. ciliata* comb. nov., the anterior pair of eyes is located on the widest part of the prostomium and is visible dorsally (Fig. 11A). Moreover, *E. ciliata* comb. nov. and all the species from *Eunoe* s. str. clade have a prostomium with blunt or underdeveloped cephalic peaks, whereas *Gattyana* s. str. species have prominent and pointed cephalic peaks. The macrotubercles of *E. ciliata* comb. nov. lack branches, but their tips are sometimes covered with nodular papillae (Figs 4B, 11E) resembling those of *E. nodosa* (Figs 4A, 5E).

###### Distribution.

Based on sequences from GenBank and BOLD (Suppl. material 1: table SS1), *Eunoe ciliata* comb. nov. was found in the Chukchi Sea, Alaska (41 m), and San Juan Channel, WA, USA.

Based on morphological data and literature records (Uschakov 1982), *Eunoe ciliata* comb. nov. is known in the Northwestern part of the Sea of Japan (Peter the Great Bay, Tatar Strait), Sea of Okhotsk, Kuril Islands, the southeastern coast of Kamchatka, Bering Sea, Chukchi Sea, Gulf of Alaska, Vancouver Island area, and Washington, Puget Sound. Depth range 8–550 m. It prefers silty sediments (Uschakov 1982).

##### Eunoe oerstedi

Taxon classificationAnimaliaPhyllodocidaPolynoidae

Malmgren, 1865

51A57E96-B6B8-5B53-B949-33133514E9E6

Figs 10B, 12–15

Lepidonote scabra Örsted, 1843: 164–166, pl. I figs 2, 7, 10, 12, 13, 17, 18.
Polynoë scabra Theel, 1879: 7. [part]
Eunoe oerstedi Malmgren, 1865: 61, pl. 8, fig. 3A–D;—Pettibone 1954: 219, fig. 26D [part, not Eunoe barbata Moore, 1910];—Loshamn 1980: 181, fig. 89a–g;—Uschakov 1982: 177, pl. 67 figs 9–10;—Jirkov 2001: 148, figs 1–3 on page 148;—Barnich and Fiege 2010: 8, fig. 3A–H.
Harmothoe (Eunoe) oerstedi .—Pettibone 1963: 44, fig. 9d [part, not Eunoe barbata Moore, 1910].

###### Type material examined.

***Lectotype (here designated)*** • SMNH 2391 (1 spm). ***Paralectotypes*** • SMNH 2387 (2 spm), SMNH 2388 (3 spm), SMNH 2389 (1 spm), SMNH 2390 (1 spm), SMNH 2392 (2 spm), SMNH 2393 (1 spm), SMNH 2394 (4 spm), SMNH 2395 (5 spm), SMNH 2396 (3 spm), SMNH 2397 (1 spm).

###### Additional type material (not examined).

***Paralectotype*** • NHMUK 1865.9.23.21 (database type status: syntype).

###### Type locality.

Svalbard, 78.00, 20.00, 46 m, mud.

###### Other material examined.

• ZMBN 150359 (1 spm), ZMBN 150360 (1 spm), ZMBN 159262 (1 spm), ZMBN 159264 (1 spm), ZMBN 159267 (1 spm), ZMBN 159268 (1 spm), ZMBN 159269 (1 spm), ZMBN 159265 (1 spm), ZMBN 159266 (1 spm), ZMBN 159263 (1 spm), ZMBN 161425 (1 spm), ZMBN 161426 (1 spm), NTNU-VM 78191 (1 spm), INV 0000809 (1 spm), INV 0000810 (1 spm), INV 0000811 (1 spm), USNM 1666882 (1 spm), USNM 1702662 (20 spm), USNM 25185 (1 spm), USNM 26622 (1 spm), USNM 43577 (6 spm), USNM 7772 (2 spm), USNM 97536 (2 spm), USNM 97537 (3 spm), ZMBN 2003 (1 spm), ZMBN 2005 (1 spm), ZMBN 2086 (1 spm), ZMBN 2090 (1 spm), ZMBN 2111 (1 spm), ZMBN 18474 (1 spm), ZMBN 18484 (1 spm), ZMBN 18490 (1 spm), ZMBN 24938 (1 spm), ZMBN 25250 (1 spm), ZMBN 25465 (1 spm), ZMBN 27489 (1 spm), ZMBN 31553 (1 spm), ZMBN 32257 (1 spm), ZMBN 32891 (1 spm), ZMBN 36711 (1 spm), ZMBN 36712 (1 spm), ZMBN 60473 (1 spm), ZMBN 60474 (1 spm), ZMBN 60475 (1 spm), ZMBN 60476 (1 spm), ZMBN 60477 (1 spm), ZMBN 60478 (1 spm), ZMBN 60479 (1 spm), ZMBN 60480 (1 spm), ZMBN 60481 (1 spm), ZMMU WS 17144 (1 spm), ZMMU WS 10718 (1 spm), ZMMU WS 10862 (1 spm), ZMMU WS 22352 (1 spm), ZMMU WS 22364 (1 spm), ZMMU WS 11960 (3 spm).

###### Comparative material.

Eunoe s. l. senta: • USNM 17028 (1 spm), USNM 17029 (2 spm), USNM 17030 (1 spm), USNM 41649 (4 spm), USNM 5489 (1 spm), USNM 5598 (1 spm), USNM 5599 (3 spm), USNM 5600 (2 spm), USNM 5601 (1 spm), USNM 5602 (3 spm), USNM 5603 (1 spm).

###### Diagnosis.

Elytra with marginal papillae absent in larger specimens and present in smaller specimens. Microtubercles conical to cylindrical, distally simple, bifid, or multifid, becoming larger towards posterior margin of elytra; horn-like and arborescent macrotubercles present, located on posterior margin of elytra. Elytrophores, dorsal tubercles, and cirrophores lack extra lobes. Antennostyles, tentacular, and dorsal cirrostyles with filiform papillae; conical spines often present. Nephridial papillae distinct, not covered by nephridial sac. Notochaetae some ending with subacute, rounded tips and some with blunt, rounded tips.

###### Description.

Redescription based on lectotype SMNH 2391; variation based on other material examined. Lectotype complete specimen in two fragments, 70 mm long, 21 mm wide, with 38 segments in total. Other specimens of 37–39 segments, ≤ 90 mm long and 30 mm wide. Color in ethanol: lectotype pale yellowish white (Fig. 12A). Macrotubercles and some microtubercles dark brown, rest of microtubercles white (Fig. 12H). Some paralectotypes with white macrotubercles. Recently fixed specimens with dorsal brown segmental pattern, every other parapodium with brown spot, dorsal cirri variable from white to brown, nephridial papillae often brown (Fig. 12B, D, F). Macrotubercles dark brown (Fig. 12I). Spines and chaetae golden-colored (Fig. 12).

Prostomium bilobed. Cephalic peaks weakly developed, rarely absent. Two pairs of large eyes, anterior pair slightly larger, located dorsally on widest part of prostomium (Fig. 12A, B). In lectotype, prostomium with two palps, two lateral antennae, median antenna missing. Ceratophores of lateral antennae attached ventrally. Palps thick, 3 × longer than prostomium, with six longitudinal rows of small papillae. In lectotype, antennal styles with filiform papillae, but no obvious spines; abruptly tapering subdistally. Paralectotypes and other examined specimens with conical spines with pointed tips on antennostyles (Fig. 12B); abruptly tapering subdistally. Median antenna ~ 2 × longer than lateral. In lectotype, tentaculophores attached on both sides of prostomium, each with dorsal and ventral tentacular cirri with filiform papillae, without spines; abruptly tapering subdistally. Paralectotypes and other specimens with spines on tentacular cirrostyles (Fig. 12B); abruptly tapering subdistally. Ventral tentacular cirri slightly shorter than dorsal. In lectotype, two notochaetae present only on left tentaculophore, acicula present on both sides. In other specimens, tentaculophores with 0–2 notochaetae. Nuchal flap present on segment 2. Buccal cirri on segment 2 abruptly tapering subdistally; 5 × longer than ventral cirri in following segments. Ventral cirri from segment 3 not extending beyond chaetal lobe. In lectotype, first ventral cirrus on left side longer than on right side, and half of buccal cirrus present (possibly a malformation). In other examined specimens, all ventral cirri of approximately same length. In lectotype, pharynx not everted, cut out from ventral side for DNA extraction. In other specimens, pharynx with ring of papillae (9+9) and jaws. Facial tubercle not examined.

Fifteen pairs of elytra, on segments 2, 4, 5, 7, 9, 11, 13, 15, 17, 19, 21, 23, 26, 29, 32. Elytrophores without extra lobe (Fig. 12A–D). Elytra reniform anteriorly, becoming more rounded in mid-body. Outer lateral margin of elytra with numerous papillae in small specimens; smooth or with only sparse short papillae in large specimens. Elytral surface with scattered papillae. Elytral macrotubercles of two types. First type, apically arborescent macrotubercles: conical to cylindrical, with non-dichotomous branching confined to apex; irregular apical crown of short, sharp processes (Figs 12I, 13F, 13G). Second type, horn-like macrotubercles: tall conical, with dominant main axis; occasionally with lateral branch (monopodial appearance); hereafter referred to as horn-like macrotubercles (Figs 12H, 12I, 13A–E). Macrotubercles increase in size towards posterior edge of elytra. Microtubercles scattered over elytral surface, conical spines (Fig. 13I), and apically bifid or multifid cones (Fig. 13H).

Dorsal tubercles and cirrophores lack extra lobe (Fig. 12A–D). Dorsal cirrostyles ≤ 7 mm long, with filiform papillae, conical spines may be present (Figs 12G, 14A, 14C, 14D); abruptly tapering subdistally. Spines less obvious in large specimens and may not be present on all cirri. Lectotype with spines on cirri from segment 32 and on detached cirri found in the jar, presumably belonging to same specimen.

Nephridial papillae from segment 5. Obvious in lectotype and other specimens, longer than 5 mm (Figs 12E, 12F, 14E). In smaller specimens, nephridial papillae may be covered by nephridial sac.

Parapodia biramous. Notopodium smaller, with ventral elongate acicular lobe. Neuropodium with elongate prechaetal acicular lobe and a digitiform supra-acicular process; postchaetal lobe rounded. Aciculae tips penetrating epidermis. Notochaetae numerous, wider than neurochaetae, covered with small rows of spinules, some ending with subacute, rounded tips (Fig. 14F, G) and some with blunt, rounded tips (Fig. 14G). Few upper notochaetae short and curved, lower notochaetae longer, straighter. Neurochaeta numerous, all unidentate with slightly falcate tips, covered with distinct rows of spinules (Fig. 14B).

Lectotype with only one pygidial cirrus, second missing; other specimens with two cirri.

###### Morphological variation of material with associated molecular data.

Small specimens (body length < 5 mm; Fig. 15A, B) are dominated by apically arborescent macrotubercles: conical to cylindrical tubercles with branching confined to the apex, forming an irregular, non-dichotomous crown of short, sharp branchlets. Occasionally, one branch is more prominent than the others, producing a weakly monopodial appearance. Posterior macrotubercles become larger, giving the elytra a spiny appearance.

Medium specimens (5–10 mm; Fig. 15C, D) show two macrotubercle morphotypes. Apically arborescent macrotubercles remain common, whereas the largest macrotubercles become horn-like, i.e., tall conical tubercles with a dominant main axis and only occasional lateral branching (monopodial appearance).

Large specimens (> 10 mm; Fig. 15E, F) are dominated by very tall conical horn-like macrotubercles with a dominant axis, occasionally bearing a lateral branch. Apically arborescent macrotubercles with non-dichotomous branching are less frequent and smaller (Fig. 15F, G).

Microtubercles in all specimens were spines, occasionally bi- and multifid.

###### Remarks.

The description of *Eunoe oerstedi* provided by Barnich and Fiege (2010) was based on two Svalbard specimens. Our study re-examined the original SMNH type series and designated a lectotype, thereby stabilizing the application of the name. We additionally provide an expanded redescription based on the lectotype and a broad set of newly examined material, documenting size-related variation in elytral marginal papillae, present in smaller specimens and reduced or absent in larger ones, and in elytral macrotubercle morphology, with apically arborescent and horn-like morphotypes dominating in different size classes. Finally, we clarify the variability of conical spines on the antennae, tentacular cirri, and dorsal cirri, confirm the consistent absence of extra lobes on elytrophores, dorsal tubercles, and cirrophores, and link these morphological observations to specimens with associated molecular data, including new sequences generated from the lectotype SMNH 2391, improving diagnosis and separation of *E. oerstedi* from the frequently confused *E. nodosa*.

*Eunoe oerstedi* can be distinguished from *E. nodosa* by the lack of extra lobes on elytrophores and dorsal tubercles (present in *E. nodosa*, Fig. 5D; absent in *E. oerstedi*, Fig. 12A–D) and the presence of spines on the antennostyles, tentacular, and dorsal cirrostyles, which are usually present but may be reduced or absent in some specimens. *Eunoe oerstedi* and *E. nodosa* were often confused in the past when identified based on elytra (Barnich and Fiege 2010). The large specimens of these species can be differentiated by the brown horn-like macrotubercles in the former and macrotubercles assembled in a row on the posterior edge resembling semiglobose macrotubercles without branches covered with nodular papillae in the latter. Elytra of smaller specimens are easily confused due to the presence of the arborescent macrotubercles in both species.

However, the apically arborescent macrotubercles in *E. oerstedi* differ from those in *E. nodosa* in having conical and pointed non-dichotomous branches. We recommend using the characters on the elytra only as an additional identification aid because *E. oerstedi* and *E. nodosa* can be clearly distinguished by body characters. Recently fixed specimens of *E. oerstedi* can be easily distinguished by the specific brown pattern on the dorsal side of the body, dark-brown macrotubercles, and brown nephridial papillae on the ventral side of the body, while most of the other *Eunoe* s. str. species have white or light brown bodies and golden-brown macrotubercles (except *E. cf. oerstedi* CMC01).

*Eunoe oerstedi* can be confused with Eunoe s. l. senta (Loshamn 1980), whose type locality was first published as Greenland and later corrected to Alaska (Moore 1905). This possibly led to the assumption that E. s. l. senta occurs in the Arctic Ocean and the coast of Norway (Loshamn 1980). Upon examining the specimens identified by Pettibone as *Okudahermadion senta*, we suggest they belong to Eunoe s. l. senta. This species can be distinguished by golden antler-like, long-branched arborescent macrotubercles with distally pointed branchlets that resemble “chicken feet” (Fig. 16D) in contrast to the dark-brown horn-like macrotubercles of *E. oerstedi*. It is also different from *E. oerstedi* in the placement of nephridial papillae, which are not visible ventrally (Fig. 16F). Another differentiating characteristic is that the anterior pair of eyes is twice as large as the posterior pair and is located ventrally (Fig. 16C, E). The latter unites E. s. l. senta with *Gattyana* s. str. species and may validate the original description of *E. senta* as *Gattyana*, even though it does not exhibit the traditionally used *Gattyana* character of notochaetae with capillary tips (Fig. 16G).

The species *Eunoe barbata* (Fig. 21), *E. hozawai* Okuda, 1939, *E. sentiformis* Uschakov, 1958 (Fig. 20), and *E. shirikishinai* (Fig. 19) all resemble *E. oerstedi*. However, they differ from the latter by the absence of spines on the antennostyles, tentacular, and dorsal cirrostyles and by the truncated tips of notochaetae versus subacute to rounded tips in *E. oerstedi*.

*Eunoe oerstedi*, *E. cf. oerstedi* CMC01, and *E. spinicirris* have spines on the cirrostyles. In *E. oerstedi*, the spines are conical with pointed tips. In *E. cf. oerstedi* CMC01, the spines are numerous and cylindrical, with rounded tips (Fig. 17G, J). In *E. spinicirris*, the spines are conical with sharp tips and are also present on the elytra (Fig. 18B, E, F).

###### Distribution.

Based on our molecular data and sequences from GenBank and BOLD (Suppl. material 1: table SS1), *Eunoe oerstedi* has been found in the Arctic regions: New Brunswick (Canada), Norwegian Sea, Greenland Sea, Barents Sea, White Sea, and Laptev Sea. Depth range 3–487 m.

Based on morphological data and literature records (Pettibone 1963; Loshamn 1980; Uschakov 1982; Barnich and Fiege 2010), *Eunoe oerstedi* is widely distributed in the Arctic Ocean and extends along the western Atlantic coast of North America to Rhode Island. In the Pacific Ocean, it is replaced by *E. cf. oerstedi* CMC01 (from the Bering Sea to the Yellow Sea) and *E. barbata* (from the Bering Sea to the coast of California). Depth range 10–945 m. The paralectotype localities are Hornsundöyene, Bellsund, Isfjorden, Treurenberg bay, and Storfjorden.

##### Eunoe cf. oerstedi

Taxon classificationAnimaliaPhyllodocidaPolynoidae

CMC01

04AD8FB1-8600-5678-879D-D958E404C668

Fig. 17

###### Material examined.

• USNM 31410 (1 spm), USNM 30014 (9 spm).

###### Description.

Based on examined material. Length ≤ 35 mm, width including parapodia, 15 mm. Color: recently fixed specimens with dorsal brown segmental pattern, every other parapodia with brown spot (Fig. 17H), dorsal cirri variable from white to brown, nephridial papillae often brown (Fig. 17I). Spines and chaetae golden (Fig. 17E, G). Macrotubercles dark brown and golden brown (Fig. 17C, D).

Cephalic peaks weakly developed. Two pairs of large eyes, anterior pair located laterally, on widest part of prostomium. Palps thick, 3–3.5 × longer than prostomium. Median antenna about twice as long as laterals. Tentacular cirri subequal. Antennostyles, tentacular and dorsal cirrostyles covered with filiform papillae and numerous long, cylindrical spines with blunt tips that sometimes bifurcate (Fig. 17J); abruptly tapering subdistally. Nuchal flap present on segment 2. Facial tubercle not examined.

Fifteen pairs of elytra. Extra lobes on elytrophores absent (Fig. 17A). Elytra reniform in anterior part of body, becoming more round in mid-body (Fig. 17B). Outer lateral margin of elytra with short papillae, surface with scattered short papillae. Macrotubercles numerous, cylindrical, with flattened, crown-like tip with undulate margin, often appearing as coronate discs in top view (Fig. 17C), and occasional apically arborescent macrotubercles (Fig. 17D). Macrotubercles cover most surface of elytra, becoming large on posterior edge.

Extra lobes on dorsal tubercles and cirrophores absent (Fig. 17A).

Nephridial papillae visible ventrally (Fig. 17I).

Notochaetae numerous, wider than neurochaetae, covered with small rows of spinules, ending with blunt, truncate, or subacute tips (Fig. 17E, F). Neurochaetae numerous, all unidentate, covered with distinct rows of spinules (Fig. 17E). Neurochaetae with slightly falcate tips.

###### Remarks.

The specimens with BOLD Process IDs: BENTH162-08, BENTH235-08, BENTH236-08, and BENTH239-08 (BOLD:AAE4839, *Eunoe cf. oerstedi* CMC01 (Carr et al. 2011)) from the Bering Strait, which we included in the phylogenetic reconstruction, had dried out and were not suitable for morphological analysis (Sarah L. Mincks, pers comm.). A single photograph of the specimen representing this clade available in BOLD shows numerous long golden spines with blunt tips on the dorsal cirri. The material examined in the present study was collected from the West Black Hill, Bering Sea (USNM 30014), and Alaska (USNM 31410) and presumably belongs to the same species as *Eunoe cf. oerstedi* CMC01 reported by Carr et al. (2011). *Eunoe cf. oerstedi* CMC01 and *E. oerstedi* share the same color pattern (Figs 12B, 12D, 12F, 17H, 17I); however, they can be distinguished by the shape of the spines and macrotubercles. The examined specimens bear numerous long, cylindrical spines with blunt tips on the antennostyles, tentacular, and dorsal cirrostyles (Fig. 17G, J), whereas the spines in *E. oerstedi* are scarcer and conical with pointed tips (Fig. 14A, C). Additionally, *E. oerstedi* bears horn-like and sometimes apically arborescent macrotubercles without secondary branches on the posterior edge of the elytra. However, in *E. cf. oerstedi* CMC01, numerous cylindrical macrotubercles with flattened, crown-like tip with undulate margins, often appearing as coronate discs in top view, and apically arborescent macrotubercles (Fig. 17C, D), similar to macrotubercles in *E. nodosa*, were present, covering the entire elytra, with larger ones located on the posterior edge.

###### Distribution.

Based on sequences from GenBank and BOLD (Suppl. material 1: table SS1), *Eunoe cf. oerstedi* CMC01 was found in the Chukchi Sea (Alaska), Bering Strait, and Bering Sea. Depth range 53–57 m. Based on morphological data, *Eunoe cf. oerstedi* CMC01 was reported in the Chukchi Sea and Pacific Ocean from the Bering Sea to the Sea of Okhotsk. Depth range 0–57 m.

##### Eunoe spinicirris

Taxon classificationAnimaliaPhyllodocidaPolynoidae

Annenkova, 1937

241EF8D0-FA55-5123-A61F-01DC6B0873FE

Fig. 18

Eunoe spinicirris Annenkova, 1937: 150, text-fig. 4, pl. I fig. 7, pl. II fig. 12, pl. III fig. 24, pl. IV figs 31, 32.—Uschakov 1982: 181, T. LXV, figs 1–5.—Imajima 1961: 85, fig. 2a–g.

###### Type locality.

Pacific Ocean, northern Sea of Japan.

###### Material examined.

• USNM 43601 (1 spm), USNM 43578 (1 spm).

###### Diagnosis.

Large specimens lack fringe of filiform papillae on margins and surface of elytra. Antennostyles, tentacular, and dorsal cirrostyles, and elytra with numerous large, pointed spines. Nephridial papillae visible ventrally. Extra lobes on dorsal tubercles, elytrophores, and cirrophores absent. Stout notochaetae with pointed and truncate tips.

###### Description.

Based on examined material. Length ≤ 40 mm, width including parapodia, 25 mm, with 38–42 segments. Color in ethanol: body light brown with white longitudinal stripe (Fig. 18D), brown spot on first visible segment medially (Fig. 18A), dorsal cirri white, elytra tan with large brown and purple spots, spines golden brown (Fig. 18B).

Cephalic peaks lacking. Eyes large. Anterior pair of eyes larger than posterior, located laterally, on widest part of prostomium. Palps thick, 4–4.5 × longer than prostomium, with six longitudinal rows of small papillae. Median antenna with large ceratophore; style about twice as long as lateral antenna styles. Dorsal tentacular cirrus subequal to ventral. Styles of antennae, tentacular and dorsal cirri abruptly tapering subdistally (Fig. 18A), with occasional thin filiform papillae and numerous large, pointed spines (Fig. 18E). Tips of spines rarely bifurcate. Ventral cirri from segment 3 short, do not reach beyond chaetal lobe. Dorsal cirri extend beyond chaetal tips. Extra lobes on dorsal tubercles, elytrophores, and cirrophores absent.

Elytra 15 pairs on segments 2, 4, 5, 7, 9, 11, 13, 15, 17, 19, 21, 23, 26, 29, 32. Elytra covered with large spines, increasing on posterior end, tips occasionally bifurcate (Fig. 18B). Large specimens lack fringe of filiform papillae on margins and surface of elytra.

Nephridial papillae start from segment 4, visible ventrally (Fig. 18C).

All notochaetae covered with small rows of spinules ending—some with subacute tips, some with truncate tips (Fig. 18G). All neurochaetae unidentate, covered with distinct rows of spinules, with long, bare, and slightly falcate tips.

###### Remarks.

Uschakov (1982) described specimens reaching ≤ 80 mm in length with 38–42 segments; the examined specimens were smaller (≤ 40 mm). Uschakov also described the tentacles and dorsal cirri as bearing soft, elongated papillae and having sharply filiform tips; in our specimens, the styles were abruptly tapering subdistally and bore only occasional thin filiform papillae in addition to the numerous large, pointed chitinous spines. On the elytra, Uschakov reported occasional low spines with a stellate apex and tridentate spinules, which we did not observe in our specimens.

*Eunoe spinicirris* is easily distinguished from other congeners by the numerous large, pointed spines that cover the antennostyles, tentacular, and dorsal cirrostyles, and elytra (Fig. 18B, E, F). Our phylogenetic analysis includes the COI sequence from BOLD derived from the specimen with Process ID BENTH238-08 (Alaska, USA) (Fig. 1). Although this specimen was unavailable for examination, we assume it was correctly identified because of its unique character—very large, pointed spines. The photograph of this specimen in BOLD is blurry, but the large spines on the dorsal cirri and elytra are visible. The comment on BENTH238-08 in BOLD states that the specimen had bright purple and green elytra, which also corresponds with the examined USNM specimens, as they had purple spots on elytra, and the green coloration might disappear in alcohol. Interestingly, the spines of *Eunoe spinicirris*, *E. oerstedi*, and *E. cf. oerstedi* CMC01 sometimes bifurcate distally (Figs 12G, 17J).

###### Distribution.

Based on a single record from BOLD (Suppl. material 1: table SS1), *Eunoe spinicirris* is found in the Chukchi Sea (Alaska) at 57 m.

Based on morphological data and literature records (Imajima 1961; Imajima and Hartman 1964; Uschakov 1982), *Eunoe spinicirris* is a North Pacific Asian species distributed along the Asian coast from the Chukchi Sea to the Peter the Great Bay in the Sea of Japan. Depth range 20–240 m. It prefers a silt bottom with small pebbles (Uschakov 1982).

##### Eunoe shirikishinai

Taxon classificationAnimaliaPhyllodocidaPolynoidae

Imajima & Hartman, 1964

1EE4A8C5-EFD4-51FD-9811-4893CB8AA8FA

Fig. 19

Eunoe shirikishinai Imajima & Hartman, 1964: 30, pl. 3, figs a–d.

###### Type material examined.

***Holotype*** • USNM 74051 (1 parapodium, 1 elytron).

###### Type locality.

Hokkaido Island, Shirikishinai, Japan.

###### Comparative material.

*Eunoe sentiformis*: • USNM 43582 (1 spm).

*Eunoe spinosa*: • USNM 23762 (1 spm).

###### Diagnosis.

Cirrophores with extra lobes. Elytral macrotubercles sharply pointed with short branches all ending with sharp, pointed tips, near posterior edge of elytra. Dorsal cirrus without spines. Notochaetae with truncate tips bearing a long spinous, minutely serrated region.

###### Description.

Based on examined material (one elytron and one parapodium). Color in alcohol: elytra white with brown spot, macrotubercles golden brown.

Cirrophore with extra lobe. Dorsal cirrus without spines; abruptly tapering subdistally.

Elytron reniform (Fig. 19A). Microtubercles horn-like and bifurcated spines on anterior and median areas of elytra. Macrotubercles spiniform, sharply pointed with short branches, all ending with sharp, pointed tips, near posterior edge of elytron (Fig. 19A, B). Outer lateral and posterior margin with short, scarce papillae.

All notochaetae covered with small rows of spinules, ending with truncate tips with long, spinous, minutely serrated region (Fig. 19E). All neurochaetae unidentate, covered with distinct rows of spinules, with long, bare, and slightly falcate tips (Fig. 19D).

###### Remarks.

The holotype of *Eunoe shirikishinai* (USNM 74051, from Hokkaido, Japan) comprised only a single parapodium and one elytron, with the body absent. Therefore, our redescription above is restricted to characters observable from these fragments. All other characters for the species are taken verbatim from the original description by Imajima and Hartman (1964) and could not be verified. Those include body size, length 57 mm, width including parapodia 20 mm; segment number, 38 segments; absence of cephalic peaks; position of the anterior eyes, laterally, on the widest part of the prostomium; and palps, thick, ~3.5× longer than lateral antennae, with triangular papillae arranged in six longitudinal rows. Additional head structures, such as nuchal flap and facial tubercle, extra lobes, spines on body appendages, and papillation on the elytral surface were not reported by Imajima and Hartman (1964). In addition, spines were not observed on the preserved holotype dorsal cirrus. The presence or absence of extra lobes on elytrophores and dorsal tubercles cannot be assessed from the available material. However, the cirrophore of the examined holotype parapodium had an extra lobe.

*Eunoe shirikishinai* has been previously confused with five other species of *Eunoe* due to superficial similarity in macrotubercles: *E. hozawai*, *E. oerstedi*, *E. senta*, *E. sentiformis*, and *E. spinosa*. For phylogenetic reconstruction, we used data from BOLD derived from the specimens *E. cf. oerstedi* P_8 NJ-2021 (BOLD Process ID GBMNF750-22), from the Kumano Sea, Japan (Jimi et al. 2021), and *E. oerstedi* isolate PhB172 (BOLD process ID GBAN4646-13), from South Korea, both identified as *E. shirikishinai* (Jimi et al. 2021). These specimens formed a well-supported clade nested within the *Eunoe* s. str. clade. However, we cannot conclusively identify them as *E. shirikishinai* because the photograph provided by Jimi et al. (2021) lacks an image of the elytra, but the specimen has extra lobes on the cirrophores. The elytra bear the main identifying character, which is the golden-brown branching, sharply pointed macrotubercles (Fig. 19B). This character unites *E. shirikishinai* with *E. hozawai*, the latter differing only in having smaller and shorter macrotubercles (Imajima and Hartman 1964).

The *Eunoe sentiformis* specimen (USNM 43582 from the Kuril Islands, North-West Pacific Ocean) exhibited two types of macrotubercles. The first type: cylindrical macrotubercles, ending distally in a rosette-shaped apex with a strongly lobate margin, a “flower-like” head with five to eight petals, which Uschakov (1958) described as “mushroom-like” (Fig. 20B–C). The second type: cylindrical macrotubercles with a dentate crown at the tip (Fig. 20D). In Imajima and Hartman (1964), these species were distinguished by the shape of notochaetae ending in truncate tips with a long spinous, minutely serrated region in *E. shirikishinai* and not serrated, truncate tips in *E. sentiformis*. Upon comparing the notochaetae of USNM 74051 (*E. shirikishinai*) and USNM 43582 (*E. sentiformis*), we found no obvious differences between the two specimens (Figs 19E, 20E, 20G). *Eunoe sentiformis* has some macrotubercles similar to *E. senta*, but it is different in the shape of notochaetae and displays lateral placement of the anterior pair of eyes and golden-brown branching macrotubercles (Uschakov 1982).

The specimen USNM 23762 (Hakodate Bay, Japan), identified by Pettibone as *Eunoe oerstedi*, might be *E. spinosa* based on the presence of extra lobes on elytrophores and cirrophores, not dorsal tubercles (Fig. 19G), and pointed notochaetae (Fig. 19H). This specimen was similar to *E. shirikishinai* in having macrotubercles with short, sharp branches (Fig. 19F) and lacking spines on the dorsal cirri (Fig. 19I, J).

###### Distribution.

Hokkaido, Japan (Imajima and Hartman 1964), Kumano Sea, Japan; South Korea (Jimi et al. 2021); and Hakodate Bay, Japan at 3.5 m depth.

##### Eunoe barbata

Taxon classificationAnimaliaPhyllodocidaPolynoidae

Moore, 1910

C1491DAE-351E-519D-993C-0346C023D45C

Fig. 21

Eunoe barbata Moore, 1910: 334, pl. 28, figs 1–6. — Imajima and Hartman 1964: 29, pl. 2, figs a–f.— Uschakov 1982: 182, T. LXVII, figs 1–4.— Jirkov 2001: 145, figs 1–4 on page 145.

###### Type material examined.

***Paratype*** • USNM 17291 (1 spm).

###### Type locality.

Pacific Ocean, Admiralty Inlet, Washington; Santa Cruz, California

###### Material examined.

• USNM 43580 (1 spm), USNM 17291 (1 spm), USNM 25201 (2 spms), USNM 25203 (3 spms), USNM 25205 (4 spms), USNM 25191 (1 spm), USNM 25195 (1 spm), USNM 25189 (1 spm), USNM 25194 (1 spm), USNM 25187 (1 spm), USNM 25198 (1 spm), USNM 25190 (3 spms), USNM 25199 (1 spm), USNM 25193 (1 spm), USNM 25185 (1 spm), USNM 25196 (3 spms), USNM 25200 (1 spm), USNM 25192 (2 spms), USNM 25197 (1 spm), USNM 25186 (2 spms), USNM 25204 (3 spms), USNM 25188 (1 spm), USNM 25202 (4 spms), USNM 25207 (18 spms).

###### Diagnosis.

Elytra with fringe of filiform papillae on outer lateral margin, with tuft of significantly longer papillae on mid-posterior margin. Elytral macrotubercles cylindrical to slightly clavate, apically coronate with small, rounded branches, forming crenulate apex; located near posterior edge of elytra. Antennostyles, tentacular, and dorsal cirrostyles with dense, filiform papillae; without spines. Extra lobes absent on elytrophores and dorsal tubercles; present on cirrophores. Notochaetae with truncate tips.

###### Description.

Based on examined material. Length ≤ 41 mm, width including parapodia 13 mm, with 38 segments. Color in ethanol: body tan with brown longitudinal stripe (Fig. 21A); antennostyles, tentacular, and dorsal cirrostyles white with brown spot distally (Fig. 21A, B); nuchal flap brown (Fig. 21A); nephridial papillae sometimes brown (Fig. 21A); elytra white with brown spot, macrotubercles golden brown (Fig. 21B).

Cephalic peaks absent in some specimens, present as small and pointed in others. Eyes large. Anterior pair of eyes larger than posterior, located laterally or dorsolaterally on widest part of prostomium. Palps thick, covered with six longitudinal rows of papillae, 4.5 × longer than prostomium, 2 × longer than lateral antennae (Fig. 21A). Antennal styles covered with dense filiform papillae; without spines. Tentacular cirri subequal; styles with dense filiform papillae; without spines. Styles of antennae, tentacular cirri abruptly tapering subdistally. Nuchal flap present on segment 2. Facial tubercle not examined.

Elytra 15 pairs on segments 2, 4, 5, 7, 9, 11, 13, 15, 17, 19, 21, 23, 26, 29, 32. Extra lobes on elytrophores absent. Elytra rounded to reniform; with fringe of filiform papillae on outer lateral margin, with distinct tuft of significantly longer papillae on mid-posterior margin (Fig. 21C, G). Numerous furcate microtubercles and rare, simple spines present on anterior and median parts of elytra. Macrotubercles cylindrical to slightly clavate, apically coronate with small branches with rounded tips, forming crenulate apex; located near posterior edge of elytra (Fig. 21B, G).

Dorsal tubercles without extra lobe. Cirrophores with extra lobe on one side (Fig. 21B). Dorsal cirri covered with numerous long filiform papillae, spines absent; abruptly tapering subdistally (Fig. 21D).

Nephridial papillae often inconspicuous ventrally, sometimes visible (Fig. 21E).

All notochaetae covered with small rows of spinules ending with truncate tips (Fig. 21F). All neurochaetae unidentate, covered with distinct rows of spinules, with long, bare, and slightly falcate tips.

###### Remarks.

The examined material of *Eunoe barbata* agrees well the descriptions by Moore (1910), Imajima and Hartman (1964), and Uschakov (1982) in overall body size (≤ 41 mm), the number and arrangement of elytra (15 pairs), densely papillate antennostyles, tentacular, and dorsal cirrostyles, abruptly tapering subdistally, a long marginal fringe on elytra with a distinct tuft of longer papillae posteriorly, and the chaetal morphology, notochaetae with truncate tips; neurochaetae unidentate with a long, bare, slightly falcate tip. In addition, we document variation in the cephalic peaks, which are absent in some specimens but small and pointed in others, and clarify the elytral ornamentation by distinguishing the abundant furcate microtubercles from the macrotubercles, which are cylindrical to slightly clavate, apically coronate with rounded branch tips, and located along the posterior edge of the elytra. Earlier accounts described the elytral macrotubercles as knoblike, bearing on the summit two, three, or more stout, sharp points, sometimes surrounded by a raised ring (Moore 1910), as irregular, with pointed, minutely denticulated branches (Imajima and Hartman 1964), or as elongated spinules with a denticulate or stellate apex (Uschakov 1982). We also record the presence of extra lobes on cirrophores, the absence of extra lobes on elytrophores and dorsal tubercles, and the absence of spines on the antennostyles and cirrostyles.

Pettibone (1954) synonymized *Eunoe barbata* with *E. oerstedi*. However, the species was treated as valid by Imajima and Hartman (1964), Uschakov (1982), and Jirkov (2001). Uschakov (1982) disagreed with the synonymy proposed by Pettibone (1954) and stated that the specimens from Hokkaido, Japan, and Puget Sound, Washington, were in fact *E. barbata*. We examined specimens from the western coast of the USA, identified by Pettibone as *E. oerstedi* in 1954, and found them to belong to *E. barbata*. Despite their close resemblance, these two species can be differentiated by the absence of spines on the antennostyles, tentacular, and dorsal cirrostyles, the presence of extra lobes on the cirrophores, and the truncate tips of notochaetae in *E. barbata* (Fig. 21D, F) versus the presence of spines on the antennostyles, tentacular, and dorsal cirrostyles, the absence of extra lobes, and subacute tips of notochaetae in *E. oerstedi* (Uschakov 1982). Additionally, the filiform papillae on the edge of the elytra of *E. barbata* form a thick fringe with a prominent tuft of significantly longer papillae (Fig. 21C). Furthermore, papillae on the dorsal cirri are more numerous in *E. barbata* than in *E. oerstedi* and may appear as a white coating, likely due to detritus adhering to the papillae (Fig. 21D). These species can be further differentiated by their macrotubercles, which are cylindrical to slightly clavate and apically coronate with small rounded branches forming a crenulate apex in *E. barbata*, whereas *E. oerstedi* has “horn-like” macrotubercles that are broader at the base and bear few or no apical branches, although some overlap in shape may occur (Fig. 4C, G). Uschakov (1982) also pointed out the difference in the length of the median antenna style being shorter in *E. barbata* than in *E. oerstedi*. However, this character could not be evaluated consistently here because median antennae were missing in most examined specimens of *E. barbata*, and in the few specimens with intact median antennae, the relative length overlapped with *E. oerstedi*. While the morphological data suggest that *E. barbata* is a valid species, molecular data are lacking to corroborate this conclusion.

We studied the specimen USNM 43580 (Strait of La Perouse, Sea of Okhotsk, North-West Pacific Ocean) identified as *E. barbata* by Annenkova (Fig. 21H–J). This specimen exhibited dorsal cirri sparsely covered with filiform papillae with spines having an intermediate shape between *E. oerstedi* and *E. cf. oerstedi* CMC01, but the macrotubercles were more similar to those of *E. barbata*, and *E. cf. oerstedi* CMC01, and the tips of notochaetae were truncate. This suggests that the West Pacific specimens might represent a closely related but different species from *E. barbata*, *E. oerstedi*, and *E. cf. oerstedi* CMC01. This requires further clarification with molecular data.

###### Distribution.

Based on morphological data and literature records (Imajima and Hartman 1964; Uschakov 1982), *Eunoe barbata* occurs in the Eastern Pacific region from the Bering Sea to the coast of California. Depth range 0–180 m. It prefers pebbles and stones (Uschakov 1982).

## Discussion

In this study, we aimed to partially revise *Eunoe* by integrating newly obtained and previously published molecular and morphological data. Given the scarcity of molecular data for many species, our analyses focused on the Arctic fauna of *Eunoe* and included a limited number of species from other regions. Nonetheless, we attempted to make the most comprehensive assessment possible with the data currently at hand.

Our phylogenetic results show that the traditional broad concepts of *Eunoe* and *Gattyana* are polyphyletic, consistent with previous studies. Furthermore, our results corroborate previous phylogenetic studies in which *Harmothoe* was recovered as polyphyletic, further supporting the need for revision of this genus (Norlinder et al. 2012; Shields et al. 2013; Gonzalez et al. 2018, 2023; Zhang et al. 2018; Gastaldi 2019; Bonifácio and Menot 2019; Jimi et al. 2021; Murray et al. 2025).

The type species of both *Eunoe* and *Gattyana* were included in our analysis. The clades that included the type species are herein referred to as *Eunoe* sensu stricto and *Gattyana* s. str. (Fig. 1). Species currently assigned to *Eunoe* and *Gattyana* but falling outside the respective s. str. clades are referred to here as *Eunoe* sensu lato and *Gattyana* s. l. for convenience. These designations are provisional and do not imply that the excluded species form natural groups. The distinction between *Eunoe* and *Gattyana* has traditionally been based on the morphology of notochaetae. *Eunoe* species were characterized by thick notochaetae, whereas *Gattyana* species were considered to bear mostly capillary notochaetae (Uschakov 1982; Pettibone 1963; Jirkov 2001; Barnich and Fiege 2009, 2010). The placement of *Eunoe ciliata* comb. nov. (former *Gattyana ciliata*) within the *Eunoe* s. str. clade with high support suggests that notochaetal morphology does not effectively separate these genera. A potential distinguishing feature could be the position of the anterior pair of eyes on the prostomium. In all species comprising the *Gattyana* s. str. clade in our analysis, the anterior pair of eyes was located ventrolaterally, making them invisible from the dorsal view (Fig. 16B, C). In contrast, the species of the *Eunoe* s. str. clade, including *E. ciliata* comb. nov. (Fig. 11A), have their anterior pair of eyes positioned dorsally or laterally.

We did not identify any exclusive character combination that differentiates *Eunoe* s. str. from other polynoid genera. Nevertheless, species in the *Eunoe* s. str. clade can be characterized by a combination of dorsolateral or dorsal anterior eyes and thick, stiff, opaque elytra with conspicuous ornamentation. In most species, this ornamentation takes the form of branching macrotubercles, except for *E. spinicirris*, in which prominent elytral ornamentation is expressed as large spines. This diagnosis excludes most *Eunoe* s. l. taxa recovered outside *Eunoe* s. str. in our analyses, which have thin, soft, and often translucent elytra without macrotubercles, except for *Eunoe* s. l. d*epressa* bearing rigid elytra with small macrotubercles.

Based on the examined material and data available in GenBank and BOLD, the clade *Eunoe* s. str. includes six species: *E. nodosa*—the type species of the genus, *E. oerstedi*, *E. cf. oerstedi* CMC01, *E. spinicirris*, *E. shirikishinai*, and *Eunoe ciliata* comb. nov. (Fig. 1). Species boundaries within this clade are supported by congruence among morphology, phylogeny, ASAP delimitation results, and haplotype networks.

Our study focused on the two species of *Eunoe* common in the Nordic seas: *E. nodosa* and *E. oerstedi*. We clarified their diagnoses based on examination of the type specimens and non-type material, including both morphological and molecular data. The lectotypes for both species were designated in the present study to stabilize the use of the species’ names in the future. Importantly, we obtained DNA sequence information of the lectotype of *E. oerstedi*, linking the species name to type DNA. In addition, we described in detail the morphological characters that clearly differentiate *E. nodosa* from *E. oerstedi*. For rapid screening, the difference in elytral macrotubercles and color patterns (Figs 5B, 5F, 12B, 12D) in recently fixed specimens may be useful. *Eunoe oerstedi* bears conical, horn-like macrotubercles in addition to apically arborescent macrotubercles found in both species (Figs 4C, 12I, 13A–G). However, we emphasize that color and elytral sculpture can vary with preservation and specimen condition, and elytral ornamentation may also vary within an individual depending on position on the elytron and along the body, as shown in other polynoids by Valencia-Soto (2021). Reliable identifications can only be based on a combination of traits. *Eunoe oerstedi* has visible and pigmented nephridial papillae (Fig. 12F), which are hidden under a sac in *E. nodosa* (Fig. 5C), and lacks extra lobes on dorsal tubercles and elytrophores (Fig. 12D), present in *E. nodosa* (Fig. 5D). Moreover, *E. oerstedi* has short and sharp spines on the antennae and dorsal cirri (Figs 12G, 14A, 14C, 14D), while only soft, elongated papillae are present on body appendages in *E. nodosa* (Figs 5D, 7A, 7B).

*Eunoe cf. oerstedi* CMC01 from Alaska was recovered by Carr et al. (2011) as a potentially new species different from *E. oerstedi* from the Northwest Atlantic based on COI data. We examined the specimens from USNM that matched a photographed specimen in BOLD (BOLD:AAE4839), also from Alaska, which allowed us to find morphological differences between the two species. *Eunoe oerstedi* had conical spines with pointed tips on the dorsal cirri and antennae, whereas *E. cf. oerstedi* CMC01 had elongated, cylindrical spines with rounded tips (Fig. 17G, J). Molecular and morphological data suggest that *E. cf. oerstedi* CMC01 is a potentially new species. However, in the absence of materials suitable for morphological examination, we refrain from a formal description of this species.

Within *Eunoe* s. str., *Eunoe spinicirris*, *E. oerstedi*, and *E. cf. oerstedi* CMC01 form a well-supported monophyletic clade. These three species have spines on antennae and dorsal cirri. In *E. spinicirris*, spines also occur on the elytra and replace prominent elytral macrotubercles.

The species currently assigned to *Eunoe* but recovered outside *Eunoe* s. str. require further study. Together, *Eunoe* s. l. was recovered as multiple species-level groups forming seven clades distributed across the tree (Fig. 1). These groups are frequently associated with other polynoid genera, including *Harmothoe*. Notably, Eunoe s. l. depressa falls between two presumably cryptic, morphologically very similar *Harmothoe cf. rarispina* lineages, being sister to one of them (Fig. 1A).

Across the sampled *Eunoe* s. l. taxa, elytral morphology shows a marked contrast with *Eunoe* s. str. Eunoe s. l. depressa is morphologically the closest to *Eunoe* s. str., sharing the presence of small macrotubercles on thick, opaque elytra and a dorsolateral placement of the anterior eyes. In contrast, the remaining species have soft elytra and lack macrotubercles. The main exception is E. s. l. etheridgei, which bears macrotubercles despite having soft, translucent elytra. Interestingly, E. s. l. etheridgei formed a well-supported clade with E. s. l. issunboushi (Fig. 1B), despite the absence of any apparent exclusive morphological characters uniting them, and despite the complete lack of elytral macrotubercles in the latter. The scattered placement of Antarctic, Australian, North Atlantic, and North Pacific *Eunoe* s. l. taxa across several clades highlights the need for broader geographic and taxonomic sampling to clarify the generic boundaries of *Eunoe* and related taxa and to determine which morphological characters are most informative at the generic level.

Notably, clade C, which comprises predominantly deep-water Southern Hemisphere species, also includes the North Atlantic deep-water species Eunoe s. l. bathydomus (Fig. 1C). In addition, E. s. l. leiotentaculata and E. s. l. albacauda form a well-supported clade (Fig. 1D) and share a distinctive morphology that differs from both *Eunoe* s. str. and all other examined *Eunoe* s. l. taxa. They are characterized by very large eyes, the absence of papillae on palps and antennae, and soft elytra (Murray et al. 2025).

We also examined the Northern Hemisphere *Eunoe* s. l. specimens available in the USNM collections that lack molecular data. When material was unavailable, we assessed species based on original descriptions and subsequent redescriptions. *Eunoe hozawai*, *E. barbata*, and *E. sentiformis* are very similar to *E. shirikishinai* nested within the *Eunoe* s. str. clade, sharing truncated notochaetae and having dorsal cirri covered with filiform papillae without spines. The difference between these species is in the way of branching of their macrotubercles (Fig. 4; Table 2). Furthermore, *E. hydroidopapillata* and *E. spinosa* may also be included in *Eunoe* s. str. based on the presence of the branching macrotubercles on rigid elytra and dorsolateral anterior eyes. Although, *E. hydroidopapillata* is described as lacking dorsal tubercles, which requires additional verification (Rzhavsky and Shabad 1999). Based on the original description, *E. tritoni* may represent a junior synonym of *E. nodosa*, as its elytral macrotubercles fall within the variation observed in *E. nodosa* (McIntosh 1900). This remains untested because we did not examine *E. tritoni* material and lack molecular data from the type locality (Faroe Channel).

**Table 2.** Discriminatory characters of species in *Eunoe* sensu stricto and Eunoe sensu lato senta.

**Species**	***Eunoe nodosa* (M. Sars, 1861)**	***Eunoe ciliata* comb. nov. (Moore, 1902)**	***Eunoe oerstedi* Malmgren, 1865**	***Eunoe cf. oerstedi* CMC01**	***Eunoe spinicirris* Annenkova, 1937**	***Eunoe shirikishinai* (Imajima & Hartman, 1964)**	***Eunoe barbata* Moore, 1910**	***Eunoe sentiformis* Uschakov, 1958**	**Eunoe s. l. senta (Moore, 1902)**
**Sources**	Loshamn 1980; Uschakov 1982; Pettibone 1963; Barnich and Fiege 2010, this study	Uschakov 1982, this study	Loshamn 1980; Uschakov 1982; Pettibone 1963; Barnich and Fiege 2010, this study	this study	Uschakov 1982; Imajima 1961; Imajima and Hartman 1964, this study	Imajima and Hartman 1964, this study	Uschakov 1982; Imajima and Hartman 1964, this study	Uschakov 1982; Imajima and Hartman 1964, this study	Loshamn 1980; Imajima and Hartman 1964, this study
**Type locality**	North Cape, Norway	Pacific Ocean, Alaska	Svalbard	–	Sea of Japan	Hokkaido, Japan	Washington; California	East Siberian Sea	Chukchi Sea, Alaska
**Size of eyes**	Large, anterior larger	Small, anterior slightly larger	Large, anterior slightly larger	Large	Large, anterior slightly larger	?	Large, anterior slightly larger	Large, anterior larger	Large, anterior larger
**Anterior pair of eyes (placement)**	Laterally or dorsolaterally	Dorsally	Dorsally	Laterally	Laterally	Laterally	Laterally or dorsolaterally	Laterally	Ventrally, under cephalic peaks
**Cephalic peaks**	Conical to weakly developed, rarely lacking	Lacking or weakly developed, short, and blunt	Weakly developed, rarely lacking	Weakly developed	Absent	Absent	Small, pointed, or absent	Absent	Rounded
**Palp/prostomium length ratio**	3	8	3	3–3.5	4–4.5	3.5 × as long as lateral antennae	4.5	~3.5	5–6
**Spines on antennostyles, tentacular, and dorsal cirral styles**	Absent	Absent	Conical with pointed tips, sometimes bifurcate	Long, cylindrical with blunt tips, sometimes bifurcate	Conical with pointed, sharp tips, sometimes bifurcate	?	Absent	Absent	Absent
**Extra lobe on dorsal tubercles, elytrophores, and/or cirrophores**	Dorsal tubercles, elytrophores, and cirrophores (one side)	Dorsal tubercles, elytrophores, and cirrophores (both sides)	Absent	Absent	Absent	Cirrophores	Cirrophores (one side)	Elytrophores and cirrophores (both sides, swollen basally)	Cirrophores (both sides, swollen basally)
**Nephridial papillae**	Covered with nephridial sac	Long and slender, visible ventrally in anterior part of body, covered posteriorly	Visible, pigmented	Visible, pigmented	Visible	?	Visible and pigmented or covered with nephridial sac	Covered with nephridial sac	Covered with nephridial sac
**Main type of macrotubercles**	1: Apically arborescent (conical to cylindrical, branching confined to apex, often dichotomous; more rounded with shorter branches in larger specimens) 2: semiglobose to cylindrical, distally rounded with nodular papillae	Conical with blunt, rounded tips, occasionally covered with nodular papillae	1: Apically arborescent (conical to cylindrical, with non-dichotomous branching confined to apex; irregular apical crown of short, sharp processes) 2: Horn-like (tall conical, with dominant main axis; occasionally with lateral branch (monopodial appearance))	1: Cylindrical, with flattened, crown-like tip with undulate margin (appearing as coronate discs in top view) 2: Occasional apically arborescent	Large spines	Spiniform, sharply pointed with short branches, all ending with sharp, pointed tips	Cylindrical to slightly clavate, apically coronate with small branches with rounded tips, forming crenulate apex	1: Cylindrical, ending distally in rosette-shaped apex with strongly lobate margin (“flower-like” head with five to eight petals) 2: Cylindrical, with dentate crown at tip	Antler-like, arborescent with sharp long branches
**Color of macrotubercles**	Brown	Golden brown	Dark brown	Dark brown	Golden brown	Golden brown	Golden brown	Golden brown	Golden
**Papillae on elytral margin**	Long dense on outer lateral margin; short, more scattered on posterior margin	Long on outer lateral margin; short on posterior margin	Absent or short and scarce on outer lateral margin in larger specimens, less scarce in small specimens	Short on outer lateral margin	Absent in larger specimens	Short, scarce on outer lateral and posterior margins	Long on outer lateral margin; short on posterior margin; with tuft of significantly longer papillae on mid-posterior margin	Long and short, scattered on outer margin	Long; posterior margin with short scattered papillae
**Papillae on elytral surface**	Short, scattered scarcely all over surface	Long, scattered on surface closer to outer lateral margin	Short, scattered scarcely all over surface	Short, scattered scarcely all over surface	Absent in larger specimens	?	Short, scattered scarcely all over surface	Short, scattered scarcely all over surface	?
**Notochaetae tips**	Subacute, rounded	Capillary	Pointed, rounded, or blunt, rounded	Blunt, truncate, or subacute, rounded	Pointed, rounded, or blunt, truncate	Blunt, truncate with long spinous minutely serrated region	Truncate or blunt, rounded	Blunt, truncate with unserrated or weakly serrated region	Thin pointed
**Coloration of body**	White, dorsal cirri white with brown spot	White, dorsal cirri white with brown spot	Brown dorsally, every other parapodium with brown spot	Brown dorsally, every other parapodium with brown spot	Pale brown with white longitudinal stripe	?	Tan with brown longitudinal stripe	White	White
**Coloration of elytra**	White with brown spots	White	Tan with brown and black spots	White with brown and purple spots	Tan with large brown and purple spots (green on freshly fixed)	White with brown spot	White with brown spot	White	White

*Eunoe clarki* (holotype USNM 21984) and *Eunoe spinulosa* (holotype USNM 7758; USNM 7762; USNM 1151330) lack macrotubercles on elytra, suggesting they might not belong to *Eunoe* s. str. *Eunoe uniseriata* holotype (USNM 36273) is damaged and lacks elytra, and is here referred to as incertae sedis. Based on the original descriptions, *E. hubrechti*, *E. laetmogonensis*, *E. subtruncata*, and *E. purpurea* are best treated as *Eunoe* s. l., as they do not match the combination of rigid, opaque elytra with conspicuous macrotubercles and either have extremely large eyes or an unusual placement of the anterior eyes.

The status of *E. yedoensis* remains uncertain because published illustrations suggest a spiny elytral surface (resembling *E. spinicirris*), but available descriptions do not document elytral thickness (McIntosh 1885; Izuka 1912). The status of *Eunoe senta* is also uncertain due to its golden macrotubercles, resembling those of *Gattyana* s. str., and ventral placement of the anterior pair of eyes (Fig. 16A, C). Moreover, *E. senta* was originally described as *Gattyana* (Moore 1902), which shows the need for molecular data to clarify its taxonomic placement.

Therefore, in addition to the six species included in the *Eunoe* s. str. clade based on molecular data, *E. barbata*, *E. hydroidopapillata*, *E. hozawai*, *E. sentiformis*, and *E. spinosa* may also belong to the genus based on the dorsolateral position of the anterior eyes and the presence of thick, opaque elytra with macrotubercles. We provide the identification key with illustrations to the eleven *Eunoe* s. str. species (Suppl. material 3).

In conclusion, our study demonstrates that *Eunoe* s. l. and *Gattyana* s. l. are polyphyletic. *Gattyana* s. str. is characterized by the combination of ventrally positioned anterior eyes and the presence of capillary notochaetae. In contrast, *Eunoe* s. str. lacks an obvious, unique diagnostic character across all sampled taxa. We propose a diagnosis for *Eunoe* s. str. based on dorsolateral to dorsal anterior eyes combined with thick, stiff, opaque elytra bearing conspicuous ornamentation (macrotubercles or spines). Including more *Eunoe* and *Harmothoe* species in the analysis, especially taxa with diverse elytral morphologies, would allow testing whether the proposed diagnostic characters are consistently associated with *Eunoe* s. str. and would facilitate the future reassignment of *Eunoe* s. l. species.

In the present study, *Eunoe* is restricted to *Eunoe* s. str. and the generic diagnosis is revised accordingly. Species referred to here as *Eunoe* s. l., including those not examined here, cannot be confidently reassigned to other genera. We therefore provisionally retain these species in *Eunoe* pending formal generic reassignment. As shown in this study, traditional generic diagnostic characters, such as chaetal morphology, are often not supported by molecular data. A comprehensive revision of the most common and species-rich polynoid genera, based on much broader taxon sampling, is needed to clarify generic boundaries and composition, and to enable explicit tests of character homology and character evolution.

## Supplementary Material

XML Treatment for Eunoe

XML Treatment for Eunoe nodosa

XML Treatment for Eunoe ciliata

XML Treatment for Eunoe oerstedi

XML Treatment for Eunoe cf. oerstedi

XML Treatment for Eunoe spinicirris

XML Treatment for Eunoe shirikishinai

XML Treatment for Eunoe barbata

## Additional information

### Artificial Intelligence (AI) use

The authors accept full responsibility for the content of the manuscript, including the disclosure of any use of AI.

Regarding the use of AI in the preparation of this manuscript, the authors declare the following: GPT, Used for: Language, style and writing.

We used OpenAI’s ChatGPT (GPT-4) for grammar and style suggestions. This tool did not generate content or rewrite scientific meaning.

### Author ORCIDs

M.W. Everett https://orcid.org/0009-0001-2957-9402

A. Koroleva https://orcid.org/0000-0002-2410-0554

N. Straube https://orcid.org/0000-0001-7047-1084

N. Budaeva https://orcid.org/0000-0001-9748-2285

### Data availability

All of the data that support the findings of this study are available in the main text or Supplementary Information.

## Supplementary materials

Supplementary material 1 Sampling data and PCR protocol Data type xlsx Explanation note **table SS1**. List of material used in this study with original and new identifications, sampling information, museum numbers, voucher numbers, GenBank accession numbers, and BOLD BIN, Process and Sample IDs. **table SS2**. Primers and PCR settings for COI, 16S, ITS2, and 28S. https://binary.pensoft.net/file/1703764This dataset is made available under the Open Database License (http://opendatacommons.org/licenses/odbl/1.0/). The Open Database License (ODbL) is a license agreement intended to allow users to freely share, modify, and use this Dataset while maintaining this same freedom for others, provided that the original source and author(s) are credited. Martha W. Everett, Anna Koroleva, Nicolas Straube, Nataliya Budaeva

Supplementary material 2 Expanded Maximum Likelihood and Bayesian Inference phylogenetic trees Data type pdf Explanation note Maximum likelihood (ML) and Bayesian Inference (BI) trees based on the concatenated dataset of COI, 16S, ITS2, and 28S. Results of ASAP species delimitation. https://binary.pensoft.net/file/1703765This dataset is made available under the Open Database License (http://opendatacommons.org/licenses/odbl/1.0/). The Open Database License (ODbL) is a license agreement intended to allow users to freely share, modify, and use this Dataset while maintaining this same freedom for others, provided that the original source and author(s) are credited. Martha W. Everett, Anna Koroleva, Nicolas Straube, Nataliya Budaeva

Supplementary material 3 Key to *Eunoe* sensu stricto with illustrations Data type pdf https://binary.pensoft.net/file/1703766This dataset is made available under the Open Database License (http://opendatacommons.org/licenses/odbl/1.0/). The Open Database License (ODbL) is a license agreement intended to allow users to freely share, modify, and use this Dataset while maintaining this same freedom for others, provided that the original source and author(s) are credited. Martha W. Everett, Anna Koroleva, Nicolas Straube, Nataliya Budaeva
