# Supplementary material for: Eunoe Malmgren, 1865 (Annelida, Polynoidae) in the Arctic, North Atlantic, and North Pacific: redescription of the type species and clarification of the genus boundaries
Source: Zookeys. 2026 Jul 1;1283:343–403. doi: 10.3897/zookeys.1283.168195 (PMC13347114; doi:10.3897/zookeys.1283.168195)
Supplement: Supplementary material 2 — Expanded Maximum Likelihood and Bayesian Inference phylogenetic trees [file zookeys-1283-343_article-168195__-s002.pdf]

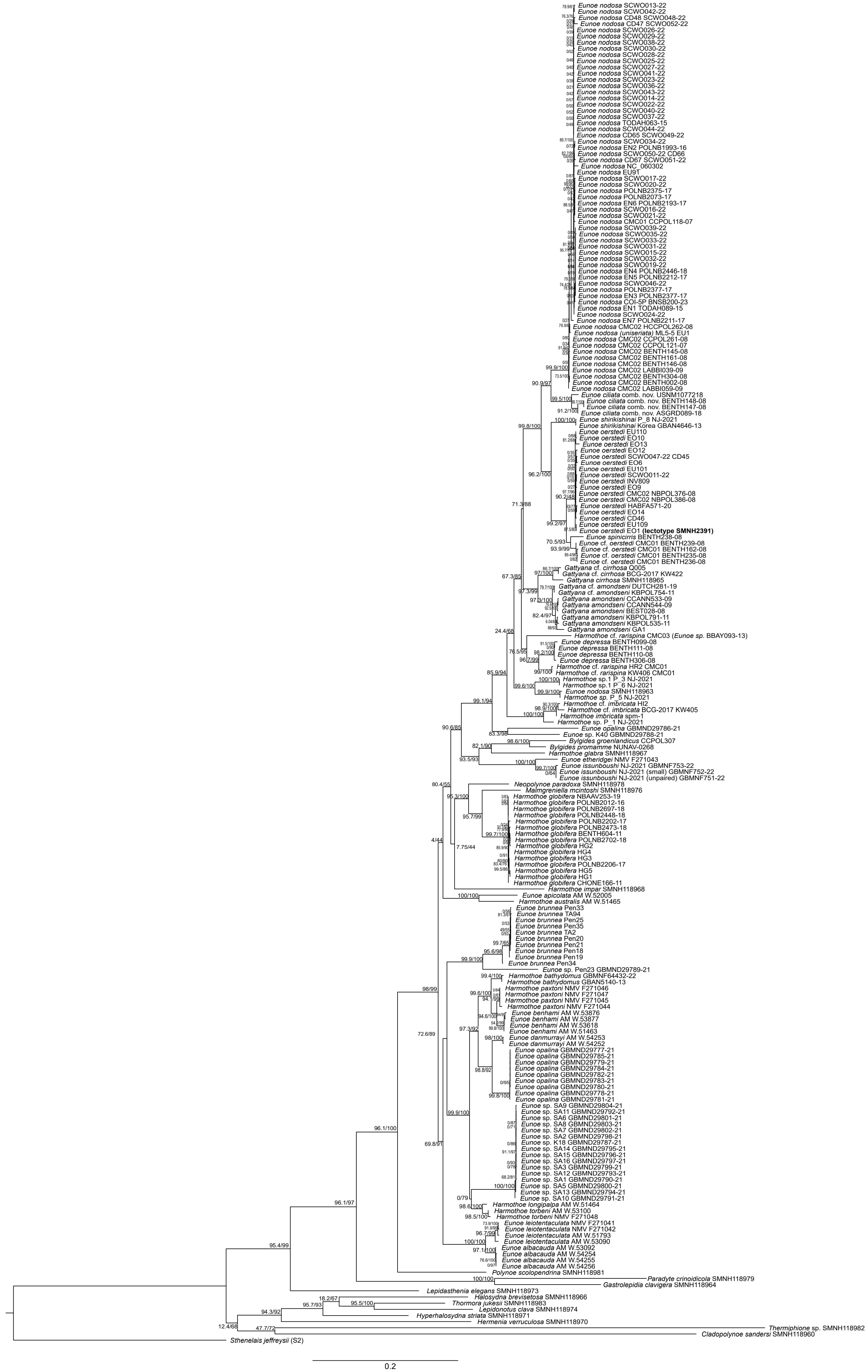

Figure S1. Non-collapsed ML Eunoe tree. Node support is given as SH-aLRT support / ultrafast bootstrap support

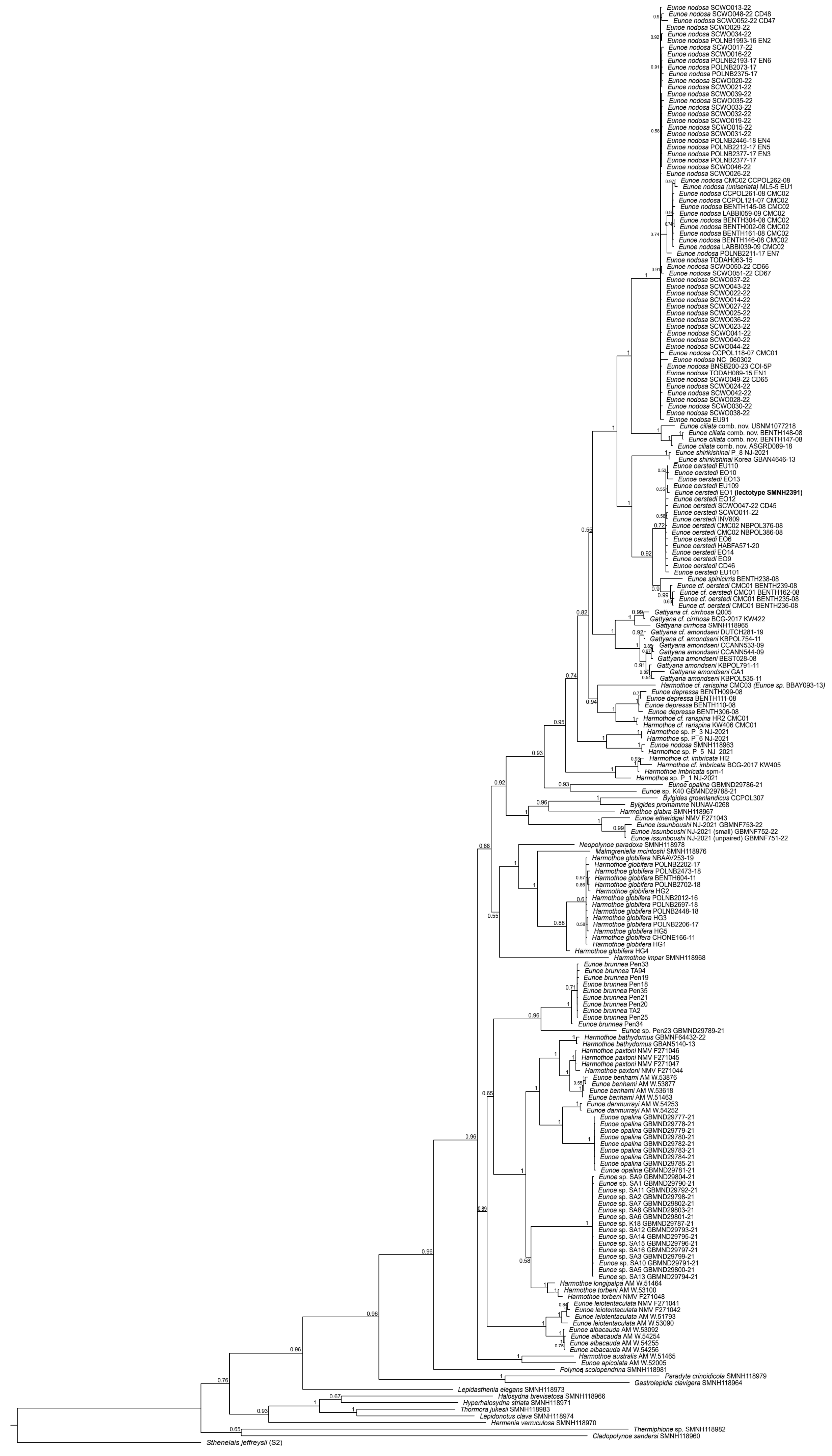

Figure S2. Non-collapsed BI Eunoe tree. Posterior probabilities (PP) are shown above branches

File S1. ASAP species delimitation results

A) ASAP results for CO1 fragment

Partition 1

Asap-Score: 2.500000

Proba: 1.337325e-01

Nb subsets with recursion:6 (without recursion: 5)

Eunoe oerstedi [1] n: 14 ;id: SCWO011_22 EU110 EO13 EO10 SCWO047_22_CD45 EO1 INV809 EU109 NBPOL376_08_Eunoe_oerstedi_CMC02_HQ024019 NBPOL386_08_Eunoe_oerstedi_CMC02_HQ024020 EO14 EO6 HABFA571_20 EO12

Eunoe nodosa [2] n: 60 ;id: TODAH089_15_Eunoe_nodosa_EN1 SCWO029_22 SCWO024_22 SCWO028_22 SCWO038_22 SCWO044_22 SCWO049_22_CD65 SCWO037_22 SCWO043_22 SCWO022_22 SCWO014_22 TODAH063_15_Eunoe_nodosa SCWO027_22 SCWO025_22 SCWO036_22 SCWO023_22 SCWO040_22 SCWO026_22 POLNB2193_17_Eunoe_nodosa_EN6 SCWO016_22 SCWO020_22 SCWO021_22 POLNB2073_17_Eunoe_nodosa POLNB2375_17_Eunoe_nodosa SCWO041_22 NC_060302_Eunoe_nodosa POLNB2446_18_Eunoe_nodosa_EN4 POLNB2212_17_Eunoe_nodosa_EN5 SCWO046_22 SCWO019_22 SCWO039_22 POLNB2377_17_Eunoe_sp_EN3 SCWO032_22 SCWO031_22 POLNB2377_17_Eunoe_sp SCWO033_22 SCWO015_22 BNSB200_23_Eunoe_nodosa_COI_5P SCWO042_22 SCWO035_22 SCWO013_22 SCWO017_22 POLNB1993_16_Eunoe_nodosa_EN2 SCWO034_22 SCWO050_22_CD66 SCWO051_22_CD67 SCWO030_22 CCPOL118_07_Eunoe_nodosa_CMC01_HQ024300 POLNB2211_17_Eunoe_nodosa_EN7 CCPOL121_07_Eunoe_nodosa_CMC02_HQ024301 BENTH146_08_Eunoe_nodosa_CMC02_HM473738 CCPOL261_08_Eunoe_nodosa_CMC02_HQ024302 LABBI039_09_Eunoe_nodosa_CMC02_HQ023872 BENTH304_08_Eunoe_nodosa_CMC02_HM473735 BENTH002_08_Eunoe_nodosa_CMC02_HM473736 BENTH161_08_Eunoe_nodosa_CMC02_HM473739 LABBI059_09_Eunoe_nodosa_CMC02_HQ023873 BENTH145_08_Eunoe_nodosa_CMC02_HM473737 CCPOL262_08_Eunoe_nodosa_CMC02_HQ024303 MK390764_Eunoe_nodosa_uniseriata_ML5_5_EU1

Eunoe shirikishinai [3] n: 2 ;id: Eunoe_shirikishinai_cf_oerstedi_P_8_NJ_2021 GBAN4646_13_Eunoe_oerstedi_shirikishinai_Korea_JX503009

Eunoe ciliata comb. nov. [4] n: 4 ;id: USNM1077218_Eunoe_Gattyana_ciliata BENTH148_08_Eunoe_Gattyana_ciliata_HM473746 BENTH147_08_Eunoe_Gattyana_ciliata_HM473745 ASGRD089_18_Eunoe

Eunoe cf. *oerstedi* [5] n: 4 ;id: BENTH162_08_Eunoe_oerstedi_CMC01_HM473740 BENTH235_08_Eunoe_oerstedi_CMC01_HM473741 BENTH236_08_Eunoe_oerstedi_CMC01_HM473742 BENTH239_08_Eunoe_oerstedi_CMC01_HM473743

Eunoe spinicirris [6] n: 1 ;id: BENTH238_08_Eunoe_spinicirris_HM473744

B) ASAP results for 16S fragment

Partition 1

Asap-Score: 2.000000

Proba: 2.069805e-02

Nb subsets with recursion:3 (without recursion: 2)

Eunoe oerstedi [1] n: 9 ;id: SCWO011_22 SCWO047_22_CD45 EO9 EO12 CD46 EO10 EO14 EO6 EO1

Eunoe nodosa [2] n: 19 ;id: TODAH089_15_Eunoe_nodosa_EN1 SCWO042_22 SCWO028_22 SCWO048_22_CD48 SCWO050_22_CD66 SCWO038_22 SCWO029_22 SCWO044_22 SCWO049_22_CD65 SCWO052_22_CD47 NC_060302_Eunoe_nodosa POLNB1993_16_Eunoe_oerstedi_EN2 POLNB2193_17_Eunoe_nodosa_EN6 SCWO016_22 SCWO020_22 SCWO021_22 POLNB2446_18_Eunoe_nodosa_EN4 POLNB2212_17_Eunoe_oerstedi_EN5 SCWO046_22

Eunoe shirikishinai [3] n: 1 ;id: Eunoe_cf_oerstedi_P_8_NJ_2021

C) ASAP results for ITS2 fragment

Partition 1

Asap-Score: 2.000000

Proba: 7.624750e-01

Nb subsets with recursion:3 (without recursion: 2)

Eunoe oerstedi A [1] n: 8 ;id: SCWO011_22 SCWO047_22_CD45 EO6 EO9 INV809 EO14 CD46 EO12

Eunoe oerstedi B [2] n: 2 ;id: EO10 EO1

Eunoe nodosa [3] n: 18 ;id: TODAH089_15_Eunoe_nodosa_EN1 POLNB1993_16_Eunoe_oerstedi_EN2 POLNB2446_18_Eunoe_nodosa_EN4 POLNB2212_17_Eunoe_oerstedi_EN5 POLNB2193_17_Eunoe_nodosa_EN6 SCWO042_22 SCWO028_22 SCWO048_22_CD48 SCWO050_22_CD66 SCWO051_22_CD67 SCWO046_22 SCWO038_22 SCWO016_22 SCWO029_22 SCWO020_22 SCWO044_22 SCWO049_22_CD65 SCWO037_22

D) ASAP results for 28S fragment

Partition 1

Asap-Score: 1.000000

Proba: 1.157685e-01

Nb subsets with recursion:4 (without recursion: 3)

Eunoe oerstedi [1] n: 14 ;id: SCWO011_22 INV809 SCWO047_22_CD45 EO14 EO12 EO6 CD46 EU110 EU101 EO10 EO13 EO9 EO1 EU109

Eunoe nodosa [2] n: 25 ;id: TODAH089_15_Eunoe_nodosa_EN1 POLNB1993_16_Eunoe_oerstedi_EN2 POLNB2446_18_Eunoe_nodosa_EN4 POLNB2212_17_Eunoe_oerstedi_EN5 SCWO028_22 SCWO048_22_CD48 SCWO050_22_CD66 SCWO051_22_CD67 SCWO046_22 SCWO038_22 SCWO016_22 SCWO029_22 SCWO044_22 SCWO049_22_CD65 SCWO037_22 SCWO052_22_CD47 SCWO021_22 SCWO019_22 SCWO039_22 POLNB2377_17_Eunoe_sp_EN3 EU91 POLNB2193_17_Eunoe_nodosa_EN6 SCWO042_22 SCWO020_22 POLNB2211_17_Eunoe_oerstedi_EN7

Eunoe shirikishinai [3] n: 1 ;id: Eunoe_cf_oerstedi_P_8_NJ_2021

Eunoe ciliata comb. nov. [4] n: 1 ;id: USNM1077218_Gattyana_ciliata
