# Supplementary material for: Eunoe Malmgren, 1865 (Annelida, Polynoidae) in the Arctic, North Atlantic, and North Pacific: redescription of the type species and clarification of the genus boundaries
Source: Zookeys. 2026 Jul 1;1283:343–403. doi: 10.3897/zookeys.1283.168195 (PMC13347114; doi:10.3897/zookeys.1283.168195)
Supplement: Supplementary material 3 — Key to Eunoe sensu stricto with illustrations [file zookeys-1283-343_article-168195__-s003.pdf]

Key to *Eunoe* sensu stricto with illustrations

1	Elytra with very long, soft, dichotomously branched macrotubercles, hydroid-like in appearance, and with a deep oval to reniform cavity near posterior margin on ventral side<br><br>Dorsal tubercles absent	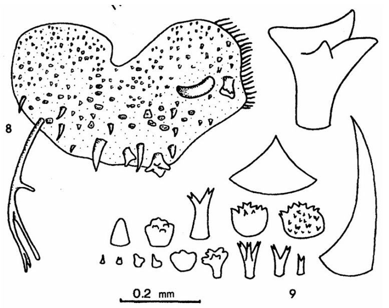<br>(from Rzhavsky & Shabad 1999)	***Eunoe hydroidopapillata* Rzhavsky & Shabad, 1999**<br>(Kamchatka)
–	Elytra without long hydroid-like macrotubercles and without deep cavity<br><br>Dorsal tubercles present		**2**
2	Notochaetae capillary (Fig. 11B)	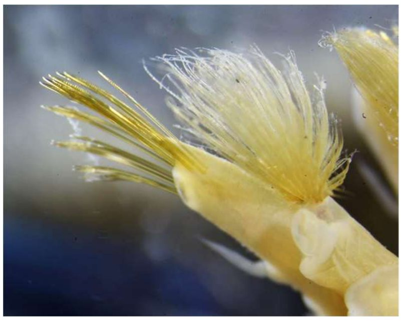	***Eunoe ciliata* comb. nov. (Moore, 1902)**<br>(North Pacific Ocean)
–	Notochaetae stout		**3**
3	Elytrophores, dorsal tubercles, and cirrophores without extra lobes<br><br>Spines on antennostyles, tentacular, and dorsal cirrostyles present	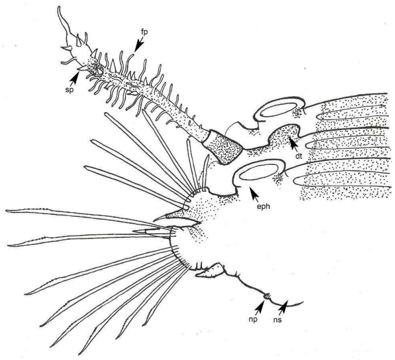<br>Abbreviations: dt – dorsal tubercle, eph – elytrophore, fp – filiform papillae, np – nephridial papilla, ns –	**4**

		nephridial sac, sp – spine.	
–	At least one of the structures (elytrophores, dorsal tubercles, and cirrophores) with extra lobes<br><br>Spines on antennostyles, tentacular, and dorsal cirrostyles absent	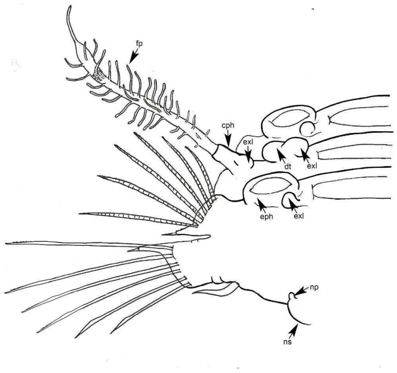<br>Abbreviations: dt – dorsal tubercle, eph – elytrophore, exl – extra lobe, fp – filiform papillae, np – nephridial papilla, ns – nephridial sac.	**6**
4	Branched elytral macrotubercles absent<br><br>Large spines with sharp tips on antennostyles, tentacular, and dorsal cirrostyles, and elytra (Fig. 18B, E, F)	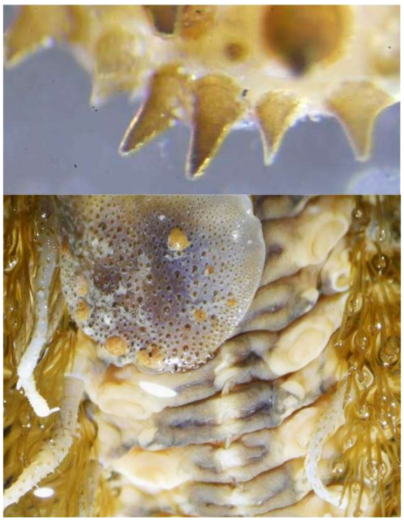	***Eunoe spinicirris*** **Annenkova, 1937** (North Pacific Ocean)
–	Branched elytral macrotubercles present		**5**

5	Conical spines with sharp tips on antennostyles, tentacular, and dorsal cirrostyles (can be present only on a few dorsal cirri and antennae) (Figs 12G, 14A, C, D)<br><br>Elytra with dark brown horn-like macrotubercles (Figs 12H, I, 13A-E), occasionally with apically arborescent macrotubercles (Figs 12I, 13F, G)<br><br>Dorsal tubercles and cirrophores often brown (Fig. 12D). Nephridial papilla visible, often brown (Fig. 12F)	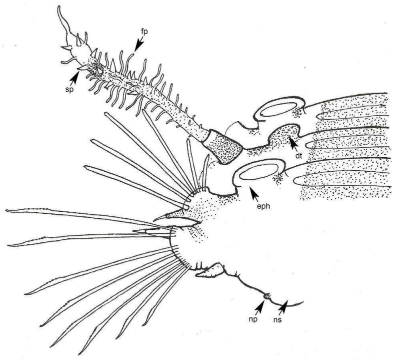 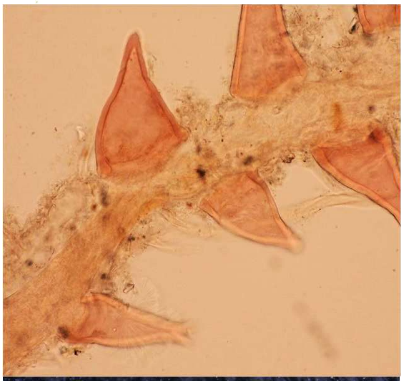 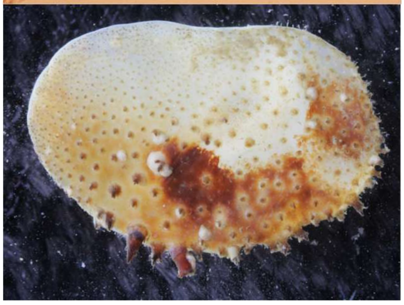	***Eunoe oerstedi* Malmgren, 1865**<br>(Arctic Ocean)
–	Numerous long, cylindrical spines with blunt tips (Fig. 17E, F)<br><br>Cylindrical elytral macrotubercles with flattened, crown-like tip with undulate margin (appearing as coronate discs in top view) (Fig. 17C) and apically arborescent	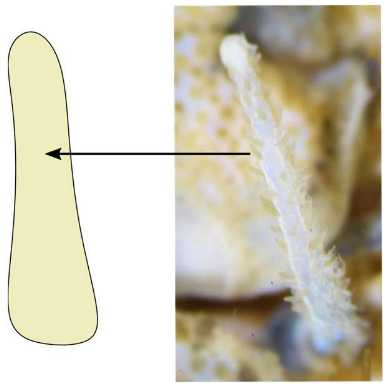	***Eunoe* cf. *oerstedi* CMC01**<br>(Alaska)

		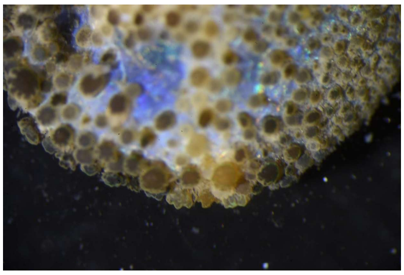	
6	Extra lobes present on elytrophores (Fig. 5D), dorsal tubercles (Fig. 5D), and cirrophores (Fig. 5A, D).<br><br>Notochaetae with subacute tips (Fig. 7C).<br><br>Elytra with apically arborescent macrotubercles (Fig. 8), never horn-like<br><br>Elytral macrotubercles semiglobular on large specimens (Fig. 8).<br><br>Nephridial papilla not visible, covered by nephridial sac (Figs 5C, 7E).<br><br>Body white, dorsal cirri white with brown spot (Fig. 5B, F)	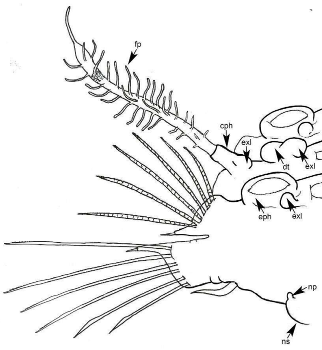<br>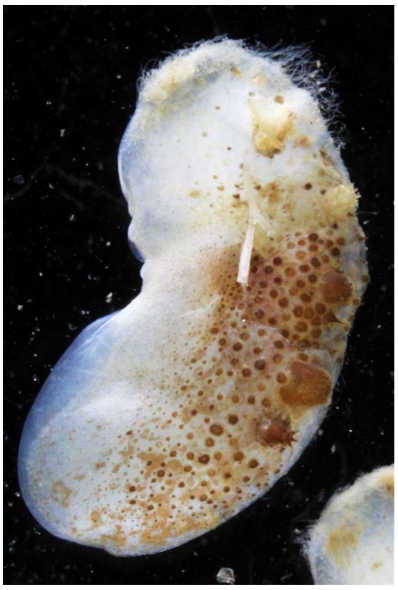	***Eunoe nodosa* (M. Sars, 1861)**<br>(North Atlantic, Arctic, and North Pacific Oceans)
–	Dorsal tubercles without extra lobes		**7**

7	Extra lobes present on elytrophores and cirrophores<br><br>Elytral macrotubercles cylindrical, ending distally in rosette-shaped apex with strongly lobate margin (“flower-like” heads with 5–8 petals in top view), and cylindrical macrotubercles with dentate crown at tip (Fig. 20B, C)	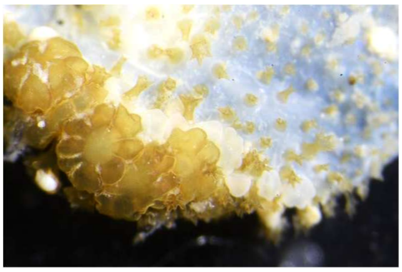	***Eunoe sentiformis*** **Uschakov, 1958** (Northwestern Pacific Ocean)
–	Extra lobes present on cirrophores<br><br>Elytral macrotubercles otherwise		**8**
8	All notochaetae with pointed tips<br><br>Elytral macrotubercles mostly arranged in a single row near posterior margin, with branched tips<br><br>Extra lobes on cirrophores present, on elytrophores absent	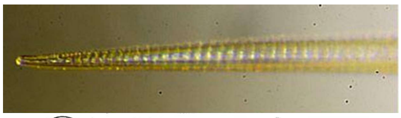<br>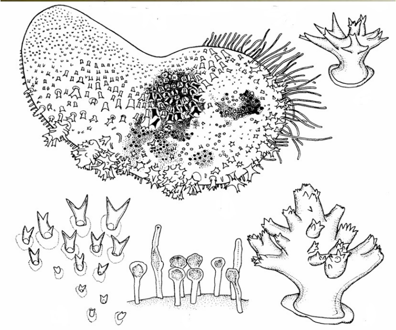<br>(from Imajima 1997)	***Eunoe spinosa*** **Imajima, 1997** (Japan)
–	Some notochaetae with truncate tips	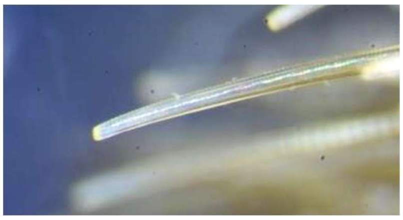	**9**

9	Elytral margin with short and scarce filiform papillae<br><br>Elytra with branching spiniform macrotubercles with sharp tips (Fig. 19A, B)<br><br>Notochaetae with truncate tips	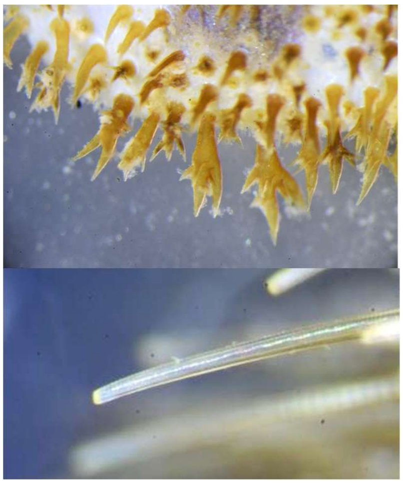	***Eunoe shirikishinai* (Imajima & Hartman, 1964)**<br>(Northwestern Pacific Ocean)
–	Elytral margin with dense fringe of filiform papillae		**10**
10	Elytral margin with dense fringe of filiform papillae and a distinct tuft of significantly longer papillae on mid-posterior margin (Fig. 21C, G)<br><br>Elytra with cylindrical to slightly clavate macrotubercles, apically coronate with small branches with rounded tips, forming crenulate apex (Fig. 21G)	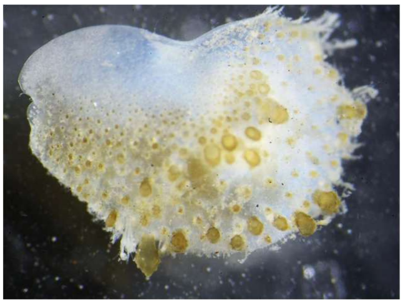	***Eunoe barbata* Moore, 1910**<br>(West coast of the USA)
–	Elytral margin with dense fringe of filiform papillae<br><br>Elytra with branching macrotubercles with sharp tips<br><br>Upper notochaetae with smooth, sharp tips; lower notochaetae with truncate, flattened tips	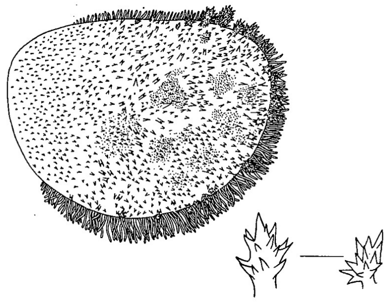<br>(from Ushakov 1982)	***Eunoe hozawai* Okuda, 1939**<br>(Northwestern Pacific Ocean)
